# Factors associated with the percentage of individuals who initiate and discontinue naltrexone as a relapse prevention pharmacotherapy in opioid use disorder: A systematic review, meta‐analysis and meta‐regression

**DOI:** 10.1111/add.70502

**Published:** 2026-06-12

**Authors:** Emmert Roberts, Elizabeth Sanderson, Thomas Santo, Matthew Hickman, Michael Farrell, Louisa Degenhardt

**Affiliations:** ^1^ National Addiction Centre, Institute of Psychiatry, Psychology and Neuroscience King's College London London UK; ^2^ National Drug and Alcohol Research Centre University of New South Wales Sydney Australia; ^3^ South London and the Maudsley NHS Foundation Trust London UK; ^4^ University of Bristol Bristol UK

**Keywords:** discontinuation, initiation, naltrexone, opioid use disorder, relapse prevention, systematic review

## Abstract

**Background and aims:**

Naltrexone is a pharmacotherapeutic option for relapse prevention in opioid use disorder (OUD); however, studies highlight low rates of initiation and high rates of discontinuation as limiting its effectiveness. We aimed to (1) estimate the percentage of individuals with OUD who (having undergone withdrawal and wish to remain abstinent) initiate and discontinue naltrexone and (2) examine participant‐ and study‐level factors that contribute to variation in initiation and discontinuation rates.

**Methods:**

We undertook a systematic review, random‐effects meta‐analysis and meta‐regression searching Medline, Embase, PsychINFO and CENTRAL from database inception to 19 February 2025 for studies of any design from any geographical region involving individuals with OUD eligible to receive naltrexone as a relapse prevention pharmacotherapy (i.e. those completing opioid withdrawal and wishing to remain abstinent). Measurements included the percentage of individuals who initiate or discontinue oral, long‐acting injectable depot or implantable formulations of naltrexone at 1, 3 or 6 months. Certainty was assessed using the GRADE framework.

**Results:**

Twenty‐two studies, including 124 016 individuals, reported initiation and 95 studies, including 16 969 individuals, reported discontinuation. The pooled percentage initiating oral naltrexone among those eligible was 60.3% [95% confidence interval (CI) = 38.9%–80.0%, 2014 participants, 15 studies] and depot was 18.2% (95% CI = 2.7%–42.5%, 57 383 participants, 4 studies). The pooled percentage discontinuing oral was 50.0% (95% CI = 41.9%–58.1%, 7340 participants, 34 studies) at 1 month, 61.3% (95% CI = 50.9%–71.2%, 2347 participants, 29 studies) at 3 months and 71.0% (95% CI = 57.3%–83.0%, 1889 participants, 19 studies) at 6 months. The pooled percentage discontinuing depot was 26.1% (95% CI = 19.5%–33.3%, 3589 participants, 33 studies) at 1 month, 46.7% (95% CI = 38.4%–55.1%, 3302 participants, 33 studies) at 3 months and 60.0% (95% CI = 43.2%–75.8%, 3071 participants, 22 studies) at 6 months. Statistically significantly higher percentages initiated oral naltrexone if it was the only offered pharmacotherapy (meta‐regression coefficient 33.6%, 95% CI = 8.1%–59.2%, *P* = 0.014) and statistically significantly lower percentages discontinued oral naltrexone at 3 and 6 months if administration was supervised (meta‐regression coefficient −18.6%, 95% CI = −36.6% to −1.0%, *P* = 0.043 and −27.3%, 95% CI = −50.1% to −4.4%, *P* = 0.022, respectively). There was no clear evidence that study setting (i.e. if the study was conducted in routine clinical care or an investigational setting) substantially explained or contributed to the variation in any estimates. All outcomes were very low certainty.

**Conclusions:**

Very low certainty evidence suggests that, among people with opioid use disorder who have undergone withdrawal and wish to remain abstinent, a substantial percentage are willing to initiate naltrexone with marked early discontinuation.

## INTRODUCTION

The challenges of preventing return to extra‐medical opioid use following a period of abstinence in people with opioid use disorder (OUD) contribute to the continuing epidemic of addiction and overdose across the globe. A low proportion of individuals optimally complete treatment for OUD and whereas opioid agonist treatment (OAT) is highly effective if individuals are willing to remain on treatment, a substantial number either do not wish to or find it challenging to do so [1, 2, 3]. Commonly stated reasons include the desire to be ‘opioid‐free’ and side‐effects from opioid agonists including drowsiness and dizziness [4]. The opioid antagonist naltrexone is licensed as a relapse prevention pharmacotherapy for the subpopulation of individuals with OUD who are currently opioid free and wish to maintain abstinence from extra‐medical opioid use; it has been shown to reduce the risk of return to extra‐medical opioid use in those who are compliant with treatment and is typically commenced 7 to 10 days following opioid cessation to allow sufficient time to elapse and reduce the risk of precipitated withdrawal. [5, 6, 7, 8].

The literature on naltrexone frequently reports concerns that it is ‘underutilised’ with several reports suggesting both limited availability within treatment programmes and limited awareness of it as a treatment option [4, 9]. However, since its inception naltrexone research has also consistently highlighted substantial reluctance among people with OUD to consider or continue it as a treatment option, with low rates of initiation and high rates of discontinuation frequently reported as limiting its potential effectiveness [10]. Multiple international treatment guidelines echo these concerns often limiting recommendation of its use to specific populations with high levels of motivation to maintain abstinence [11, 12]. However, the majority of supporting evidence typically describes trends in dispensing data, comparing the number of naltrexone prescriptions to those of methadone or buprenorphine (which are typically far larger in magnitude) [13], with often only small historical single‐site studies cited reporting the percentage of individuals who initiate or discontinue among those offered naltrexone who are eligible to receive it [13, 14, 15]. Although several qualitative studies have attempted to explore reasons why individuals may or may not choose to use naltrexone [16, 17], there have been limited attempts to quantitatively synthesise the evidence to establish initiation and discontinuation rates or evaluate any factors that may impact on the number of individuals initiating or discontinuing treatment. Time spent actively taking naltrexone has a lower mortality risk when compared to non‐pharmacologically assisted abstinence, and discontinuation is associated with an elevated risk of relapse and overdose [18]. Therefore, a more complete understanding of the factors associated with both initiation and discontinuation may assist in targeting pharmacotherapy, provide a more robust evidence base on which to base current prescribing guidance, and ultimately help mitigate the risk of relapse and overdose in individuals with OUD who are opioid free and wish to maintain abstinence.

To address these gaps, for each available formulation of naltrexone (i.e. oral, long‐acting injectable depot and implantable) we aimed to conduct a systematic review and meta‐analysis to (1) estimate the percentage of individuals who initiate naltrexone as a relapse prevention pharmacotherapy among those offered naltrexone who are eligible to receive it (i.e. the number of individuals who take at least one dose of naltrexone compared to the number of individuals offered naltrexone who are currently opioid free, having completed self or medically assisted opioid withdrawal from any opioid agonist); (2) estimate the percentage of individuals who discontinue naltrexone among those who used it for relapse prevention (i.e. the proportion of people who stop taking naltrexone among those who receive at least one dose); and (3) use meta‐regression to examine what participant‐ and study‐level factors are associated with initiation and discontinuation.

## METHODS

This study is reported according to the Preferred Reporting Items for Systematic Reviews and Meta‐Analyses (PRISMA) and Meta‐analysis of Observational Studies in Epidemiology (MOOSE) guidelines [19, 20]. The study did not require ethical approval and the complete review protocol was pre‐registered on PROSPERO (CRD42025646642).

### Search strategy

We searched Medline, Embase, PsychINFO and the Cochrane Central Register of Controlled Trials (CENTRAL) from database inception to 19 February 2025. The complete search strategies can be found as Figure S1 in the online supplementary material.

### Study selection

We included studies of any design that reported either (1) a calculable percentage of individuals who initiated naltrexone as a relapse prevention pharmacotherapy for OUD among those eligible to receive it (i.e. the number of individuals who received at least one dose of naltrexone compared to the number of individuals offered naltrexone who were currently opioid free having completed self or medically assisted opioid withdrawal from any opioid agonist) or (2) a calculable percentage of individuals discontinuing naltrexone as a relapse prevention pharmacotherapy for OUD at 1, 3 or 6 months following initiation (i.e. the number of individuals who stopped taking naltrexone compared to the number of individuals who received at least one dose). Oral formulation discontinuation estimates were based on the number of individuals reported as having ceased tablet consumption (e.g. through self‐report, pill counts, riboflavin urine bioassays *etc*.), depot discontinuation estimates were based on the reported number of injections received and implant discontinuation based on the reported number of implants received and the number removed before the end of the period of naltrexone coverage. Two authors (E.R. and E.S.) initially assessed titles and abstracts and subsequently reviewed the full text of the remaining articles for inclusion. Any discrepancy was resolved by discussion, and where agreement could not be reached a third author (T.S.) was consulted. All relevant references were checked for additional citations.

### Data extraction

Two authors (E.R. and E.S.) independently extracted data from all eligible studies using a standardised data extraction spreadsheet. In the case of incomplete reporting of data, we searched studies' online supplementary appendices, clinical trial registries and contacted authors as necessary. We additionally extracted participant‐ and study‐level factors that may explain the variation in estimates of the percentage of individuals initiating or discontinuing naltrexone. Complete descriptions of extracted factors can be found in Figure S2. Participant‐level factors included individual's mean age, the percentage of participants who were female, the percentage of participants who were employed, the level of motivation to maintain abstinence from extra‐medical opioid use, the type of opioid used by the majority of participants before withdrawal and if recruited participants were criminal justice offenders or healthcare professionals (as these populations have historically been considered to have potentially differential rates of naltrexone initiation and/or discontinuation) [21]. Study‐level factors include the study setting [i.e. investigational studies (studies conducted in a research context according to a study protocol) or studies of records taken from routine clinical care—this grouping reflecting that naltrexone was not always the studied exposure in included clinical trials] [5], year and country of data collection, the type of detoxification [i.e. in‐patient, outpatient or ‘rapid’ (i.e. conducted under anaesthesia or sedation), the dose and formulation of naltrexone (i.e. oral, long‐acting injectable depot or implantable)], whether naltrexone was reportedly administered under supervision, whether naltrexone was the only pharmacotherapy reported to be offered at the point of individuals completing opioid withdrawal and whether any psychosocial support, or any psychosocial support specifically designed to increase naltrexone adherence, was reportedly provided during receipt of naltrexone. Where studies reported percentages of individuals initiating or discontinuing naltrexone in two or more unique populations that differed in one of the study‐level factors (e.g. an investigational study comparing treatment as usual to a psychosocial intervention designed to improve naltrexone adherence or a study of routine clinical care comparing individuals on oral or implantable formulations) these were extracted and reported separately.

### Certainty assessment

Following best practice as laid out in the Cochrane Handbook, the certainty of each pooled prevalence estimate was assessed using the Grading of Recommendations Assessment, Development and Evaluation (GRADE) framework. [22] Each estimate is given a rating of high, moderate, low or very low certainty based on scores in five domains: risk of bias, inconsistency, indirectness, imprecision and publication bias. The default certainty score is low for outcomes based on observational data and high for outcomes based on randomised data, which can then be up‐ or down‐graded according to the certainty of evidence for each estimate. Risk of bias was assessed using a quality assessment tool adapted from the Newcastle‐Ottawa Scale, available in Figure S3 and, where sufficient estimates (*n* ≥ 10) were available, publication bias was assessed using funnel plots and Egger's test for small study effects [23, 24]. The complete GRADE scoring criteria are available in Figure S4. Two reviewers (E.R. and E.S.) independently estimated the GRADE certainty of each outcome, any discrepancy was resolved by discussion and where agreement could not be reached a third author (T.S.) was consulted.

### Statistical analysis

Pooled estimates were calculated for the percentage of individuals initiating or discontinuing naltrexone stratified *a priori* by each naltrexone formulation (i.e. oral, long‐acting injectable depot or implantable) and subgrouped by study setting. The true percentages were likely to vary between studies because of a variety of factors, including differences in studied population and diagnostic definitions. As such, a high degree of heterogeneity was anticipated and we chose *a priori* to perform random‐effects meta‐analysis [25]. Percentages are bounded by 0 and 100 and some reported estimates may be close to these boundaries. As a normal approximation can break down at these extremes we used the Freeman‐Tukey double arcsine transformation as a method of stabilising the variances [26]. Heterogeneity was examined through complete case exploratory meta‐regression if there were sufficient numbers of percentage estimates (*n* ≥ 10) [24]. Factors were sequentially entered as univariable explanatory variables to assess their contribution to the variation in the percentage estimates. We compared τ^2^ (the between‐study variance) and *I* ^2^ values from the meta‐analysis with the τ^2^ and *I* ^2^ values, and the adjusted *R* ^2^ (the percentage of variation in the percentage estimates explained by the explanatory variable), from each univariable meta‐regression. Where explanatory variables were continuous or binary, results were reported to show how the pooled percentage point difference estimate changes with each unit increase of the explanatory variable. For categorical variables, results were reported to show how the pooled percentage estimates differ across categories. If an explanatory variable demonstrated a significant association with the percentage of individuals initiating or discontinuing naltrexone, and there was a reduction in the τ^2^ and *I* ^2^, and an *R* ^2^ >0, in univariable meta‐regression, we planned to introduce these variables using a forward approach into a multi‐variable meta‐regression model, provided this would not breach the rules of data sparsity and result in overparameterisation (in this case defined as fewer than 10 estimates for each covariate). All analyses were conducted in STATA IC version 17, with the significance level set at 0.05.

## RESULTS

The search generated 3686 unique results, and 104 additional references were identified from citation searching leading to a total of 3790. We examined 635 full texts and included a total of 97 studies. A total of 538 studies were excluded. Common reasons for exclusion included that studies did not report data enabling calculation of the percentage of individuals initiating or discontinuing naltrexone (*n* = 103), studies reported retention in treatment, as measured by clinic attendance, or relapse to extra‐medical opioid use, and not the rate of naltrexone discontinuation (*n* = 82) or studies reported qualitative data exploring perceptions of naltrexone as a pharmacotherapy (*n* = 12). Full reasons for each study's exclusion can be found in Table S1 and the PRISMA diagram, describing the complete study selection, can be found as Figure S5.

A description of all included studies can be found in Table S2. Sixty‐one (63%) studies were conducted in the United States, eight (8%) in Australia, six in Norway (6%) with the remainder conducted in Canada, Germany, India, Iran, Israel, Italy, Mauritius, the Netherlands, Russia, Turkey, Ukraine and the United Kingdom. Overall, 45 (46%) studies contained data reporting on oral formulations of naltrexone, 42 (43%) on long‐acting injectable depot formulations and six (6%) on implantable formulations. Sixty (62%) studies were investigational studies and 37 (38%) were studies conducted in routine clinical care. Twenty‐two studies (23%), including a total of 124 016 individuals, reported data enabling calculation of the percentage of individuals initiating naltrexone and 95 (98%) studies, including a total of 16 969 individuals, reported data enabling calculation of the percentage of individuals discontinuing naltrexone. Sixty‐nine (71%) studies reported discontinuation at 1 month, 65 (67%) at 3 months and 44 (45%) at 6 months. Ten studies (10%) reported on initiation and discontinuation within the same sample. Only four studies (4%) formally assessed individuals' motivation to maintain abstinence from extra‐medical opioid use and, where reported, the majority of studies (82%) included individuals who were using extra‐medical or illicit opioids before undergoing opioid withdrawal.

### Initiation

**FIGURE 1:**
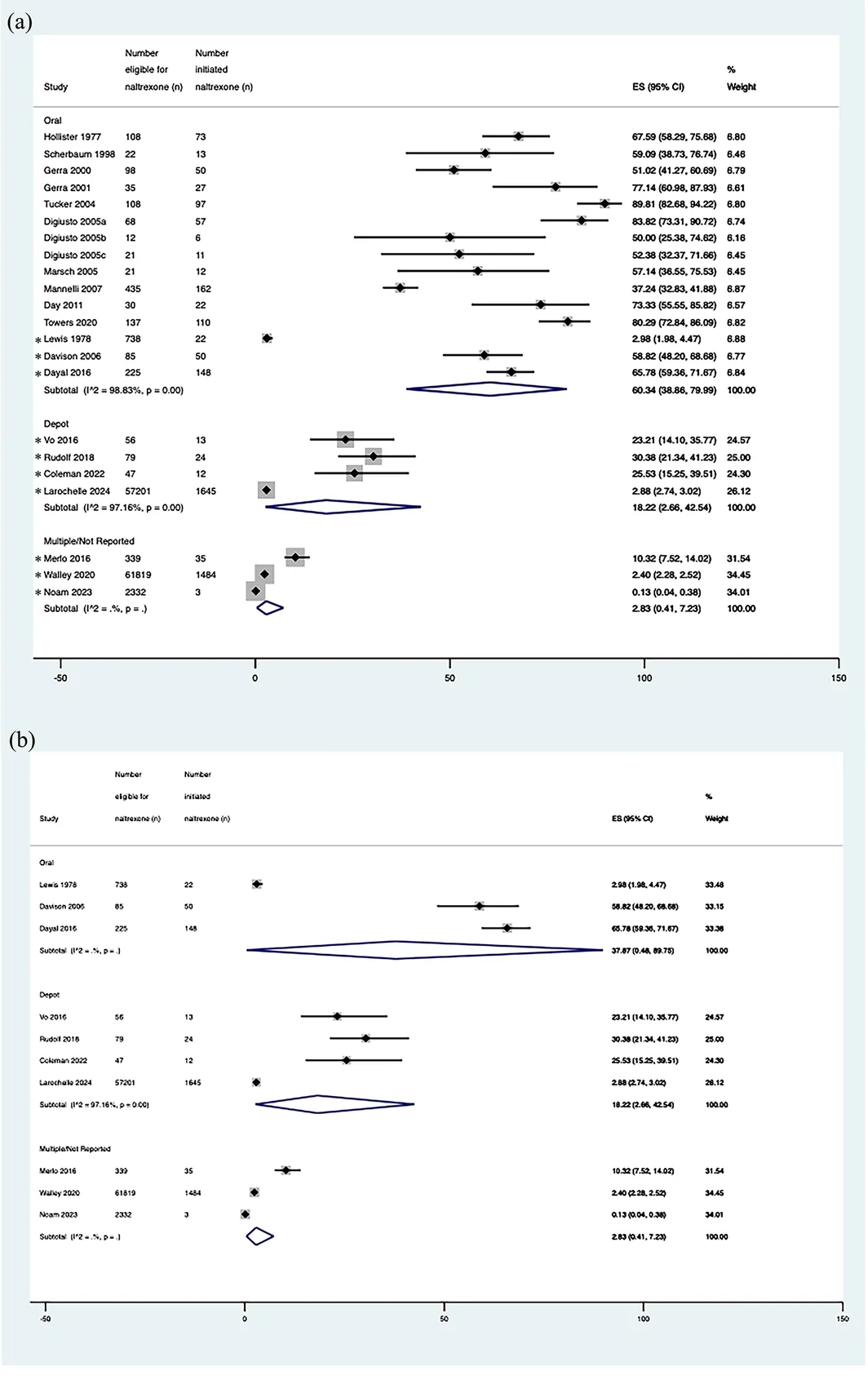
Forest plots of the (a) overall pooled percentages of individuals who initiated naltrexone as a relapse prevention pharmacotherapy for opioid use disorder among those who were eligible to receive it (i.e. people who have completed opioid withdrawal and wish to maintain abstinence from extra‐medical opioid use) and (b) pooled percentages restricted to those studies conducted in routine clinical care. *Studies conducted in routine clinical care [subgroup analyses presented as Figure 1 (b)].

The pooled percentage of individuals who initiated oral naltrexone among people who had completed opioid withdrawal and wished to maintain abstinence was 60.3% (95% CI = 38.9%–80.0%) and for long‐acting injectable depot formulations was 18.2% (95% CI = 2.7%–42.5%). No studies reported a calculable percentage of individuals initiating implantable naltrexone [Figure 1 (a)]. There was substantial heterogeneity with all *I* ^2^ values greater than 97%. There were no significant differences in the subgroup pooled percentage estimates by study setting with the pooled percentage of individuals initiating naltrexone in routine clinical care estimated as 37.9% (95% CI = 0.5%–89.8%) for oral formulations and 18.2% (2.7%–42.5%) for long‐acting injectable depot formulations. Forest plots including only those studies conducted in routine clinical care are available as Figure 1 (b), with forest plots for only those studies conducted in investigational settings available in Figure S6.

Exploratory meta‐regression was only possible for oral formulations. Univariable meta‐regression demonstrated that one factor was significantly associated with the percentage of individuals initiating oral naltrexone. When compared to individuals who were offered multiple relapse prevention pharmacotherapy options (e.g. naltrexone, methadone or buprenorphine), if individuals were offered only naltrexone the percentage point difference of individuals initiating naltrexone increased by +33.6% (95% CI = +8.1% to +59.2%, *P* = 0.014) (Table 1). There was no clear evidence that any other variables appeared to substantially explain or contribute to the variation in initiation estimates during meta‐regression.

**TABLE 1:** Univariable meta‐regression of individual and study‐level factors associated with the percentage of individuals initiating oral naltrexone as a relapse prevention pharmacotherapy in opioid use disorder.

	Percentage estimates (*n*)	Individuals (*n*)	Percentage	LCI	UCI	τ^2^	*I* ^2^ (%)	*P* value	Adjusted *R* ^2^ (%)
	15	2413	60.3	38.9	80.0	0.676	98.8		
	Percentage estimates (*n*)	Individuals (*n*)	β	LCI	UCI	τ^2^	*I* ^2^ (%)		
Univariable									
Mean age of individuals (continuous)	3	565	−0.08	−0.44	0.27	0.000	0.0	0.211	100.0
Percentage of female individuals (continuous)	5	1401	0.004	−0.02	0.03	0.078	93.0	0.659	24.8
Median year of data collection (continuous)	7	1304	0.02	−0.01	0.03	0.020	80.6	0.413	15.1
Total Newcastle‐Ottawa scale quality score (continuous)	15	2413	−0.01	−0.15	0.14	0.054	93.5	0.945	7.2
Study setting [binary: routine versus investigational (ref)]	15	2413	−0.25	−0.56	0.07	0.042	89.7	0.111	16.1
Was naltrexone the only relapse prevention pharmacotherapy offered? [binary: yes versus no (ref)]	15	2413	0.34	0.08	0.59	0.029	82.6	0.014	42.6
Type of opioid used before withdrawal and naltrexone initiation [binary: OAT versus extra‐medical/illicit use (ref)]	11	840	0.15	−0.05	0.34	0.000	0.0	0.126	100.0
Type of detoxification [categorical: rapid, outpatient versus in‐patient (ref)]	12	1868	0.05	−0.47	0.58	0.047	92.6	0.823	0.7
			0.24	−0.23	0.72			0.275	

Abbreviations: LCI, lower 95% confidence interval; UCI, upper 95% confidence interval.

### Discontinuation

**FIGURE 2:**
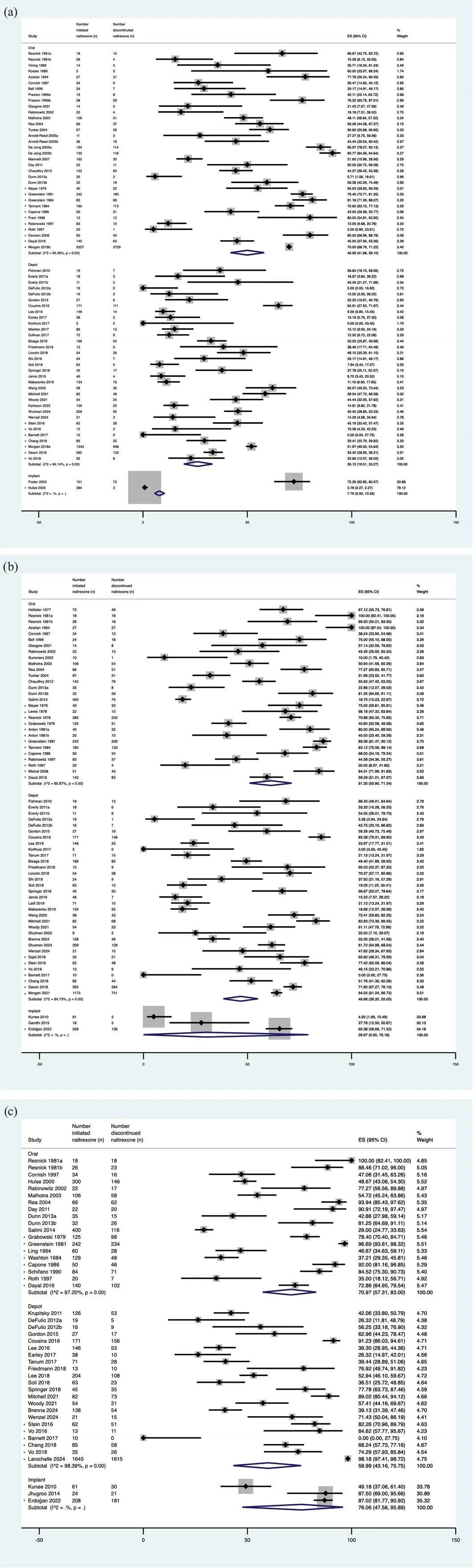
Forest plots of the pooled percentage of individuals who discontinued naltrexone as a relapse prevention pharmacotherapy for opioid use disorder at (a) 1 month, (b) 3 months and (c) 6 months following initiation.*Studies conducted in routine clinical care (subgroup analyses available in Figure S7).

The pooled percentage of individuals who discontinued oral naltrexone at 1 month was 50.0% (95% CI = 41.9%–58.1%), at 3 months it was 61.3% (95% CI = 50.9%–71.2%) and at 6 months it was 71.0% (95% CI = 57.3%–83.0%). The pooled percentage of individuals who discontinued long‐acting injectable depot naltrexone at 1 month was 26.1% (95% CI = 19.5%–33.3%), at 3 months it was 46.7% (95% CI = 38.4%–55.1%) and at 6 months it was 60.0% (95% CI = 43.2%–75.8%). The pooled percentage of individuals who discontinued implantable naltrexone at 1 month was 7.8% (95% CI = 5.5%–10.4%), at 3 months it was 30.0% (95% CI = 0.0%–79.2%) and at 6 months it was 76.1% (95% CI = 47.6%–95.9%) (Figure 2). There was substantial heterogeneity with all *I* ^2^ values greater than 94%. There were no significant differences in the subgroup pooled percentage estimates by study setting with forest plots including only those studies conducted in routine clinical care in Figure S7. A plot showing the percentage of individuals initiating and discontinuing naltrexone in the 10 studies that contained data on both initiation and discontinuation within the same sample can be found in Figure S8.

Exploratory meta‐regression was possible for oral and long‐acting injectable formulations. Univariable meta‐regression demonstrated that one factor was significantly associated with the percentage of individuals discontinuing both oral and long‐acting injectable formulations at 1 month. When compared to individuals who did not receive a specific psychosocial intervention designed to increase naltrexone adherence, if individuals did receive a specific psychosocial intervention proportionally fewer individuals discontinued oral naltrexone at 1 month by a percentage point difference of −35.3% (95% CI = −68.6% to −2.1%, *P* = 0.038) (Table 2) and the percentage discontinuing long‐acting injectable formulations at 1 month decreased by −22.6% (95% CI = −44.5% to −1.0%, *P* = 0.044) (Table 3). Univariable meta‐regression demonstrated that one factor was significantly associated with the percentage of individuals discontinuing oral naltrexone at 3 and 6 months. When compared to individuals who reportedly did not have administration of their oral naltrexone supervised, if naltrexone was supervised the percentage point difference of individuals discontinuing naltrexone decreased by −18.6% (95% CI = −36.6% to −1.0%, *P* = 0.043) at 3 months and by −27.3% (95% CI = −50.1% to −4.4%, *P* = 0.022) at 6 months. There was no significant association of the receipt of a specific psychosocial intervention designed to increase naltrexone adherence with the percentage of individuals discontinuing any formulation of naltrexone at either 3 or 6 months. Complete meta‐regression results for discontinuation at 3 and 6 months can be found in the online supplementary material as Tables S3–S10. There was no clear evidence that any other variables appeared to substantially explain or contribute to the variation in discontinuation estimates during meta‐regression.

**TABLE 2:** Univariable meta‐regression of individual and study‐level factors associated with the percentage of individuals discontinuing oral naltrexone as a relapse prevention pharmacotherapy in opioid use disorder 1 month following initiation.

	Percentage estimates (*n*)	Individuals (*n*)	Percentage	LCI	UCI	τ^2^	*I* ^2^ (%)	*P* value	Adjusted *R* ^2^ (%)
	34	7426	50.0	41.8	58.1	0.190	95.4		
	Percentage estimates (*n*)	Individuals (*n*)	β	LCI	UCI	τ^2^	*I* ^2^ (%)		
Univariable									
Mean age of individuals (continuous)	17	1148	−0.02	−0.04	0.01	0.030	66.1	0.180	12.3
Percentage of female individuals (continuous)	18	6552	−0.003	−0.01	0.003	0.027	71.3	0.316	14.1
Percentage of employed individuals (continuous)	10	692	0.001	−0.004	0.007	0.019	56.6	0.615	21.2
Median year of data collection (continuous)	19	6729	−0.01	−0.02	0.008	0.048	81.5	0.421	4.0
Total Newcastle‐Ottawa scale quality score (continuous)	34	7426	0.06	−0.01	0.13	0.039	73.4	0.068	7.3
Study setting [binary: routine versus investigational (ref)]	34	7426	0.09	−0.09	0.28	0.043	75.0	0.314	0.1
Type of opioid used before withdrawal and naltrexone initiation [binary: OAT versus extra‐medical/illicit use (ref)]	18	768	−0.11	−0.51	0.28	0.020	43.0	0.545	4.0
Were the recruited individuals healthcare professionals? [binary: yes versus no (ref)]	34	7426	−0.48	−0.93	0.14	0.040	78.4	0.122	5.2
Were the recruited individuals criminal justice offenders? [binary: yes versus no (ref)]	34	7426	−0.26	−0.81	0.29	0.042	78.8	0.344	0.4
Was the naltrexone reportedly administered under supervision? [binary: yes versus no (ref)]	34	7426	−0.05	−0.25	0.15	0.044	77.5	0.613	3.7
Was psychosocial therapy reportedly provided to people on naltrexone [binary: yes versus no (ref)]	34	7426	−0.02	−0.22	0.18	0.044	76.9	0.855	4.3
Was a specific psychosocial intervention designed to increase naltrexone adherence delivered? [binary: yes versus no (ref)]	34	7426	−0.35	−0.69	−0.02	0.036	76.6	0.038	14.7

Abbreviations: LCI, lower 95%confidence interval; UCI, upper 95% confidence interval.

**TABLE 3:** Univariable meta‐regression of individual and study‐level factors associated with the percentage of individuals discontinuing long‐acting injectable depot naltrexone as a relapse prevention pharmacotherapy in opioid use disorder 1 month following initiation.

	Percentage estimates (*n*)	Individuals (*n*)	Percentage	LCI	UCI	τ^2^	*I* ^2^ (%)	*P* value	Adjusted *R* ^2^ (%)
	33	3.675	26.1	19.5	33.3	0.166	94.1		
	Percentage estimates (*n*)	Individuals (*n*)	β	LCI	UCI	τ^2^	*I* ^2^ (%)		
Univariable									
Mean age of individuals (continuous)	15	1234	−0.01	−0.02	0.01	0.029	69.1	0.452	9.6
Percentage of female individuals (continuous)	16	2577	0.002	−0.004	0.01	0.029	76.5	0.500	1.0
Percentage of employed individuals (continuous)	10	900	−0.003	−0.01	0.004	0.026	54.4	0.351	13.9
Median year of data collection (continuous)	32	3589	0.002	−0.02	0.03	0.024	70.7	0.882	5.2
Total Newcastle‐Ottawa scale quality score (continuous)	33	3.675	0.03	−0.03	0.09	0.023	71.7	0.316	2.2
Study setting [binary: routine versus investigational (ref)]	33	3.675	0.06	−0.11	0.24	0.022	65.3	0.468	0.2
Type of opioid used before withdrawal and naltrexone initiation [binary: OAT versus extra‐medical/illicit use (ref)]	21	1098	−0.16	−0.54	0.21	0.022	51.6	0.370	0.5
Were the recruited individuals healthcare professionals? [binary: yes versus no (ref)]	33	3.675	−0.18	−0.63	0.28	0.023	71.2	0.435	0.7
Were the recruited individuals criminal justice offenders? [binary: yes versus no (ref)]	33	3.675	0.02	−0.18	0.22	0.024	70.6	0.849	5.5
Was psychosocial therapy reportedly provided to people on naltrexone [binary: yes versus no (ref)]	33	3.675	−0.05	−0.20	0.10	0.023	69.7	0.472	3.5
Was a specific psychosocial intervention designed to increase naltrexone adherence delivered? [binary: yes versus no (ref)]	33	3.675	−0.23	−0.45	−0.01	0.019	67.6	0.044	13.9

Abbreviations: LCI, lower 95%confidence interval; UCI, upper 95% confidence interval.

### Certainty

There was significant evidence of publication bias for all initiation and discontinuation outcomes, funnel plots available in Figures S9–S12. The overall GRADE certainty score was very low for all outcomes. The full clinical evidence profiles are available in Tables S3–S6.

## DISCUSSION

Approximately three‐fifths of individuals with OUD who have completed opioid withdrawal and wish to maintain abstinence initiate at least one dose of oral naltrexone, and one‐fifth initiate long‐acting injectable depot naltrexone when offered it for relapse prevention. The only factor significantly impacting on oral initiation rates being a reduction in naltrexone initiation if the eligible cohort was concurrently offered the option of an opioid agonist. Half of individuals taking oral naltrexone discontinue by 1 month and almost three‐quarters discontinue by 6 months compared to a quarter who do not receive their second depot injection and three‐fifths who have ceased receiving depot injections at 6 months. Although early discontinuation rates are significantly reduced if individuals are provided with specific psychosocial interventions designed to increase naltrexone adherence, these effects do not appear to persist beyond 1 month, the only factor significantly reducing rates of oral discontinuation thereafter being administration under supervision.

### Other evidence

Previous studies have demonstrated that a substantial number of individuals with OUD are potentially willing to consider a pharmacotherapy for relapse prevention [27]. Discontinuation of naltrexone has also been shown to have a significantly elevated risk of subsequent overdose compared to those discontinuing opioid agonists [18, 28], and as such, collaborative clinician and patient discussions need to be aware of subsequent risks of differing pharmacotherapeutic strategies at the point of prescribing for relapse prevention. We demonstrate that the simultaneous offer of naltrexone and opioid agonists significantly reduces the percentage of people initiating naltrexone, highlighting the importance of offering the full suite of available pharmacotherapy to eligible individuals to ensure people can make an informed choice about ongoing care. The ability to offer naltrexone as a treatment option is, however, necessarily predicated on it being available and there being no restrictions on its prescription [29]. Lack of availability of naltrexone in some regions may limit patient choice and, in the case of licenced long‐acting depot formulations, contribute to increased early discontinuation [5].

Several treatment guidelines suggest that the use of naltrexone should be considered (1) only in conjunction with psychosocial interventions; and (2) limited to populations with a high level of motivation to maintain abstinence from extra‐medical opioid use [11, 12]. Although the overall certainty of evidence was rated as very low, this study does not support these recommendations given (1) there was no clear evidence of a statistically significant impact of the provision of psychosocial interventions on rates of discontinuation at any studied time point, other than specific psychosocial interventions designed to increase naltrexone compliance at 1 month; and (2) only four included studies reported formal measurement of motivation, only two reporting that this was high in the majority of individuals [30, 31], Recommendations for restriction of use to motivated cohorts have typically relied on studies from specific populations thought to be inherently more motivated because of additional factors (e.g. criminal justice offenders under probation schemes or healthcare professionals at risk of loss of employment) [32, 33], however, there was no clear evidence that discontinuation rates appeared to significantly differ within these populations at any time point. Few guidelines recommend the consideration of healthcare professional or family member administration of oral formulations under supervision, however, for individuals willing to submit to supervision this may significantly reduce discontinuation. Previous systematic reviews have examined the effectiveness of psychosocial interventions as an adjunct to naltrexone therapy (e.g. contingency management, family therapy, *etc*.) with mixed results attributed to different treatment modalities and studied populations. [34] Although the majority of included studies were conducted in investigational settings, almost all studies contributing data to the initiation outcome allowed participants a free choice of whether to commence naltrexone as a relapse prevention pharmacotherapy (e.g. investigational studies included clinical trials that compared different methods of detoxification and subsequently offered naltrexone to participants who had successfully competed opioid withdrawal and wished to maintain abstinence). Nevertheless, individuals are likely to behave differently in investigational settings compared to routine clinical care, and as such exploration of potential differences between these settings is warranted, it was reassuring there was no clear evidence that study setting was able to substantially explain or contribute to the variation in any estimates.

The study has several strengths including a broad search strategy and a pre‐registered protocol to capture studies of any design. We acknowledge several limitations, estimates of the percentage of individuals initiating or discontinuing naltrexone were not typically the primary aim of a study, and as such, were frequently buried in the main body of article texts with often no baseline variable description of the cohorts either eligible to receive or having received naltrexone. This limited the evidence on which participant‐level factors may demonstrate an increased likelihood of naltrexone compliance. Similarly, many studies lacked detail in their definition of the outcome retention in treatment, with lack of clinic attendance, lack of relapse to extra‐medical opioid use and treatment discontinuation often used interchangeably [35]. As such, these factors could have led to some studies being missed at the inclusion stage. Additional limitations included the limited literature on initiation and discontinuation of naltrexone implants. Despite claims that implants may provide an elegant solution to the issue of high discontinuation for other formulations [36], many studies did not report the intended duration of implant coverage, the numbers receiving subsequent implants or the number of implants removed, preventing robust initiation and discontinuation conclusions. Indeed, many studies reported on the use of unlicensed implant products, the use of which have subsequently been restricted [36]. Almost two‐thirds of included studies were conducted in the United States, with others reported in jurisdictions in which OAT is unavailable [37], likely impacting on the rates of naltrexone usage. Although this may limit the generalisability of results, English language was not an applied restriction in a bid to include data from the broadest geographical footprint.

Although there is general awareness that the percentage of people initiating naltrexone, among those eligible, is ‘low’ and discontinuing naltrexone is ‘high’, this terminology is often discussed in the literature without quantification. We have attempted to provide this quantification and are of the opinion that our results represent a smaller magnitude of ‘underutilisation’ than is currently anecdotally assumed [38]. There will always be a cohort of individuals with OUD who do not wish to take opioid agonists and, given the substantial percentage of opioid free individuals who are willing to, and do, initiate naltrexone, in people wishing to maintain abstinence and considering relapse prevention pharmacotherapy a collaborative patient and clinician decision should consider the potential risks, benefits and side effects alongside discussion of all pharmacotherapeutic treatment options for this group. Use of depot formulations or specific psychosocial interventions may reduce early discontinuation, and it may be appropriate to discuss and offer the option of oral tablet administration under supervision. Many individuals do continue to take naltrexone at 6 months post initiation, but any lapse to extra‐medical opioid use or naltrexone discontinuation should trigger an urgent clinical review and consideration of the most appropriate ongoing treatment.

## AUTHOR CONTRIBUTIONS

**Emmert Roberts:** Conceptualization (lead); data curation (lead); formal analysis (lead); funding acquisition (lead); investigation (lead); methodology (lead); project administration (lead); resources (lead); writing—original draft (lead). **Elizabeth Sanderson:** Investigation (equal); resources (equal); writing—review and editing (equal). **Thomas Santo Jr.:** Investigation (equal); resources (equal); writing—review and editing (equal). **Matthew Hickman:** Conceptualization (equal); investigation (equal); resources (equal); supervision (equal); validation (equal); writing—review and editing (equal). **Michael Farrell:** Conceptualization (equal); investigation (equal); resources (equal); supervision (equal); validation (equal); writing—review and editing (equal). **Louisa Degenhardt:** Conceptualization (equal); investigation (equal); resources (equal); supervision (equal); validation (equal); writing—review and editing (equal).

## DECLARATION OF INTERESTS

All authors have completed the ICJME Unified Competing Interest form (available on request from the corresponding author) and declare: no support from any organisation for the submitted work; no financial relationships with organisations that might have an interest in the submitted work in the previous 3 years, no other relationships or activities that could appear to have influenced the submitted work.

## Supporting information


**Figure S1:** Search strategies.
**Figure S2:** Extracted variable definitions.
**Figure S3:** Adapted Newcastle‐Ottawa checklist.
**Figure S4:** GRADE certainty assessment.
**Figure S5:** PRISMA Flow Diagram.
**Figure S6:** Forest plot of the pooled proportion of individuals who initiated naltrexone as a relapse prevention pharmacotherapy for opioid use disorder among those who were eligible to receive it (i.e., people who have completed opioid withdrawal and wish to maintain abstinence from extra‐medical opioid use) in the subgroups of studies conducted in i) routine clinical care and ii) investigational settings.
**Figure S7:** Forest plots of the pooled proportion of individuals who discontinued naltrexone as a relapse prevention pharmacotherapy for opioid use disorder at a) one‐month, b) three‐months and c) six‐months following initiation in the subgroups of studies conducted in i) routine clinical care and ii) investigational settings.
**Figure S8:** The proportion of individuals initiating and discontinuing naltrexone in the ten studies containing data on both initiation and discontinuation within the same sample.
**Figure S9:** Funnel plot of estimates of proportion of individuals initiating naltrexone.
**Figure S10:** Funnel plot of estimates of proportion of individuals discontinuing naltrexone at one month following initiation.
**Figure S11:** Funnel plot of estimates of proportion of individuals discontinuing naltrexone at three months following initiation.
**Figure S12:** Funnel plot of estimates of proportion of individuals discontinuing naltrexone at six months following initiation.
**Table S1:** Excluded Studies.
**Table S2:** Description of included studies.
**Table S3:** GRADE clinical evidence profile for estimates of the proportion of individuals initiating naltrexone.
**Table S4:** GRADE clinical evidence profile for pooled estimates of the proportion of individuals discontinuing naltrexone at one month following initiation.
**Table S5:** GRADE clinical evidence profile for pooled estimates of the proportion of individuals discontinuing naltrexone at three months following initiation.
**Table S6:** GRADE clinical evidence profile for pooled estimates of the proportion of individuals discontinuing naltrexone at six months following initiation.
**Table S7:** Univariable meta‐regression of individual and study‐level factors associated with the proportion of individuals discontinuing oral naltrexone as a relapse prevention pharmacotherapy in opioid use disorder at three months following initiation.
**Table S8:** Univariable meta‐regression of individual and study‐level factors associated with the proportion of individuals discontinuing oral naltrexone as a relapse prevention pharmacotherapy in opioid use disorder at six months following initiation.
**Table S9:** Univariable meta‐regression of individual and study‐level factors associated with the proportion of individuals discontinuing long‐acting injectable depot naltrexone as a relapse prevention pharmacotherapy in opioid use disorder at three months following initiation.
**Table S10:** Univariable meta‐regression of individual and study‐level factors associated with the proportion of individuals discontinuing long‐acting injectable depot naltrexone as a relapse prevention pharmacotherapy in opioid use disorder at six months following initiation.
**Table S11:** Preferred Reporting Items for Systematic Reviews and Meta‐Analyses (PRISMA) Checklist.
**Table S12:** Meta‐analysis of Observational Studies in Epidemiology (MOOSE) checklist.

## Data Availability

This work represents a systematic review of published research. All data is publicly available.
