# Supplementary material for: Factors associated with the percentage of individuals who initiate and discontinue naltrexone as a relapse prevention pharmacotherapy in opioid use disorder: A systematic review, meta‐analysis and meta‐regression
Source: Addiction. 2026 Jun 12;121(10):2620–32. doi: 10.1111/add.70502 (PMC13578835; doi:10.1111/add.70502)
Supplement: Supplementary file 1 [file ADD-121-2620-s001.docx]

**Online Supplementary Material**

**Contents**

**Figures**

Figure S1: Search strategies

Figure S2: Extracted variable definitions

Figure S3: Adapted Newcastle-Ottawa checklist

Figure S4: GRADE certainty assessment

Figure S5: PRISMA Flow Diagram

Figure S6: Forest plot of the pooled proportion of individuals who initiated naltrexone as a relapse prevention pharmacotherapy for opioid use disorder among those who were eligible to receive it (i.e., people who have completed opioid withdrawal and wish to maintain abstinence from extra-medical opioid use) in the subgroups of studies conducted in i) routine clinical care and ii) investigational settings

Figure S7: Forest plots of the pooled proportion of individuals who discontinued naltrexone as a relapse prevention pharmacotherapy for opioid use disorder at a) one-month, b) three-months and c) six-months following initiation in the subgroups of studies conducted in i) routine clinical care and ii) investigational settings

Figure S8: The proportion of individuals initiating and discontinuing naltrexone in the ten studies containing data on both initiation and discontinuation within the same sample

Figure S9: Funnel plot of estimates of proportion of individuals initiating naltrexone

Figure S10: Funnel plot of estimates of proportion of individuals discontinuing naltrexone at one month following initiation

Figure S11: Funnel plot of estimates of proportion of individuals discontinuing naltrexone at three months following initiation

Figure S12: Funnel plot of estimates of proportion of individuals discontinuing naltrexone at six months following initiation

**Tables**

Table S1: Excluded Studies

Table S2: Description of included studies

Table S3: GRADE clinical evidence profile for estimates of the proportion of individuals initiating naltrexone

Table S4: GRADE clinical evidence profile for pooled estimates of the proportion of individuals discontinuing naltrexone at one month following initiation

Table S5: GRADE clinical evidence profile for pooled estimates of the proportion of individuals discontinuing naltrexone at three months following initiation

Table S6: GRADE clinical evidence profile for pooled estimates of the proportion of individuals discontinuing naltrexone at six months following initiation

Table S7: Univariable meta-regression of individual and study-level factors associated with the proportion of individuals discontinuing oral naltrexone as a relapse prevention pharmacotherapy in opioid use disorder at three months following initiation

Table S8: Univariable meta-regression of individual and study-level factors associated with the proportion of individuals discontinuing oral naltrexone as a relapse prevention pharmacotherapy in opioid use disorder at six months following initiation

Table S9: Univariable meta-regression of individual and study-level factors associated with the proportion of individuals discontinuing long-acting injectable depot naltrexone as a relapse prevention pharmacotherapy in opioid use disorder at three months following initiation

Table S10: Univariable meta-regression of individual and study-level factors associated with the proportion of individuals discontinuing long-acting injectable depot naltrexone as a relapse prevention pharmacotherapy in opioid use disorder at six months following initiation

Table S11: Preferred Reporting Items for Systematic Reviews and Meta-Analyses (PRISMA) Checklist

Table S12: Meta-analysis of Observational Studies in Epidemiology (MOOSE) checklist

**Figure S1: Search strategies**

Search strategy for Medline, Embase and PsychINFO using the Ovid interface

1. (naltrexone or vivitrol or revia or depade or depotrex or prodetoxone).mp
2. (opioid* or opiate*).mp
3. (detox* or withdraw* or initiat* or start* or cessation or stop* or discontinu* or retain* or retention*).mp
4. 1 and 2 and 3

Search strategy for the Cochrane Central Register of Controlled Trials (CENTRAL)

#1 (naltrexone or vivitrol or revia or depade or depotrex or prodetoxone):ti,ab,kw

#2 (opioid* or opiate*):ti,ab,kw

#3 (detox* or withdraw* or initiat* or start* or cessation or stop* or discontinu* or retain* or retention*):ti,ab,kw

#4 (#1 and #2 and #3)

**Figure S2: Extracted variable definitions**

**Study ID (String)**

Free text study name and year (e.g. Wenzel 2024)

**Median year of data collection (Continuous)**

The median year of data collection reported by the study (e.g., 2004)

**Country of data collection (Categorical)**

The country of data collection reported by the study e.g.,

1: USA

2: UK

3: Australia

etc.

**Study design (Categorical)**

Study design

1: Clinical trial

2: Prospective cohort

3: Retrospective cohort

**Prospective investigational study setting (Binary)**

1: Prospective investigational study (i.e., studies conducted in a research context according to a study protocol)

2: Studies of records taken from routine clinical care

**Study setting (Categorical)**

The setting in which study takes place as reported

1: Outpatient Drug Service

2: Specialist Inpatient Unit

3: Prison/Jail

4: Other Outpatient Service

5: Community

**Non-selective sample (Binary)**

Did the study report the recruitment a sample of non-selective individuals with opioid use disorder? (i.e., the study did not report specifically recruiting individuals with a particular sociodemographic or clinical feature (e.g., people who are HIV positive, people within the criminal justice system)

1: Yes - Reported as non-selective

2: No - Reported as selective

**Type of detoxification (Categorical)**

What type of detoxification process did individuals reportedly undergo?

1: Inpatient

2: Outpatient (i.e., a managed taper of opioid agonist therapy conduced as an outpatient)

3: Rapid (i.e., conducted as an inpatient under anaesthesia or sedation)

4. Self

5. Multiple types of detoxification

**Type of opioid used prior to opioid withdrawal (Binary)**

What type/s of opioid were used by the majority of participants prior to undergoing opioid withdrawal and the commencement of naltrexone?

1. Prescribed Opioid Agonist Therapy (OAT) (e.g., methadone or buprenorphine etc.)

2. Extra-medical or illicit use of opioids (e.g., heroin, fentanyl, extra-medical use of prescription opioids etc.)

**Naltrexone formulation (Categorical)**

What was the formulation of naltrexone reportedly examined?

1: Oral

2: Long-acting injectable depot

3: Implantable

4: Multiple formulations

**Dose: (String)**

What dose of naltrexone does the study report? (e.g., 50 milligrams per day, 380 milligrams per month)

**Supervision (Binary)**

Does the study report that naltrexone was administered under supervision (e.g., by a healthcare professional or family member*)

1: Yes - Reported as supervised

2: No - Not reported as supervised

*All administrations of long-acting injectable depot and implants are, by definition, supervised by a healthcare professional

**Naltrexone the only pharmacotherapy offered at the point of relapse prevention (Binary)**

Was naltrexone reported as the only pharmacotherapy offered following opioid withdrawal?

1: Yes - Naltrexone reported as the only pharmacotherapy offered following opioid withdrawal

2: No – Naltrexone was not reported as the only pharmacotherapy offered following opioid withdrawal (e.g., methadone or buprenorphine offered)

**Psychosocial therapy (Binary)**

Was any form of psychosocial therapy reported as being available to individuals in combination with naltrexone?

1: Yes - Psychosocial therapy was reported as available to individuals alongside naltrexone

2: No - Psychosocial therapy was not reported as available to individuals alongside naltrexone

**Bespoke psychosocial therapy designed to improve naltrexone adherence (Binary)**

Was a form of psychosocial therapy specifically designed to improve adherence to naltrexone reported as being available to individuals in combination with naltrexone?

1: Yes - Bespoke psychosocial therapy designed to improve adherence was reported as available to individuals alongside naltrexone

2: No - Bespoke psychosocial therapy designed to improve adherence was not reported as available to individuals alongside naltrexone

**Motivation (Binary)**

Did the study report formal assessment of the level of motivation of individuals to maintain abstinence from extra-medical opioid use?

1: Yes – Reported formal assessment of motivation

2: No – Did not report formal assessment of motivation

**High motivation (Binary)**

If the study reported a formal assessment of the level of motivation of individuals to maintain abstinence or comply with naltrexone pharmacotherapy, did it report the majority of individuals were highly motivated to maintain abstinence from extra-medical opioid use?

1: Yes - Reported majority of individuals were highly motivated

2: No - Did not report that the majority of individuals were highly motivated

**Mean age of individuals (Continuous)**

The mean age of individuals in the sample (e.g., 29.4 years)

**Percentage female (Continuous)**

The percentage of female individuals in the sample (e.g., 29%)

**Percentage employed (Continuous)**

The percentage of employed individuals in the sample (e.g., 49%)

**Figure S3: Adapted Newcastle-Ottawa Assessment Scale**

**Selection:** (Maximum four points)

1) Representativeness of the sample

a) Truly representative of the average in the target population (all subjects or random sampling) (2)

b) Somewhat representative of the average in the target population (non-random/purposive sampling) (1)

c) Selected group of individuals (i.e., specific demographic or clinical population) (0)

d) No description of the sampling strategy (0)

2) Non-participants

a) Comparability between included individuals and eligible non included individuals is established, and the participation rate is satisfactory (>60%) (1)

b) The participation rate is unsatisfactory, or the comparability between included individuals and non-included individuals is unsatisfactory (0)

c) No description of the response rate or the characteristics of the included and non-included individuals (0)

3) Sample size

a) Justified and satisfactory (>50) (1)

b) Not justified or satisfactory (≤ 49)

**Comparability:** (Maximum two points)

1) The individuals are comparable across studies based on diagnosis, based on the study design or analysis

a) Comparable (2)

b) Non-comparable (0)

**Outcome:** (Maximum three points)

1) Assessment of the proportion of individuals initiating or discontinuing naltrexone:

a) Clear description of number of individuals eligible, initiating or discontinuing naltrexone at each time point (2)

b) Some description of number of individuals eligible, initiating or discontinuing naltrexone at each time point (1)

c) No description (0)

2) Proportion measurement

a) Reports results enabling calculation of proration with numerator and denominator (1)

b) Reports results enabling calculation of proration with numerator and denominator (1)

This scale has been adapted from the Newcastle-Ottawa Quality Assessment Scale. The maximum score is nine.

**Figure S4: GRADE Certainty Assessment**

Risk of Bias: Risk of bias was assessed using a quality assessment tool adapted from the Newcastle-Ottawa Scale. The maximum available score was nine, higher scores indicating higher quality. There was deemed to be minimal risk of bias, and thus the overall certainty rating was not downgraded, if the pooled average total quality score was ≥ 7. The overall certainty rating was downgraded by one level (e.g. a reduction from *low* to *very low*) if the weighted average total quality score per estimate was 4-6, and the overall certainty rating was downgraded by two levels if the weighted average total quality score per estimate was ≤ 3.

Inconsistency: Inconsistency refers to the level of heterogeneity between estimates and was assessed using the I^2^ statistic. The overall certainty rating was not downgraded if I^2^ was < 50%, was downgraded by one level if I^2^ was ≥ 50% and < 75%, and by two levels if I^2^ was ≥ 75%.

Indirectness: Indirectness refers to the extent to which the study population or outcome differ from those specified in the inclusion criteria. As all studies fulfilled the inclusion criteria no estimate was downgraded due to indirectness.

Imprecision: Imprecision refers to the extent to which we are uncertain about the estimates and based on examination of the confidence interval (CI). As no guidelines exist as to what represents an appropriate precision of proportion estimates, the following criteria were developed: the overall certainty rating was not downgraded if the precision (i.e. half the CI) was < 5%, was downgraded by one level if the precision was ≥ 5 % and < 10%, and by two levels if the precision was ≥ 10%.

Other considerations: GRADE also considers other potential sources of bias, and publication bias was assessed within this domain. Consistent with the Cochrane handbook, (1) in cases where ≥ 10 studies were included in the meta-analysis funnel plots were produced. These were visually inspected to explore the possibility of preferential publication of small studies reporting inflated prevalence estimates, and in addition we performed Egger’s test for small study effects. (2) Where evidence was suggestive of publication bias the overall certainty rating was downgraded by one level.

**Figure S5: PRISMA flow diagram**

Records identified through database searching
(n = 5150)

## Screening

## Included

## Eligibility

## Identification

Additional records identified through other sources
(n = 104)

Records after duplicates removed
(n = 3790)

Records screened
(n = 3790)

Records excluded
(n = 3155)

Full-text articles assessed for eligibility
(n = 635)

Full-text articles excluded, with reasons
(n = 538)

Studies included
(n = 97)

Number of proportion estimates included in quantitative synthesis
(n = 22 Initiation,

n = 95 Discontinuation)

**Figure S6:** **Forest plot of the pooled proportion of individuals who initiated naltrexone as a relapse prevention pharmacotherapy for opioid use disorder among those who were eligible to receive it (i.e., people who have completed opioid withdrawal and wish to maintain abstinence from extra-medical opioid use) in the subgroups of studies conducted in i) routine clinical care and ii) investigational settings**

i)

ii)

**Figure S7: Forest plots of the pooled proportion of individuals who discontinued naltrexone as a relapse prevention pharmacotherapy for opioid use disorder at a) one-month, b) three-months and c) six-months following initiation in the subgroups of studies conducted in i) routine clinical care and ii) investigational settings**

a) i)

a) ii)

b) i)

b) ii)

c) i)

c) ii)

**Figure S8: The proportion of individuals initiating and discontinuing naltrexone in the ten studies containing data on both initiation and discontinuation within the same sample**

^O = Oral; D = Long-acting injectable depot^

**Figure S9: Funnel plot of the estimates of the proportion of individuals initiating naltrexone**

Eggers test for small study effects: *p*<0.001

**Figure S10: Funnel plot of estimates of proportion of individuals discontinuing naltrexone at one month following initiation**

Eggers test for small study effects: *p*<0.001

**Figure S11: Funnel plot of estimates of proportion of individuals discontinuing naltrexone at three months following initiation**

Eggers test for small study effects: *p*=0.184

**Figure S12: Funnel plot of estimates of proportion of individuals discontinuing naltrexone at six months following initiation**

Eggers test for small study effects: *p*=0.018

**Tables**

**Table S1: Excluded studies**

| 1. Abbott PJ. A review of the community reinforcement approach in the treatment of opioid dependence. Journal of Psychoactive Drugs. 2009;41(4):379-85. | Exclude: Review article all references checked for inclusion |
| --- | --- |
| 2. Adi Y, Juarez-Garcia A, Wang D, Jowett S, Frew E, Day E, et al. Oral naltrexone as a treatment for relapse prevention in formerly opioid-dependent drug users: a systematic review and economic evaluation. Health Technol Assess. 2007;11(6):iii-iv, 1-85. | Exclude: Review article all references checked for additional citations |
| 3. Ahamad K, Milloy MJ, Nguyen P, Uhlmann S, Johnson C, Korthuis TP, et al. Factors associated with willingness to take extended release naltrexone among injection drug users. Addict Sci Clin Pract. 2015;10:12. | Exclude: Cross sectional survey of willingness to take naltrexone; No empirical data of calculable numbers initiating or discontinuing naltrexone reported. |
| 4. Ahmadi J, Ahmadi K, Ohaeri J. Controlled, randomized trial in maintenance treatment of intravenous buprenorphine dependence with naltrexone, methadone or buprenorphine: a novel study. European journal of clinical investigation. 2003;33(9):824-9. | Exclude: Reports “treatment retention” not discontinuation of naltrexone |
| 5. AIa G, Krupitskiĭ E, Zvartau E. Pharmacotherapy in heroin addiction: pharmacological approaches to remission stabilization and recurrence prevention. Vestnik Rossiiskoi Akademii Meditsinskikh Nauk. 2003(10):54-6. | Exclude: RCT does not report discontinuation rates |
| 6. Aklin WM, Severtson SG, Umbricht A, Fingerhood M, Bigelow GE, Lejuez CW, et al. Risk-taking propensity as a predictor of induction onto naltrexone treatment for opioid dependence. Journal of Clinical Psychiatry. 2012;73(8):e1056-61. | Exclude: Assess success of naltrexone induction among a cohort of people detoxified in order to start naltrexone, not the number of people choosing to initiate naltrexone in an eligible sample. |
| 7. Al-Eitan LN, Jaradat SA, Su SY, Tay GK, Hulse GK. Mu opioid receptor (OPRM1) as a predictor of treatment outcome in opiate-dependent individuals of Arab descent. Pharmacogenomics and Personalized Medicine. 2012;5(1):99-111. | Exclude: Does not report calculable rates of naltrexone discontinuation |
| 8. Albanese AP, Gevirtz C, Oppenheim B, Field JM, Abels I, Eustace JC. Outcome and six month follow up of patients after Ultra Rapid Opiate Detoxification (UROD). Journal of Addictive Diseases. 2000;19(2):11-28. | Exclude: Paper assessing withdrawal strategies; All patients aimed to be inducted onto naltrexone following detoxification; Nil other treatment options available and no prevalence estimate of number of patients opting to initiate naltrexone calculable. Nil discontinuation data reported. |
| 9. Aletraris L, Bond Edmond M, Roman PM. Adoption of injectable naltrexone in U.S. substance use disorder treatment programs. Journal of Studies on Alcohol & Drugs. 2015;76(1):143-51. | Exclude: Cross sectional survey of services to implement XR-NTX; No empirical data of calculable numbers initiating or discontinuing naltrexone reported. |
| 10. Ali R, Thomas P, White J, McGregor C, Danz C, Gowing L, et al. Antagonist-precipitated heroin withdrawal under anaesthetic prior to maintenance naltrexone treatment: Determinants of withdrawal severity. Drug and Alcohol Review. 2003;22(4):425-31. | Exclude: Paper assessing withdrawal; All patients aimed to be inducted onto naltrexone following detoxification; No prevalence estimate of number of patients opting to initiate naltrexone calculable. Nil discontinuation of naltrexone data reported. |
| 11. Allen AA, Chen DT, Bonnie RJ, Ko TM, Suratt CE, Lee JD, et al. Assessing informed consent in an opioid relapse prevention study with adults under current or recent criminal justice supervision. Journal of Substance Abuse Treatment. 2017;81:66-72. | Exclude: Results of informed consent survey on people using XR-NTX |
| 12. Amato L, Davoli M, Ferri M, Gowing L, Perucci CA. Effectiveness of interventions on opiate withdrawal treatment: An overview of systematic reviews. Drug and Alcohol Dependence. 2004;73(3):219-26. | Exclude: Review of review of withdrawal regimens; All references checked for inclusion |
| 13. Anderson BJ, Bailey GL, Flori JN, Risi MM, Conti MT, Stein MD. Starting injectable naltrexone during opioid detoxification: Linkage to primary care. Journal of Addiction Medicine. 2016;10(3):E8. | Exclude: Conference abstract of included study Stein 2016 with duplicate data |
| 14. Andraka-Christou B, Atkins D. Beliefs about medications for opioid use disorder among Florida criminal problem-solving court & dependency court staff. Am J Drug Alcohol Abuse. 2020;46(6):749-60. | Exclude: Review article all references checked for inclusion |
| 15. Andraka-Christou B, Capone MJ. A qualitative study comparing physician-reported barriers to treating addiction using buprenorphine and extended-release naltrexone in U.S. office-based practices. International Journal of Drug Policy. 2018;54:9-17. | Exclude: Qualitative study of barriers to XR-Naltrexone |
| 16. Andrew Tompkins D, Nunes E, Nangia N, Akerman S. Baseline characteristics predictive of transition onto extended-release naltrexone. Journal of Addiction Medicine. 2018;12(3):E3-E4. | Exclude: Conference abstract assessing predictors of successful induction to XR-NTX not rate of those opting to initiate naltrexone |
| 17. Anonymous. Baseline characteristics of patients predicting suitability for rapid naltrexone induction. American Journal on Addictions. 2015;24(3). | Exclude: Reports factors of success to initiation following rapid withdrawal not number opting to initiate naltrexone. References checked for includable studies |
| 18. Arechiga G, Bonilla J, Mendoza I, Correa J, Ruiz Y. Naltrexone implants improve therapeutic adherence with multiple substance use disorder. Journal of Addiction Medicine. 2019;13(3):E13. | Exclude: Conference Abstract: Unclear numbers in each arm trial of the trial; No rates of discontinuation calculable |
| 19. Arndt I, McLellan AT, O'Brien CP. Abstinence treatments for opiate addicts: Therapeutic community or naltrexone? NIDA Research Monograph Series. 1984;NO. 49:275-81. | Exclude: Comparative study with naltrexone arm: No empirical data reported on the prevalence of or the factors associated with naltrexone initiation/discontinuation |
| 20. Arnold-Reed DE, O'Neil P, Holman CD, Bulsara MK, Rodiguez C, Gawthorne G, et al. A comparison of mental health hospital admissions in a cohort of heroin users prior to and after rapid opiate detoxification and oral naltrexone maintenance. Am J Drug Alcohol Abuse. 2007;33(5):655-64. | Exclude: Does not report empirical naltrexone initiation or discontinuation data |
| 21. ARNOLD‐REED D, Hulse G, Hansson R, Murray S, O'NEIL G, Basso M, et al. Blood morphine levels in naltrexone‐exposed compared to non‐naltrexone‐exposed fatal heroin overdoses. Addiction biology. 2003;8(3):343-50. | Exclude: Reports mortality in naltrexone vs non-naltrexone groups; Nil initiation or discontinuation data reported |
| 22. Atluru S, Bruehlman AK, Vaughn P, Schauberger CW, Smid MC. Naltrexone Compared With Buprenorphine or Methadone in Pregnancy: A Systematic Review. Obstet Gynecol. 2024;143(3):403-10. | Exclude: Systematic review all articles checked for inclusion |
| 23. Badaras R, Jovaisa T, Judickas S, Ivaskevicius J. Changes in potassium concentration during opioid antagonist induction: Comparison of two randomized clinical trials. Journal of Addiction Medicine. 2016;10(4):244-7. | Exclude: Examination of electrolyte disturbance during opioid withdrawal |
| 24. Badaras R, Jovaisa T, Lapinskiene I, Ivaskevicius J. Dose Escalation of Naltrexone to Reduce Stress Responses Associated With Opioid Antagonist Induction: A Double-blind Randomized Trial. Journal of Addiction Medicine. 2020;14(3):253-60. | Exclude: Clinical trial exploring different dosage regimens for naltrexone induction; No initiation or discontinuation data reported |
| 25. Bagley SM, Larochelle MR, Xuan Z, Wang N, Patel A, Bernson D, et al. Characteristics and receipt of medication treatment among young adults who experience a nonfatal opioid-related overdose. Annals of Emergency Medicine. 2020;75(1):29-38. | Exclude: Reports Number of young adults in receipt of medication for opioid use disorder among a claims database; Unclear in denominator whom is eligible for naltrexone as a relapse prevention medication. |
| 26. Bahji A, Carlone D, Altomare J. Acceptability and efficacy of naltrexone for criminal justice-involved individuals with opioid use disorder: a systematic review and meta-analysis. Addiction. 2020;115(8):1413-25. | Exclude: Systematic review all references checked for inclusion |
| 27. Bailey A, Senthilkumar R, Evans EA. Correlates and Patterns in Use of Medications to Treat Opioid Use Disorder in Jail. Journal of Addiction Medicine. 2023;17(5):568-73. | Exclude: Reports number of people on medications for opioid use disorder in jail population; unclear of number eligible for naltrexone; nil data reported to enable calculable rate of initiation or discontinuation |
| 28. Ball SA, Nich C, Rounsaville BJ, Eagan D, Carroll KM. Millon Clinical Multiaxial Inventory-III subtypes of opioid dependence: validity and matching to behavioral therapies. J Consult Clin Psychol. 2004;72(4):698-711. | Exclude: Does not report empirical initiation or discontinuation data |
| 29. Balter DR, Puglisi LB, Dziura J, Fiellin DA, Howell BA. Buprenorphine-naloxone vs. extended-release naltrexone for opioid use disorder in individuals with and without criminal legal involvement: A secondary analysis of the X:BOT randomized controlled trial. Journal of Substance Use and Addiction Treatment. 2024;164:209438. | Exclude: Secondary analysis of screened clinical trial reporting on criminal legal involvement outcomes |
| 30. Barbieri VA, Mishlen K, Sullivan M, Bisaga A. Baseline predictors of outpatient induction onto extended-release naltrexone. Drug and Alcohol Dependence. 2017;Conference: 2016 Annual Meeting of the College on Problems of Drug Dependence, CPDD 2016. Palm Springs, CA United States. 171:e15. | Exclude: Conference abstract of success of induction onto XR-NTX not assessing rate of opting into initiation of naltrexone |
| 31. Barnett ML, Meara E, Lewinson T, Hardy B, Chyn D, Onsando M, et al. Racial Inequality in Receipt of Medications for Opioid Use Disorder. New England Journal of Medicine. 2023;388(19):1779-89. | Exclude: Reports number of people with OUD received a prescription for naltrexone; Any receipt in 180 days; Does not report calculable rate of initiation or discontinuation. |
| 32. Bartter T, Gooberman LL. Rapid opiate detoxification. Am J Drug Alcohol Abuse. 1996;22(4):489-95. | Exclude: Paper assessing withdrawal regimens; All patients aimed to be inducted onto naltrexone following detoxification; Nil other treatment options available and no prevalence estimate of number of patients opting to initiate naltrexone calculable. Nil discontinuation data reported. |
| 33. Baser O, Chalk M, Fiellin DA, Gastfriend DR. Cost and utilization outcomes of opioid-dependence treatments. American Journal of Managed Care. 2011;17 Suppl 8:S235-48. | Exclude: Reports Medication Possession Ratios >0.8 as a measure of compliance but no time of discontinuation of calculable rate of discontinuation at any time point. |
| 34. Bassiony MM, Abdelfattah NR, Elshabrawy A, Adly MM. A comparative study of the efficacy of venlafaxine and naltrexone for relapse prevention in patients with opioid use disorder attributed to tramadol. Int Clin Psychopharmacol. 2024;39(6):341-9. | Exclude: Reports treatment retention not discontinuation of naltrexone rates; No data to enable calculable rate of discontinuation presented |
| 35. Bateman J, Gilvarry E, Tziggili M, Crome IB, Mirza K, McArdle P. Psychopharmacological treatment of young people with substance dependence: A survey of prescribing practices in England. Child and Adolescent Mental Health. 2014;19(2):102-9. | Exclude: Cross sectional survey of prescribing practices. |
| 36. Beaini AY, Johnson TS, Langstaff P, Carr MP, Crossfield JN, Sweeney RC. A compressed opiate detoxification regime with naltrexone maintenance: Patient tolerance, risk assessment and abstinence rates. Addiction Biology. 2000;5(4):451-62. | Exclude: Does not report number of participants initiating naltrexone to enable calculation of initiation or discontinuation rate. Reports the percentage of participants who were naltrexone compliant only among those achieving abstinence and also reports data on a random selection of patients “Pathology data on 51 patients 6 months post-detoxification selected at random and divided according to their use of naltrexone maintenance (n = 29 on maintenance, n = 22 stopped maintenance at 2.6 ± 0.5 months)” |
| 37. Beauchamp GA, Laubach LT, Esposito SB, Yazdanyar A, Roth P, Lauber P, et al. Implementation of a Medication for Addiction Treatment (MAT) and Linkage Program by Leveraging Community Partnerships and Medical Toxicology Expertise. J Med Toxicol. 2021;17(2):176-84. | Exclude: Results not stratified by substance use disorder or naltrexone as a medication for opioid use disorder |
| 38. Beauchamp GA, Lauber P, Allen J, Boateng N, Shaak S, Roth P, et al. 309 Overcoming Brick and Mortar: Feasibility of Implementation of a Medication for Addiction Treatment and Linkage to Treatment Program by Leveraging Community Partnerships. Annals of Emergency Medicine. 2019;74(4 Supplement):S121-S2. | Exclude: Conference Abstract: Non opioid use disorder cohort; All substance use disorder not discernible number taking naltrexone for OUD |
| 39. Bell LA, Dir AL, Tu W, Aalsma MC. 86. Characteristics of Youth Receiving Medication Treatment for Opioid Use Disorder. Journal of Adolescent Health. 2021;68(2 Supplement):S46. | Exclude: Conference Abstract; No empirical data reported on the prevalence of or the factors associated with naltrexone initiation/discontinuation. Dispensing data reported and unclear within selected cohort the number eligible for naltrexone. |
| 40. Biondi BE, Vander Wyk B, Schlossberg EF, Shaw A, Springer SA. Factors associated with retention on medications for opioid use disorder among a cohort of adults seeking treatment in the community. Addict Sci Clin Pract. 2022;17(1):15. | Exclude: No naltrexone data |
| 41. Bisaga A, Mannelli P, Sullivan MA, Vosburg SK, Compton P, Woody GE, et al. Antagonists in the medical management of opioid use disorders: Historical and existing treatment strategies. American Journal on Addictions. 2018;27(3):177-87. | Exclude: Review article all references checked for inclusion |
| 42. Bisaga A, Nunes E, Akerman S, Hu MC, Mishlen K, Legedza A, et al. Predictors of xr-ntx initiation in patients with opioid use disorder: Integrated database findings. American Journal on Addictions. 2020;29(3):230-1. | Exclude: Conference abstract reporting induction data from five screened clinical trials |
| 43. Bisaga A, Sullivan MA, Cheng WY, Carpenter KM, Mariani JJ, Levin FR, et al. A placebo controlled trial of memantine as an adjunct to oral naltrexone for opioid dependence. Drug Alcohol Depend. 2011;119(1-2):e23-9. | Exclude: Reports drop out of trial not discontinuation and reports compliance for studied memantine not naltrexone |
| 44. Bisaga A, Sullivan MA, Glass A, Mishlen K, Carpenter KM, Mariani JJ, et al. A placebo-controlled trial of memantine as an adjunct to injectable extended-release naltrexone for opioid dependence. Journal of Substance Abuse Treatment. 2014;46(5):546-52. | Exclude: Reports retention in treatment. No empirical data reported to enable calculation of rate of naltrexone initiation or discontinuation. |
| 45. Bisaga A, Sullivan MA, Glass A, Mishlen K, Pavlicova M, Haney M, et al. The effects of dronabinol during detoxification and the initiation of treatment with extended release naltrexone. Drug Alcohol Depend. 2015;154:38-45. | Exclude: Reports retention in treatment and compliance rates for dronabinol not naltrexone. No empirical data reported to enable calculation of rate of naltrexone initiation or discontinuation. |
| 46. Blansfield HN. Methadone vs naltrexone. Connecticut Medicine. 1997;61(1):53. | Exclude: No empirical data reported on the prevalence of or the factors associated with naltrexone initiation/discontinuation |
| 47. Blokhina E, Krupitsky E, Bushara N, Yaroslavtseva T, Tiurina A, Palatkin V, et al. Implantable and oral naltrexone for preventing relapse in opiate addicts: A psychometric evaluation. Drug and Alcohol Dependence. 2015;Conference: 2014 Annual Meeting of the College on Problems of Drug Dependence. San Juan Puerto Rico. 146:e224. | Exclude: Conference abstract of screened clinical trial reporting on psychiatric symptoms |
| 48. Blokhina E, Krupitsky E, Kibitov A, Verbitskaya E, Kosten T, Nielsen DA, et al. The role of genetic polymorphisms on patient response to opioid use disorder therapy with naltrexone and guanfacine. Drug and Alcohol Dependence. 2017;Conference: 2016 Annual Meeting of the College on Problems of Drug Dependence, CPDD 2016. Palm Springs, CA United States. 171:e21. | Exclude: Conference abstract of screened clinical trial. Nil discontinuation or initiation outcomes reported |
| 49. Bochud Tornay C, Favrat B, Monnat M, Daeppen JB, Schnyder C, Bertschy G, et al. Ultra-rapid opiate detoxification using deep sedation and prior oral buprenorphine preparation: long-term results. Drug Alcohol Depend. 2003;69(3):283-8. | Exclude: Paper assessing withdrawal; All patients aimed to be inducted onto naltrexone following detoxification; No prevalence estimate of number of patients opting to initiate naltrexone calculable. Discontinuation of naltrexone data reported but discontinuation rate not calculable. |
| 50. Bovill JG. Opioid detoxification under anaesthesia. European Journal of Anaesthesiology. 2000;17(11):657-61. | Exclude: Editorial all references checked |
| 51. Bradford A, Hurley F, Golondzowski O, Dorrier C. Interim report on clinic intake and safety data collected from 17 NIDA-funded naltrexone studies. NIDA Res Monogr. 1976;9:163-71. | Exclude: Reports aggregate data for seventeen NIDA studies involving naltrexone - where possible individual studies have been checked for inclusion. Initiation data is reported per study and not by arm and includes data for placebo induction. Figure reporting the cumulative retention on naltrexone and other arms does not provide denominator for number of patients receiving naltrexone, unable to calculate discontinuation rate from presented data. |
| 52. Bradford HA, Kaim SC, Ling W. A summary of the phase II clinical studies of the narcotic antagonist, naltrexone.  Drug Dependence and Alcoholism: Volume 1 Biomedical Issues: Springer; 1981. p. 981-91. | Exclude: Clinical trial naltrexone vs placebo; nil naltrexone discontinuation data presented or calculable. |
| 53. Brahen LS, Capone T, Wiechert V, Desiderio D. Naltrexone and cyclazocine: a controlled treatment study. Archives of General Psychiatry. 1977;34(10):1181-4. | Exclude: Clinical trial; No calculable discontinuation naltrexone data reported |
| 54. Brahen LS, Henderson RK, Capone T, Kordal N. Naltrexone treatment in a jail work-release program. Journal of Clinical Psychiatry. 1984;45(9 II):49-52. | Exclude: No empirical data reported on number of patients initiating naltrexone or the number eligible to receive it nor the number discontinuing naltrexone |
| 55. Breen CL, Harris SJ, Lintzeris N, Mattick RP, Hawken L, Bell J, et al. Cessation of methadone maintenance treatment using buprenorphine: transfer from methadone to buprenorphine and subsequent buprenorphine reductions. Drug and alcohol dependence. 2003;71(1):49-55. | Exclude: Does not report the number of patients initiating naltrexone post detoxification; Only reports the number of patients taking naltrexone at one month follow up. No data reported to enable calculable rate of initiation or discontinuation |
| 56. Brewer C. Opiate withdrawal: inpatient versus outpatient programmes. Br Med J (Clin Res Ed). 1986;293(6543):391. | Exclude: Letter; No empirical data reported on the prevalence of or the factors associated with naltrexone initiation/discontinuation |
| 57. Brewer C. Ultra-rapid, antagonist-precipitated opiate detoxification under general anaesthesia or sedation. Addiction Biology. 1997;2(3):291-302. | Exclude: Review article of rapid detoxification; Discusses patients aimed to be inducted onto naltrexone following detoxication; Nil other treatment options available and no prevalence estimate of number of patients opting to initiate naltrexone calculable. Nil discontinuation data reported. |
| 58. Brewer C. Optimising patient care using naltrexone for opioid use disorders.  Handbook on optimizing patient care in psychiatry. New York, NY: Routledge; US; 2023. p. 300-16. | Exclude: Review chapter all references checked for inclusion |
| 59. Brewer C, Krupitsky E. Antagonists for the treatment of opioid dependence.  Comprehensive addictive behaviors and disorders, Vol 3: Interventions for addiction. San Diego, CA: Elsevier Academic Press; US; 2013. p. 427-38. | Exclude: Book; Review chapter all references checked for potential inclusion |
| 60. Brewer C, Rezae H, Bailey C. Opioid withdrawal and naltrexone induction in 48-72 hours with minimal drop-out, using a modification of the naltrexone-clonidine technique. British Journal of Psychiatry. 1988;153:340-3. | Exclude: Paper assessing induction onto naltrexone; All patients aimed to be inducted onto naltrexone following detoxication; Nil other treatment options available and no prevalence estimate of number of patients opting to initiate naltrexone calculable. Nil discontinuation data reported. |
| 61. Brewer C, Streel E. Recent developments in naltrexone implants and depot injections for opiate abuse: the new kid on the block is approaching adulthood. Adicciones. 2010;22(4):285-91. | Exclude: Review article all references checked for inclusion |
| 62. Bromberg DJ, Viera A. Mortality Risk Following Naltrexone Discontinuation: Systematic Review and Meta-Analysis. Journal of Addiction Medicine. 2020;14(6):e404-e5. | Exclude: Conference Abstract; No empirical data in abstract reported on the prevalence of or the factors associated with naltrexone initiation/discontinuation |
| 63. Brooks AC, Comer SD, Sullivan MA, Bisaga A, Carpenter KM, Raby WM, et al. Long-acting injectable versus oral naltrexone maintenance therapy with psychosocial intervention for heroin dependence: a quasi-experiment. Journal of Clinical Psychiatry. 2010;71(10):1371. | Exclude: Reports retention in treatment and clinic absence. Does not report data to enable calculation of rate of naltrexone initiation or discontinuation |
| 64. Bunting AM, Krawczyk N, Choo TH, Pavlicova M, McNeely J, Tofighi B, et al. Polysubstance use before and during treatment with medication for opioid use disorder: Prevalence and association with treatment outcomes. Journal of Substance Abuse Treatment. 2022;143:108830. | Exclude: Secondary analysis of screened clinical trial reporting polysubstance outcomes |
| 65. Buntwal N, Bearn J, Gossop M, Strang J. Naltrexone and lofexidine combination treatment compared with conventional lofexidine treatment for in-patient opiate detoxification. Drug and Alcohol Dependence. 2000;59(2):183-8. | Exclude: Paper assessing withdrawal; All patients aimed to be inducted onto naltrexone following detoxification; No prevalence estimate of number of patients opting to initiate naltrexone calculable. Nil discontinuation of naltrexone data reported. |
| 66. Burnett T, Miller S, Burris J, Blaiklock E, Harlow C. Pharmacists' impact on adherence of addiction treatment with extended-release naltrexone: A retrospective review. Journal of the American Pharmacists Association. 2018;58(3):e175. | Exclude: Conference abstract of design and methods of community study |
| 67. Busch AB, Greenfield SF, Reif S, Normand SLT, Huskamp HA. Outpatient care for opioid use disorder among the commercially insured: Use of medication and psychosocial treatment. Journal of Substance Abuse Treatment. 2020;115(no pagination). | Exclude: Reports number of prescriptions of medications for opioid use disorder, no initiation or discontinuation data reported. |
| 68. Callahan EJ, Rawson RA, McCleave B, Arias R, Glazer M, Liberman RP. The treatment of heroin addiction: Naltrexone alone and with behavior therapy. International Journal of the Addictions. 1980;15(6):795-807. | Exclude: Reports average duration of staying on naltrexone per group not calculable numbers discontinuing from treatment. |
| 69. Camarasa X, Khazaal Y, Besson J, Zullino DF. Naltrexone-assisted rapid methadone discontinuation: a pilot study. European Addiction Research. 2007;13(1):20-4. | Exclude: Assess withdrawal using naltrexone; No initiation of cohort onto naltrexone post detoxification |
| 70. Camenga DR, Colon-Rivera HA, Muvvala SB. Medications for Maintenance Treatment of Opioid Use Disorder in Adolescents: A Narrative Review and Assessment of Clinical Benefits and Potential Risks. Journal of Studies on Alcohol & Drugs. 2019;80(4):393-402. | Exclude: Review article all references checked for inclusion |
| 71. Cantor J, Griffin BA, Levitan B, Mendon-Plasek SJ, Stein BD, Hunter SB, et al. Availability of Medications for Opioid Use Disorder in Community Mental Health Facilities. JAMA Network Open. 2024;7(6):e2417545. | Exclude: Cross sectional survey of treatment providers |
| 72. Capote HA, Zummo JA, Memisoglu A, Rainka M, Silverman BL. Substance use and employment outcomes among patients initiating extended-release naltrexone (XR-NTX): Real-world data from the XR-NTX opioid dependence registry. Value in Health. 2014;17(3):A221. | Exclude: Conference abstract with duplicate data source of Saxon 2018. Excluded to avoid double counting. |
| 73. Carpenter KM, Jiang H, Sullivan MA, Bisaga A, Comer SD, Raby WN, et al. Betting on change: modeling transitional probabilities to guide therapy development for opioid dependence. Psychology of Addictive Behaviors. 2009;23(1):47-55. | Exclude: Reports treatment retention and a compound measure of naltrexone compliance with relapse to opioid use; No empirical data reported to for calculable naltrexone initiation or discontinuation. |
| 74. Carreno JE, Alvarez CE, San Narciso GI, Bascaran MT, Diaz M, Bobes J. Maintenance treatment with depot opioid antagonists in subcutaneous implants: An alternative in the treatment of opioid dependence. Addiction Biology. 2003;8(4):429-38. | Exclude: Reports treatment retention and return to follow up not number of implants received. |
| 75. Carreno JE, Bobes J, Brewer C, Alvarez CE, San Narciso GI, Bascaran MT, et al. 24-Hour opiate detoxification and antagonist induction at home--the 'Asturian method': a report on 1368 procedures. Addiction Biology. 2002;7(2):243-50. | Exclude: Paper assessing withdrawal; All patients aimed to be inducted onto naltrexone following detoxification; No prevalence estimate of number of patients opting to initiate naltrexone calculable. Nil discontinuation of naltrexone data reported |
| 76. Carroll KM, Ball SA, Nich C, O'Connor PG, Eagan DA, Frankforter TL, et al. Targeting behavioral therapies to enhance naltrexone treatment of opioid dependence: efficacy of contingency management and significant other involvement. Archives of General Psychiatry. 2001;58(8):755-61. | Exclude: Reports treatment retention as measured by attendance and mean number of naltrexone doses. Does not report data to enable calculable rate of naltrexone discontinuation |
| 77. Carroll KM, Sinha R, Nich C, Babuscio T, Rounsaville BJ. Contingency management to enhance naltrexone treatment of opioid dependence: A randomized clinical trial of reinforcement magnitude. Experimental and Clinical Psychopharmacology. 2002;10(1):54-63. | Exclude: Clinical trial reports mean number of naltrexone doses and weeks retained in treatment. Do not report calculable number of people discontinuing from naltrexone. |
| 78. Carswell N, Angermaier G, Castaneda C, Delgado F. Management of opioid withdrawal and initiation of medications for opioid use disorder in the hospital setting. Hosp Pract (Minneap). 2022;50(4):251-8. | Exclude: Review article all references checked for inclusion |
| 79. Chambers A, Kleyman S, Snyder M. Effect of a pilot care coordination program on compliance with naltrexone extended release suspension for injection. Journal of the American Pharmacists Association. 2015;55(2):e245. | Exclude: Conference abstract protocol of study |
| 80. Chan KY. The Singapore naltrexone community-based project for heroin addicts compared with drugfree community-based program: The first cohort. Journal of Clinical Forensic Medicine. 1996;3(2):87-8,90-2. | Exclude: Reports retention as measured by being opioid free. No calculable discontinuation data reported. |
| 81. Chand P, Murthy P. Short-term outcome of take-home prescriptions for opioid dependence: A clinic-based study. Journal of Substance Use. 2013;18(2):108-18. | Exclude: Reports relapse to opioid use in a cohort of patients already prescribed either naltrexone or buprenorphine. |
| 82. Chandler RK, Finger MS, Farabee D, Schwartz RP, Condon T, Dunlap LJ, et al. The SOMATICS collaborative: Introduction to a National Institute on Drug Abuse cooperative study of pharmacotherapy for opioid treatment in criminal justice settings. Contemporary Clinical Trials. 2016;48:166-72. | Exclude: Design and rationale paper for clinical trials |
| 83. Chang G, Schein A, Crawford M. Characteristics associated with longacting naltrexone treatment persistence. American Journal on Addictions. 2018;27(4):273-4. | Exclude: Conference abstract of screened included study Chang 2018 |
| 84. Chavez LJ, Bonny AE, Bradley KA, Lapham GT, Cooper J, Miller W, et al. Medication treatment and health care use among adolescents with opioid use disorder in Ohio. Journal of Adolescent Health. 2020;67(1):33-9. | Exclude: Reports Number of people in receipt of medication for opioid use disorder among a claims database; Unclear in denominator whom is eligible for naltrexone as a relapse prevention medication. |
| 85. Chou F, Strom F, Hyatt J. Comparing treatment outcomes in Veterans with opioid use disorder treated with either extended-release naltrexone or buprenorphine at Veterans Affairs Western New York (VAWNY) healthcare system. Journal of Pharmacy Practice. 2016;29(3):295. | Exclude: Conference abstract of protocol paper |
| 86. Church SH, Rothenberg JL, Sullivan MA, Bornstein G, Nunes EV. Concurrent substance use and outcome in combined behavioral and naltrexone therapy for opiate dependence. American Journal of Drug and Alcohol Abuse. 2001;27(3):441-52. | Exclude: Does not report data to calculate number of subjects discontinuing naltrexone |
| 87. Collins ED, Kleber HD, Whittington RA, Heitler NE. Anesthesia-assisted vs buprenorphineor clonidine-assisted heroin detoxification and naltrexone induction: A randomized trial. Jama. 2005;294(8):903-13. | Exclude: Paper assessing withdrawal; All patients aimed to be inducted onto naltrexone following detoxification; No prevalence estimate of number of patients opting to initiate naltrexone calculable. Nil discontinuation of naltrexone data reported |
| 88. Colquhoun RM. Open label trial of naltrexone implants: Measuring blood serum levels of naltrexone. Substance Abuse: Research and Treatment. 2013;7:75-84. | Exclude: Cohort of 66 people started on a naltrexone implant. Unclear number of people discontinuing after 6 months and during implantation “18 who were lost to follow up. It could not be confirmed if they were using at 6 months” |
| 89. Comer SD, Collins ED, Kleber HD, Nuwayser ES, Kerrigan JH, Fischman MW. Depot naltrexone: long-lasting antagonism of the effects of heroin in humans. Psychopharmacology. 2002;159(4):351-60. | Exclude: Tests antagonism of only one dose of depot naltrexone. No data on discontinuation |
| 90. Comer SD, Mannelli P, Alam D, Douaihy A, Nangia N, Akerman SC, et al. Transition of Patients with Opioid Use Disorder from Buprenorphine to Extended-Release Naltrexone: A Randomized Clinical Trial Assessing Two Transition Regimens. American Journal on Addictions. 2020;29(4):313-22. | Exclude: Assess number of people inducted onto XR-NTX in sample that had opted for detoxication/antagonist treatment no denominator calculable for those opting to initiate naltrexone treatment; Nil discontinuation data presented |
| 91. Comer SD, Sullivan MA, Hulse GK. Sustained-release naltrexone: novel treatment for opioid dependence. Expert Opinion on Investigational Drugs. 2007;16(8):1285-94. | Exclude: Review article all references checked for inclusion |
| 92. Comer SD, Sullivan MA, Yu E, Rothenberg JL, Kleber HD, Kampman K, et al. Injectable, sustained-release naltrexone for the treatment of opioid dependence: a randomized, placebo-controlled trial. Archives of General Psychiatry. 2006;63(2):210-8. | Exclude: Clinical trial which reports retention in treatment defined as clinic attendance. No data reported on number receiving second or subsequent XR-NTX injections. |
| 93. Cornish J, Nordeck CD, Langleben DD, Coviello D, Lynch K, Boney T, et al. Six-month depot naltrexone treatment reduces relapse in parolees formerly addicted to opioids. Neuropsychopharmacology. 2012;1):S355. | Exclude: Conference abstract of XR-NTX trial reporting retention |
| 94. Cousins SJ, Crèvecoeur-MacPhail D, Kim T, Rawson RA. The Los Angeles County hub-and-provider network for promoting the sustained use of extended-release naltrexone (XR-NTX) in Los Angeles County (2010–2015). Journal of Substance Abuse Treatment. 2018;85:78-83. | Exclude: Reports all XR-NTX use for all indications including alcohol use disorder; No breakdown of number of injections received by substance diagnosis reported |
| 95. Cousins SJ, Radfar SR, Crèvecoeur-MacPhail D, Ang A, Darfler K, Rawson RA. Predictors of continued use of extended-released naltrexone (XR-NTX) for opioid-dependence: An analysis of heroin and non-heroin opioid users in Los Angeles County. Journal of substance abuse treatment. 2016;63:66-71. | Exclude: Duplicate cohort data on OUD cohort (n=171) included in Cousins 2016 “a demonstration pilot” |
| 96. Coviello DM, Cornish JW, Lynch KG, Alterman AI, O'Brien CP. A randomized trial of oral naltrexone for treating opioid-dependent offenders. American Journal on Addictions. 2010;19(5):422-32. | Exclude: Reports follow up rate and treatment completion not calculable rate of naltrexone discontinuation |
| 97. Crits-Christoph P, Lundy C, Stringer M, Gallop R, Gastfriend DR. Extended-Release Naltrexone for Alcohol and Opioid Problems in Missouri Parolees and Probationers. Journal of Substance Abuse Treatment. 2015;56:54-60. | Exclude: Reports duration of care; No empirical data reported to enable calculation of rate of initiation or discontinuation of naltrexone. |
| 98. Crits-Christoph P, Markell HM, Gibbons MB, Gallop R, Lundy C, Stringer M, et al. A Naturalistic Evaluation of Extended-Release Naltrexone in Clinical Practice in Missouri. Journal of Substance Abuse Treatment. 2016;70:50-7. | Exclude: Does not report data to enable calculation of rate of initiation or discontinuation of naltrexone |
| 99. Cropsey KL, Villalobos GC, St. Clair CL. Pharmacotherapy treatment in substance-dependent correctional populations: A review. Substance Use and Misuse. 2005;40(13-14):1983-99. | Exclude: Review article all references checked |
| 100. Crowley TJ, Wagner JE, Zerbe G, Macdonald M. Naltrexone-induced dysphoria in former opioid addicts. American Journal of Psychiatry. 1985;142(9):1081-4. | Exclude: Trial for dysphoria symptoms in four opioid free men |
| 101. Cucchia AT, Monnat M, Spagnoli J, Ferrero F, Bertschy G. Ultra-rapid opiate detoxification using deep sedation with oral midazolam: Short and long-term results. Drug and Alcohol Dependence. 1998;52(3):243-50. | Exclude: Paper assessing withdrawal and induction onto naltrexone; All patients aimed to be inducted onto naltrexone following detoxification; No prevalence estimate of number of patients opting to initiate naltrexone calculable. Nil discontinuation of naltrexone data reported. |
| 102. Curran S, Savage C. Patient response to naltrexone: issues of acceptance, treatment effects, and frequency of administration. NIDA Res Monogr. 1976;9:67-9. | Exclude: Reports the number of people initiating treatment from the cohort eligible for naltrexone, however the number initiating commences either naltrexone or placebo in the trial, the number eligible is to take part in the trial not to commence naltrexone. Mean number of weeks on treatment presented, no data presented enabling a calculable rate of discontinuation. |
| 103. Cushman Jr P. Narcotic antagonists in the treatment of opiate dependency. Advances in Alcohol and Substance Abuse. 1982;2(1):87-99. | Exclude: Review article all references checked |
| 104. Daniulaityte R, Silverstein SM, Crawford TN, Martins SS, Zule W, Zaragoza AJ, et al. Methamphetamine use and its correlates among individuals with opioid use disorder in a Midwestern US city. Substance Use & Misuse. 2020;55(11):1781-9. | Exclude: Includes people with a lifetime use of XR-NTX. No initiation or discontinuation data reported. |
| 105. Dayal P, Balhara YPS. Profile of female patients seeking in-patient treatment for prescription opioid abuse from a tertiary care drug dependence treatment centre from India. Indian Journal of Medical Research. 2016;143(JANUARY):95-100. | Exclude: Reports a subset of the number that were retained on naltrexone, but not data to allow calculation of rate of initiation or discontinuation |
| 106. Dayal P, Balhara YPS, Mishra A. A retrospective cohort study of predictors of retention and compliance to naltrexone treatment among patients with opioid dependence at tertiary care substance use disorder treatment center. Indian Journal of Psychiatry. 2014;1):S44. | Exclude: Conference abstract reporting predictors of retention on naltrexone. No empirical data of numbers discontinuing treatment calculable from data in abstract. Full paper published in 2016 reviewed for inclusion |
| 107. De Jong CA, Roozen HG, van Rossum LG, Krabbe PF, Kerkhof AJ. High abstinence rates in heroin addicts by a new comprehensive treatment approach. American Journal on Addictions. 2007;16(2):124-30. | Exclude: No empirical data on naltrexone initiation or discontinuation reported |
| 108. Dean AJ, Saunders JB, Jones RT, Young RM, Connor JP, Lawford BR. Does naltrexone treatment lead to depression? Findings from a randomized controlled trial in subjects with opioid dependence. J Psychiatry Neurosci. 2006;31(1):38-45. | Exclude: Reports retention in treatment with number of people attending clinic also splits results by those adherent vs non adherent to naltrexone but no numbers reported at any timepoint allowing calculation of discontinuation rate. |
| 109. Degenhardt L, Larney S, Kimber J, Farrell M, Hall W. Excess mortality among opioid-using patients treated with oral naltrexone in Australia. Drug Alcohol Rev. 2015;34(1):90-6. | Exclude: Reports excess mortality with naltrexone compared to methadone; No empirical data reported to enable calculation of rate of initiation or discontinuation of naltrexone. |
| 110. Dever JA, Hertz MF, Dunlap LJ, Richardson JS, Wolicki SB, Biggers BB, et al. The Medications for Opioid Use Disorder Study: Methods and Initial Outcomes From an 18-Month Study of Patients in Treatment for Opioid Use Disorder. Public Health Rep. 2024;139(4):484-93. | Exclude: Cross sectional study of providers and patients on medication for opioid use disorder; Nil initiation or discontinuation data reported |
| 111. Di Paola A, Lincoln T, Skiest DJ, Desabrais M, Altice FL, Springer SA. Design and methods of a double blind randomized placebo-controlled trial of extended-release naltrexone for HIV-infected, opioid dependent prisoners and jail detainees who are transitioning to the community. Contemporary Clinical Trials. 2014;39(2):256-68. | Exclude: Design and methods paper of a clinical trial; Nil discontinuation outcomes reported |
| 112. Digiusto E, Shakeshaft A, Ritter A, O'Brien S, Mattick RP. Serious adverse events in the Australian National Evaluation of Pharmacotherapies for Opioid Dependence (NEPOD). Addiction. 2004;99(4):450-60. | Exclude: Review of trials and SAEs on/off pharmacotherapy. Prevalence reported by individual trials |
| 113. Dijkstra BA, De Jong CA, Bluschke SM, Krabbe PF, van der Staak CP. Does naltrexone affect craving in abstinent opioid-dependent patients? Addiction Biology. 2007;12(2):176-82. | Exclude: Reports on craving outcomes; No empirical data on naltrexone initiation or discontinuation reported |
| 114. Dijkstra BA, De Jong CA, Krabbe PF, van der Staak CP. Prediction of abstinence in opioid-dependent patients. Journal of Addiction Medicine. 2008;2(4):194-201. | Exclude: Examines predictors of relapse; No empirical data of naltrexone initiation or discontinuation reported |
| 115. Dijkstra BA, De Jong CA, Wensing M, Krabbe PF, van der Staak CP. Opioid detoxification: from controlled clinical trial to clinical practice. American Journal on Addictions. 2010;19(3):283-90. | Exclude: Study on withdrawal does not report a calculable rate of naltrexone discontinuation |
| 116. Dijkstra BAG, Krabbe PFM, De Jong CAJ, van der Staak CPF. Prediction of withdrawal symptoms during opioid detoxification. Journal of Opioid Management. 2008;4(5):311-9. | Exclude: Examines predictors of withdrawal; No empirical data of naltrexone initiation or discontinuation reported |
| 117. Douaihy A, Akerman S, Legedza A, Sullivan M, Zavod A, Fratantonio J. Characteristics of individuals seeking to transition from BUP to XR-NTX in a randomized, placebo-controlled trial. American Journal on Addictions. 2020;29(3):231. | Exclude: Conference Abstract; No empirical data reported on the prevalence of or the factors associated with naltrexone initiation/discontinuation. Dispensing data reported and unclear within selected cohort the number eligible for naltrexone. Reasons for wanting to transition to antagonist therapy |
| 118. Douaihy AB, Zavod A. Withdrawal Patterns during Transition from Opioid Use or Buprenorphine Treatment to XR-NTX. Journal of Addiction Medicine. 2020;14(6):e423-e4. | Exclude: Conference Abstract; No empirical data reported on the prevalence of or the factors associated with naltrexone initiation/discontinuation |
| 119. Ducharme LJ, Knudsen HK, Roman PM. Evidence-based treatment for opiate-dependent clients: Availability, variation, and organizational correlates. American Journal of Drug and Alcohol Abuse. 2006;32(4):569-76. | Exclude: Examines availability of treatment modalities; Nil patient initation or discontinuation data |
| 120. Dunn K, DeFulio A, Everly JJ, Donlin WD, Aklin WM, Nuzzo PA, et al. Employment-based reinforcement of adherence to oral naltrexone in unemployed injection drug users: 12-month outcomes. Psychology of Addictive Behaviors. 2015;29(2):270-6. | Exclude: 12 month follow up data of screened clinical trial Dunn 2013. |
| 121. Elman I, D'Ambra MN, Krause S, Breiter H, Kane M, Morris R, et al. Ultrarapid opioid detoxification: Effects on cardiopulmonary physiology, stress hormones and clinical outcomes. Drug and Alcohol Dependence. 2001;61(2):163-72. | Exclude: Paper assessing withdrawal; All patients aimed to be inducted onto naltrexone following detoxification; No prevalence estimate of number of patients opting to initiate naltrexone calculable. Nil calculable proportion of discontinuation of naltrexone reported |
| 122. Estreet A, Garber C, Vo H, Fishman M, Burgower R. Continuing care engagement following inpatient treatment for youth with opioid addiction. Journal of Addiction Medicine. 2016;10(3):E11-E2. | Exclude: Conference abstract using data from included study Vo 2016. Exclude to avoid double counting. |
| 123. Fals-Stewart W, O'Farrell TJ. Behavioral family counseling and naltrexone for male opioid-dependent patients. Journal of Consulting and Clinical Psychology. 2003;71(3):432-42. | Exclude: Clinical trial reporting relapse and days of naltrexone usage “took naltrexone on more days during treatment (M 102.6, SD  41.3) than IBT patients M79.4,SD46.3), F(1,122)7.83,2 .06,p.01” Does not report data enabling calculable rate of naltrexone discontinuation. |
| 124. Farabee D, Condon T, Hallgren KA, McCrady B. A randomized comparison of extended-release naltrexone with or without patient navigation vs enhanced treatment-as-usual for incarcerated adults with opioid use disorder. Journal of Substance Abuse Treatment. 2020;117:108076. | Exclude: Unable to calculate numbers of people receiving number of XR-NTX injections. Reports mean number of injections including those that did not receive >-1 injection; Baseline numbers of people receiving at least one injection calculable for XR-NTX and XR-NTX-PN groups however discontinuation only reported for those not receiving all seven injections (not a timeframe studied here) “Across both conditions, only 8% of participants (7 of 91) received all seven possible injections” |
| 125. Fareed A, Vayalapalli S, Casarella J, Amar R, Drexler K. Heroin anticraving medications: a systematic review. Am J Drug Alcohol Abuse. 2010;36(6):332-41. | Exclude: Review article all references checked for inclusion |
| 126. Farren CK, O'Malley S. A pilot double blind placebo controlled trial of sertraline with naltrexone in the treatment of opiate dependence. American Journal on Addictions. 2002;11(3):228-34. | Exclude: Reports retention in treatment only; No empirical discontinuation data reported |
| 127. Favrat B, Zimmermann G, Zullino D, Krenz S, Dorogy F, Muller J, et al. Opioid antagonist detoxification under anaesthesia versus traditional clonidine detoxification combined with an additional week of psychosocial support: a randomised clinical trial. Drug Alcohol Depend. 2006;81(2):109-16. | Exclude: Does not report denominators of eligible patients who have been detoxified who were offered naltrexone therapy, Does not report number of patients discontinuing in RODA group. “In the clonidine group, after the in-patient stay, 2/34 started naltrexone [unclear number who were detoxed fully and offered naltrexone], compared to 24/36 in the anaesthesia-assisted group (p = 0.001) [again unclear number detoxed and eligible]. The mean number of days of naltrexone intake after the end of detoxification treatment was 10.8 (IC 95%: 5.2–16.4) for the RODA group. In the clonidine group one patient took the drug for 3 months.] |
| 128. Festinger DS, Dugosh KL, Gastfriend DR, Sierka C. Attitudes and practices on the use of extended-release naltrexone in criminal justice settings. Drug and Alcohol Dependence. 2017;Conference: 2016 Annual Meeting of the College on Problems of Drug Dependence, CPDD 2016. Palm Springs, CA United States. 171:e62-e3. | Exclude: Conference abstract of cross sectional survey to providers of XR-NTX assessing attitudes |
| 129. Fiellin D, Cutter CJ, Moore BA, Barry D, O'Connor P, Schottenfeld RS. Primary care buprenorphine detoxification vs. Maintenance for prescription opioid dependence. Drug and Alcohol Dependence. 2015;Conference: 2014 Annual Meeting of the College on Problems of Drug Dependence. San Juan Puerto Rico. 146:e277. | Exclude: Conference abstract of maintenance versus detoxification strategies |
| 130. Fiellin DA, Schottenfeld RS, Cutter CJ, Moore BA, Barry DT, O'Connor PG. Primary care-based buprenorphine taper vs maintenance therapy for prescription opioid dependence: a randomized clinical trial. JAMA Internal Medicine. 2014;174(12):1947-54. | Exclude: Clinical trial with one arm buprenorphine taper to abstinence followed by opioid free period and offer of naltrexone. Unclear number of people randomised to this arm eligible for naltrexone (i.e. completed detoxification and opioid free). No naltrexone discontinuation data presented |
| 131. Fingerhood MI, Thompson MR, Jasinski DR. A comparison of clonidine and buprenorphine in the outpatient treatment of opiate withdrawal. Substance Abuse. 2001;22(3):193-9. | Exclude: Paper assessing withdrawal; All patients aimed to be inducted onto naltrexone following detoxification; No prevalence estimate of number of patients opting to initiate naltrexone calculable. Nil discontinuation of naltrexone data reported. |
| 132. Firth AY, Pulling S, Carr MP, Beaini AY. Orthoptic status before and immediately after heroin detoxification. Br J Ophthalmol. 2004;88(9):1186-90. | Exclude: Paper assessing withdrawal; All patients aimed to be inducted onto naltrexone following detoxification; No prevalence estimate of number of patients opting to initiate naltrexone calculable. Nil discontinuation of naltrexone data reported. |
| 133. Fishman M, Selby V, Wildberger J, Lavorato L, Thomas J, Moody S, et al. Extending the youth opioid recovery support (YORS) intervention with patient choice of extended-release naltrexone or extended-release buprenorphine. American Journal on Addictions. 2021;30(3):256. | Exclude: Conference Abstract; No empirical data reported on the prevalence of or the factors associated with naltrexone initiation/discontinuation. XR-NTX cohort with no discontinuation data presented. |
| 134. Flanagan Balawajder E, Ducharme L, Taylor BG, Lamuda PA, Kolak M, Friedmann PD, et al. Factors Associated With the Availability of Medications for Opioid Use Disorder in US Jails. JAMA Network Open. 2024;7(9):e2434704. | Exclude: Cross sectional survey of jails in the US and availability of medication for opioid use disorder |
| 135. Foy A, Sadler C, Taylor A. An open trial of naltrexone for opiate dependence. Drug and Alcohol Review. 1998;17(2):167-74. | Exclude: Reports retention in treatment  at 12 months “Retention at 12 months was 34% but it is noted that [of these] only two patients actually took naltrexone for the whole of the 6 months as intended” unable to calculate rate of discontinuation |
| 136. Friedmann PD, Dunn KE, Nunes EV, O'Brien CP. Effectiveness, implementation and real-world experience with extended-release naltrexone (XR-NTX): A special issue of JSAT. Journal of Substance Abuse Treatment. 2018;85:31-3. | Exclude: Editorial on XR-NTX use |
| 137. Friedmann PD, Lee JD, Nunes EV, Kinlock TW, O'Brien CP. Patient selection for extended-release naltrexone among criminal justice-involved persons with opioid use disorder. Drug and Alcohol Dependence. 2015;Conference: 2015 Annual Meeting of the College on Problems of Drug Dependence, CPDD 2015. Phoenix, AZ United States. 156:e74-e5. | Exclude: Conference abstract assessing predictors of opioid free state and ability to initiate naltrexone |
| 138. Friedmann PD, Wilson D, Nunes EV, Hoskinson R, Jr., Lee JD, Gordon M, et al. Do patient characteristics moderate the effect of extended-release naltrexone (XR-NTX) for opioid use disorder? Journal of Substance Abuse Treatment. 2018;85:61-5. | Exclude: Secondary analyses if screened clinical trial reporting on moderators or relapse to opioid use |
| 139. Frost MC, Lampert H, Tsui JI, Iles-Shih MD, Williams EC. The impact of methamphetamine/amphetamine use on receipt and outcomes of medications for opioid use disorder: a systematic review. Addict Sci Clin Pract. 2021;16(1):62. | Exclude: Review article all articles checked for inclusion |
| 140. Gainer DM, Nahhas RW, Vanderhoof T, Silverstein SM, Wright MD, Vanderhoof SO, et al. Exploring the interactions between non-medical methamphetamine use and prescribed buprenorphine or naltrexone in opioid use disorder treatment retention. Substance Use & Misuse. 2021;56(14):2160-70. | Exclude: Evaluated treatment retention, as measured by time to dropout, not discontinuation of naltrexone |
| 141. García-Alonso F, Gutierrez M, San L, Bedate J, Forteza-Rei J, Rodríguez-Artalejo F, et al. A multicentre study to introduce naltrexone for opiate dependence in Spain. Drug and alcohol dependence. 1989;23(2):117-21. | Exclude: Retention in treatment “‘Therapeutic failure’ was considered in the following circumstances: (a) when during the course of the study the patient failed to attend the established pattern of visits on at least five consecutive occasions; (b) when during the course of study the patient failed to attend the established pattern of visits on at least ten alternative occasions; (cl when five or more urine samples were opiate positive.” No naltrexone discontinuation data reported. |
| 142. Gastfriend D, Silverman B, Memisoglu A, Krupitsky E, Ling W. Continuity of clinical efficacy with injectable extended-release naltrexone (XR-NTX). Journal of Addiction Medicine. 2013;7(4):E4. | Exclude: Conference abstract of included data from Krupitsky 2011. |
| 143. Gastfriend DR. Intramuscular extended-release naltrexone: current evidence. Annals of the New York Academy of Sciences. 2011;1216:144-66. | Exclude: Review article in alcohol dependence |
| 144. Gastfriend DR, Krupitsky E, Nunes EV, Ling W, Illeperuma A, Silverman BL. Efficacy and safety of injectable extended-release naltrexone (XR-NTX) for the treatment of opioid dependence. American Journal on Addictions. 2011;20(4):389. | Exclude: Conference abstract of clinical trial reporting treatment completion rates no calculable rate of naltrexone discontinuation reported |
| 145. Gaulen Z, Brenna IH, Fadnes LT, Saltyte Benth J, Solli KK, Kunoe N, et al. The Predictive Value of Degree of Preference for Extended-Release Naltrexone for Treatment Adherence, Opioid Use, and Relapse. European Addiction Research. 2022;28(1):56-67. | Exclude: Secondary analysis of screened clinical trial assessing associations with treatment preference. |
| 146. Gauthier P, Greco P, Meyers-Ohki S, Desai A, Rotrosen J. Patients' perspectives on initiating treatment with extended-release naltrexone (XR-NTX). Journal of Substance Abuse Treatment. 2021;122:108183. | Exclude: Qualitative study of experiences of induction onto XR-NTX |
| 147. George S, Ekhtiari H. Naltrexone in the treatment of opioid dependence. British Journal of Hospital Medicine. 2010;71(10):568-70. | Exclude: Review article all references cheeked for inclusion |
| 148. Gerra G, Ceresini S, Esposito A, Zaimovic A, Moi G, Bussandri M, et al. Neuroendocrine and behavioural responses to opioid receptor-antagonist during heroin detoxification: relationship with personality traits. Int Clin Psychopharmacol. 2003;18(5):261-9. | Exclude: Paper assessing withdrawal; All patients aimed to be inducted onto naltrexone following detoxification; No prevalence estimate of number of patients opting to initiate naltrexone calculable. Nil discontinuation of naltrexone data reported. |
| 149. Gerra G, Marcato A, Caccavari R, Fontanesi B, Delsignore R, Fertonani G, et al. Clonidine and opiate receptor antagonists in the treatment of heroin addiction. Journal of Substance Abuse Treatment. 1995;12(1):35-41. | Exclude: Paper assessing withdrawal strategies; All patients aimed to be inducted onto naltrexone following detoxication; Nil number of eligible patients post detoxification initiating naltrexone reported. Reports clinic attendance at six months not naltrexone discontinuation data. “”attendance to the meetings demonstrated a significantly higher percentage” |
| 150. Gibson AE, Doran CM, Bell JR, Ryan A, Lintzeris N. A comparison of buprenorphine treatment in clinic and primary care settings: a randomised trial. Medical Journal of Australia. 2003;179(1):38-42. | Exclude: Initiation data reported in pooled included Digustio et al. No discontinuation data reported. |
| 151. Ginzburg HM. Naltrexone: its clinical utility. Adv Alcohol Subst Abuse. 1985;5(1-2):83-101. | Exclude: Review article: No empirical data reported on the prevalence of or the factors associated with naltrexone initiation/discontinuation |
| 152. Ginzburg HM, Glass WJ. The role of the National Institute on Drug Abuse in the development of naltrexone. Journal of Clinical Psychiatry. 1984;45(9 Pt 2):4-6. | Exclude: Duplicate of included Washton study |
| 153. Ginzburg HM, MacDonald MG. The role of naltrexone in the management of drug abuse. Medical Toxicology and Adverse Drug Experience. 1987;2(2):83-92. | Exclude: Review article all references checked |
| 154. Gish EC, Miller JL, Honey BL, Johnson PN. Lofexidine, an alpha<inf>2</inf>-receptor agonist for opioid detoxification. Annals of Pharmacotherapy. 2010;44(2):343-51. | Exclude: Examines lofexidine for withdrawal; Does not report a calculable rate of naltrexone for relapse prevention discontinuation |
| 155. Gold CG, Cullen DJ, Gonzales S, Houtmeyers D, Dwyer MJ. Rapid opioid detoxification during general anesthesia: a review of 20 patients. Anesthesiology. 1999;91(6):1639-47. | Exclude: Paper assessing withdrawal; All patients aimed to be inducted onto naltrexone following detoxification; No prevalence estimate of number of patients opting to initiate naltrexone calculable. Nil discontinuation of naltrexone data reported. |
| 156. Gold MS, Dackis CA, Pottash AL, Sternbach HH, Annitto WJ, Martin D, et al. Naltrexone, opiate addiction, and endorphins. Med Res Rev. 1982;2(3):211-46. | Exclude: Review article: No empirical data reported on the prevalence of or the factors associated with naltrexone initiation/discontinuation |
| 157. Gold MS, Dackis CA, Washton AM. The sequential use of clonidine and naltrexone in the treatment of opiate addicts. Adv Alcohol Subst Abuse. 1984;3(3):19-39. | Exclude: Empirical data reported is duplicate of that in Washton 1984 114 business professionals/16 physicians. Exclude to avoid double counting. |
| 158. Gonzalez JP, Brogden RN. Naltrexone. A review of its pharmacodynamic and pharmacokinetic properties and therapeutic efficacy in the management of opioid dependence. Drugs. 1988;35(3):192-213. | Exclude: Review article all references checked |
| 159. Gooberman LL, Bradway DW. Depot naltrexone vs oral naltrexone postdetoxification. Journal of Addictive Diseases. 1998;17(2):150. | Exclude: Conference abstract; No empirical data reported on the prevalence of or the factors associated with naltrexone initiation/discontinuation |
| 160. Gordon MS, Mitchell SG, Blue TR, Vocci FJ, Fishman MJ, Murphy SM, et al. A clinical protocol of a comparative effectiveness trial of extended-release naltrexone versus extended-release buprenorphine with individuals leaving jail. Journal of Substance Abuse Treatment. 2021;128:108241. | Exclude: Protocol paper |
| 161. Gowing L, Ali R, White J. Opioid antagonists and adrenergic agonists for the management of opioid withdrawal. Cochrane Database of Systematic Reviews. 2000(2):CD002021. | Exclude: Cochrane review of withdrawal regimens including naltrexone; All references checked |
| 162. Gowing L, Ali R, White J. Opioid antagonists with minimal sedation for opioid withdrawal. Cochrane Database of Systematic Reviews. 2002(2):CD002021. | Exclude: Cochrane review of opioid antagonists for withdrawal: All references checked |
| 163. Gowing L, Ali R, White J. Opioid antagonists under heavy sedation or anaesthesia for opioid withdrawal. Cochrane Database of Systematic Reviews. 2006(2):CD002022. | Exclude: Review of withdrawal practices; All references checked |
| 164. Gowing L, Ali R, White JM. Buprenorphine for the management of opioid withdrawal. Cochrane Database of Systematic Reviews. 2009;(3) (no pagination). | Exclude: Review of buprenorphine for withdrawal all references checked |
| 165. Gowing L, Ali R, White JM. Opioid antagonists with minimal sedation for opioid withdrawal. Cochrane Database of Systematic Reviews. 2009(4):CD002021. | Exclude: Review of withdrawal; all references checked |
| 166. Gowing L, Ali R, White JM. Opioid antagonists under heavy sedation or anaesthesia for opioid withdrawal. Cochrane Database of Systematic Reviews. 2010(1):CD002022. | Exclude: Review article all references checked |
| 167. Gowing L, Ali R, White JM. Opioid antagonists with minimal sedation for opioid withdrawal. Cochrane Database of Systematic Reviews. 2017;5:CD002021. | Exclude: Review article assessing withdrawal and induction onto naltrexone. Nil data on number opting for initiation or discontinuation data presented |
| 168. Green A, Kaul A, O'Shea J, Sharma E, Bennett L, Mullings EL, et al. Opiate agonists and antagonists modulate taste perception in opiate-maintained and recently detoxified subjects. Journal of Psychopharmacology. 2013;27(3):265-75. | Exclude: No empirical data reported to allow calculation of proportion of people discontinuing naltrexone |
| 169. Greenstein R, O’Brien C, Mintz J, Woody G, Hanna N. Clinical experience with naltrexone in a behavioral research study: An interim report. Narcotic Antagonists: Naltrexone, NIDA Research Monograph. 1976;9:141-9. | Exclude: Report superseded by reports from same patient sample Greenstein 1981/1984 reporting on expanded patient population. Excluded to prevent double counting. |
| 170. Greenstein RA, Evans BD, McLellan AT, O'Brien CP. Predictors of favorable outcome following naltrexone treatment. Drug and Alcohol Dependence. 1983;12(2):173-80. | Exclude: Reports on subsample of patients selected from Greenstein’s reports 1984/1981/1976; Included study with largest sample size to avoid double counting. |
| 171. Greiner MG, Shulman M, Choo TH, Scodes J, Pavlicova M, Campbell ANC, et al. Naturalistic follow-up after a trial of medications for opioid use disorder: Medication status, opioid use, and relapse. Journal of Substance Abuse Treatment. 2021;131:108447. | Exclude: 36 week follow up results of screened clinical trial (X:BOT) outcomes described beyond studies outcome timeframe (36 weeks) |
| 172. Greiner MG, Shulman M, Opara O, Potter K, Voronca DC, Tafessu HM, et al. Surmounting Withdrawal to Initiate Fast Treatment with Naltrexone (SWIFT): A stepped wedge hybrid type 1 effectiveness-implementation study. Contemporary Clinical Trials. 2023;128:107148. | Exclude: Methods paper for screened clinical trial |
| 173. Grey C, Osborn E, Reznikoff M. Psychosocial factors in outcome in two opiate addiction treatments. Journal of Clinical Psychology. 1986;42(1):185-9. | Exclude: Duplicate cohort of excluded Osborn 1986. Reports retention as measured by clinic attendance “Retention was calculated by number of required clinic appointments kept during the 12-week period of the study” |
| 174. Guo S, Jiang Z, Wu Y. Efficacy of naltrexone hydrochloride for preventing relapse among opiate-dependent patients after detoxification. Hong Kong Journal of Psychiatry. 2001;11(4):2-8+28. | Exclude: Reports retention at six months in the open trial of the study; Nil specific discontinuation data reported with unclear denominator for the number of patients taking naltrexone in the open study. No discontinuation data reported in the double blind study. “The retention rate of naltrexone at 6 months was 23.6%” |
| 175. Guthrie SK. Pharmacologic interventions for the treatment of opioid dependence and withdrawal. Dicp. 1990;24(7-8):721-34. | Exclude: Review article; References checked; No empirical data reported on the prevalence of or the factors associated with naltrexone initiation/discontinuation. |
| 176. Gutierrez M, Ballesteros J, Gonzalez-Oliveros R, Ruiz de Apodaka J. Retention rates in two naltrexone programmes for heroin addicts in Vitoria, Spain. European Psychiatry. 1995;10(4):183-8. | Exclude: Reports retention not naltrexone discontinuation “A drop-out was defined as the following: a self-discharge from the program against medical advice; or breaking treatment rules, in particular, unjustified absence to five consecutive or ten alternative follow-up visits, and/or the presence of opiate contaminated urine on five occasions.” |
| 177. Haase KS, Opheim A, Kunoe N, Stavseth LS, Latif ZEH, Mjolhus ALN, et al. Interest for treatment with extended release Naltrexone among Norwegian opiate users. Drug and Alcohol Dependence. 2015;Conference: 2014 Annual Meeting of the College on Problems of Drug Dependence. San Juan Puerto Rico. 146:e132. | Exclude: Conference abstract of cross-sectional survey of people with opioid dependence to assess interest in XR-NTX |
| 178. Hadland SE, Bagley SM, Rodean J, Levy S, Zima BT. Use of evidence-based medication treatment among medicaid-enrolled youth with opioid use disorder, 2014–2015. Journal of Adolescent Health. 2018;62(2):S16. | Exclude: Reports Number of people in receipt of medication for opioid use disorder among a claims database; Unclear in denominator whom is eligible for naltrexone as a relapse prevention medication. |
| 179. Hadland SE, Bagley SM, Rodean J, Silverstein M, Levy S, Larochelle MR, et al. Receipt of Timely Addiction Treatment and Association of Early Medication Treatment with Retention in Care among Youths with Opioid Use Disorder. JAMA Pediatrics. 2018;172(11):1029-37. | Exclude: Does not report number of patients detoxed and eligible to receive naltrexone who were offered and initiated it; Reports retention in treatment defined as time from receipt of first addiction treatment (either behavioural health services or OUD medication) to time of treatment discontinuation; Does not report discontinuation rates for individual drugs just category of medication for opioid use disorder overall |
| 180. Hadland SE, Wharam JF, Schuster MA, Zhang F, Samet JH, Larochelle MR. Trends in receipt of buprenorphine and naltrexone for opioid use disorder among adolescents and young adults, 2001-2014. JAMA pediatrics. 2017;171(8):747-55. | Exclude: Reports Number of people in receipt of medication for opioid use disorder among a claims database; Unclear in denominator whom is eligible for naltrexone as a relapse prevention medication. |
| 181. Haeny AM, Montgomery L, Burlew AK, Campbell ANC, Scodes J, Pavlicova M, et al. Extended-release naltrexone versus buprenorphine-naloxone to treat opioid use disorder among black adults. Addictive Behaviors. 2020;110:106514. | Exclude: Secondary analysis of screened clinical trial reporting on outcomes in people of black ethnicity. |
| 182. Hall W, Mattick RP. Is ultra-rapid opioid detoxification a viable option in the treatment of opioid dependence? CNS Drugs. 2000;14(4):251-5. | Exclude: Review article all references checked for includable studies |
| 183. Hartung DM, McCarty D, Fu R, Wiest K, Chalk M, Gastfriend DR. Extended-release naltrexone for alcohol and opioid dependence: A meta-analysis of healthcare utilization studies. Journal of Substance Abuse Treatment. 2014;47(2):113-21. | Exclude: Review article all references checked for inclusion |
| 184. Haug NA, Sorensen JL, Gruber VA, Song YS. Relapse Prevention for Opioid Dependence.  Relapse prevention: Maintenance strategies in the treatment of addictive behaviors , 2nd ed. New York, NY: The Guilford Press; US; 2005. p. 151-78. | Exclude: Review article all references checked |
| 185. He S, Yu L, Jia S, Chen Q, Wang D, Hu S. Effects of long-term sustained naltrexone release on the optic center in opioid-dependent patients: Case-control study in four provinces of China. Neural Regeneration Research. 2011;6(3):236-40. | Exclude: Does not report empirical data to provide calculable rate of naltrexone discontinuation |
| 186. He SX, Yu LC, Chen Q, Wang DM, Hu S, Jia SW. Effect of long-term sustained release naltrexone on semantic recognition of opioid addicts. Journal of Clinical Rehabilitative Tissue Engineering Research. 2009;13(8):1573-6. | Exclude: No empirical data reported to for calculable naltrexone initiation or discontinuation. |
| 187. Helm S, Trescot AM, Colson J, Sehgal N, Silverman S. Opioid antagonists, partial agonists, and agonists/antagonists: the role of office-based detoxification. Pain physician. 2008;11(2):225-35. | Exclude: Review article all references checked for inclusion |
| 188. Hensel M, Kox WJ. Safety, efficacy, and long-term results of a modified version of rapid opiate detoxification under general anaesthesia: a prospective study in methadone, heroin, codeine and morphine addicts. Acta Anaesthesiol Scand. 2000;44(3):326-33. | Exclude: Paper assessing withdrawal strategies; All patients aimed to be inducted onto naltrexone following detoxication; Nil other treatment options available and no prevalence estimate of number of patients opting to initiate naltrexone calculable. Nil naltrexone discontinuation data reported. |
| 189. Herbeck DM, Jeter KE, Cousins SJ, Abdelmaksoud R, Crevecoeur-MacPhail D. Gender differences in treatment and clinical characteristics among patients receiving extended release naltrexone. Journal of Addictive Diseases. 2016;35(4):305-14. | Exclude: Same data source and timeframe of data collection as Cousins 2016. Exclude to avoid double counting. |
| 190. Hermes G, Hyman SM, Fogelman N, Kosten TR, Sinha R. Lofexidine in Combination With Oral Naltrexone for Opioid Use Disorder Relapse Prevention: A Pilot Randomized, Double-Blind, Placebo-Controlled Study. American Journal on Addictions. 2019;28(6):480-8. | Exclude: Reports number of days patients were blocked with naltrexone across the 84‐day study period and retention in treatment not calculable rate of naltrexone discontinuation. |
| 191. Hickman M, Degenhardt L, Farrell M, Hall W. Commentary on Kelty & Hulse (2012): Is the comparison of mortality between patients prescribed implanted or oral naltrexone an unbiased and unconfounded comparison? Addiction. 2012;107(10):1825-6. | Exclude: Commentary article all references checked |
| 192. Hoque MM, Hossain KJ, Kamal MM, Akhtaruzzaman M. Naltrexone in drug addiction: significance in the prevention of relapse. Mymensingh Med J. 2009;18(1 Suppl):S56-65. | Exclude: No empirical data reported to for calculable naltrexone initiation or discontinuation. |
| 193. Horspool MJ, Seivewright N, Armitage CJ, Mathers N. Post-treatment outcomes of buprenorphine detoxification in community settings: a systematic review. European Addiction Research. 2008;14(4):179-85. | Exclude: Review article all references checked for inclusion |
| 194. Hulse G, O'neil G. Using naltrexone implants in the management of the pregnant heroin user. Australian and New Zealand Journal of Obstetrics and Gynaecology. 2002;42(5):569-73. | Exclude: Case series of 8 patients in pregnancy treated with naltrexone implants; No data given with regards initiation and discontinuation rates incalculable for cohort - not all cases report number of implants used or removed |
| 195. Hulse GK, Morris N, Arnold-Reed D, Tait RJ. Improving clinical outcomes in treating heroin dependence: randomized, controlled trial of oral or implant naltrexone. Archives of General Psychiatry. 2009;66(10):1108-15. | Exclude: Clinical trail with unclear reporting with regards discontinuation of oral or implant naltrexone: Paper states “trial participation was discontinued and an alternative treatment was offered (figure 1)”. Figure one reports discontinuation from trial but unclear point of discontinuation or if naltrexone implant still in situ (in only one case is this definitively reported as being explanted”. in oral group unclear when or if naltrexone discontinued. |
| 196. Hulse GK, Ngo HT, Tait RJ. Risk factors for craving and relapse in Heroin users treated with oral or implant naltrexone. Biological Psychiatry. 2010;68(3):296-302. | Exclude: Does not report a calculable rate of naltrexone for relapse prevention discontinuation |
| 197. Hulse GK, Tait RJ. A pilot study to assess the impact of naltrexone implant on accidental opiate overdose in 'high-risk' adolescent heroin users. Addiction Biology. 2003;8(3):337-42. | Exclude: Reports data on 8 adolescents receiving naltrexone implants and overdose risk associated; All patients aimed to be inducted onto naltrexone following detoxification; No prevalence estimate of number of patients opting to initiate naltrexone calculable. Nil discontinuation of naltrexone data calculable as different implant durations reported not able to calculate rate of implant discontinuation. |
| 198. Hyman SM, Fox H, Hong KI, Doebrick C, Sinha R. Stress and drug-cue-induced craving in opioid-dependent individuals in naltrexone treatment. Exp Clin Psychopharmacol. 2007;15(2):134-43. | Exclude: Reports on craving outcomes; No empirical data on naltrexone initiation or discontinuation reported |
| 199. Hyman SM, Hong KI, Chaplin TM, Dabre Z, Comegys AD, Kimmerling A, et al. A stress-coping profile of opioid dependent individuals entering naltrexone treatment: a comparison with healthy controls. Psychology of Addictive Behaviors. 2009;23(4):613-9. | Exclude: Reports psychological measurers of stress; No empirical data reported to for calculable naltrexone initiation or discontinuation. |
| 200. Iosub R, Seeger I, Law F, Wallbank N, Melichar J. Substantial improvement in outpatient opiate-free exits using a novel resource efficient lofexidine and buprenorphine-based protocol ('Detox-in-a-Box') - a service evaluation study. Heroin Addiction and Related Clinical Problems. 2017;19(4):5-12. | Exclude: Does not report the numbers of people opting to initiate naltrexone nor rate of naltrexone discontinuation |
| 201. Iosub R, Seeger I, Law FD, Wallbank NS, Melichar JK. Dramatic improvem ent in outpatient opiate-free exits using a novel resource-efficient lofexidine-based protocol (Bristol-regim e)-a service evaluation study. European Psychiatry. 2015;1):475. | Exclude: Conference abstract of study of novel withdrawal protocol |
| 202. Iraurgi I, Jimenez-Lerma JM, Landabaso MA, Arrazola X, Gutierrez-Fraile M. Gypsies and drug addictions. Study of the adherence to treatment. European Addiction Research. 2000;6(1):34-41. | Exclude: Reports retention as measured by “abandoning treatment” defined as “‘ (a) failing to report for two consecutive or three alternate counselling sessions, in the absence of justification; (b) failing to report for two consecutive (or three alternate) urine samplings; (c) opioids detected in urine on five occasions in a 12-week period; or (d) three consecutive positive urinalyses” Naltrexone discontinuation rate not reported. |
| 203. IuP S, Savchenkov V. Preventive therapy of opiate addiction with naltrexone. Zhurnal Nevrologii i Psikhiatrii Imeni SS Korsakova. 1998;98(11):22-5. | Exclude: RCT does not report data for calculation of discontinuation |
| 204. Jaffe JH. Do antagonists have a role in the treatment of opioid dependence? Addiction. 2006;101(4):468-9. | Exclude: Editorial all references checked for inclusion |
| 205. Jalali A, Jeng PJ, Polsky D, Poole S, Ku YC, Woody GE, et al. Cost-effectiveness of extended-release injectable naltrexone among incarcerated persons with opioid use disorder before release from prison versus after release. Journal of Substance Abuse Treatment. 2022;141:108835. | Exclude: Cost effectiveness analysis od screened clinical trial |
| 206. Jarvis BP, Holtyn AF, Berry MS, Subramaniam S, Umbricht A, Fingerhood M, et al. Predictors of induction onto extended-release naltrexone among unemployed heroin-dependent adults. Journal of Substance Abuse Treatment. 2018;85:38-44. | Exclude: Reports predictors of successful induction onto XR-NTX from oral naltrexone. Not opting to initiate or discontinuation of naltrexone data reported |
| 207. Jarvis BP, Holtyn AF, DeFulio A, Dunn KE, Everly JJ, Leoutsakos JS, et al. Effects of incentives for naltrexone adherence on opiate abstinence in heroin-dependent adults. Addiction. 2017;112(5):830-7. | Exclude: Post hoc analysis combining data from three screened studies |
| 208. Jarvis BP, Holtyn AF, Subramaniam S, Tompkins DA, Oga EA, Bigelow GE, et al. Extended-release injectable naltrexone for opioid use disorder: a systematic review. Addiction. 2018;113(7):1188-209. | Exclude: Systematic Review all references checked for inclusion |
| 209. Jeffrey GP, MacQuillan G, Chua F, Galhenage S, Bull J, Young E, et al. Hepatitis C virus eradication in intravenous drug users maintained with subcutaneous naltrexone implants. Hepatology. 2007;45(1):111-7. | Exclude: Reports on HCV outcomes; No empirical data on naltrexone initiation or discontinuation reported |
| 210. Jelovac N, Milas M, Golik-Gruber V. Naltrexone is efficient in maintaining heroin abstinence of selected groups of addicts. Alcoholism. 2000;36(1):73-7. | Exclude: Reports relapse rates. No initiation or discontinuation data reported |
| 211. Johansson BA, Berglund M, Lindgren A. Efficacy of maintenance treatment with naltrexone for opioid dependence: A meta-analytical review. Addiction. 2006;101(4):491-503. | Exclude: Systematic Review looking at retention rates in treatment - all references checked for inclusion |
| 212. Johnson B, Faraone SV. Outpatient detoxification completion and one-month outcomes for opioid dependence: A preliminary study of a neuropsychoanalytic treatment in pain patients and addicted patients. Neuropsychoanalysis. 2013;15(2):145-60. | Exclude: Reports study of withdrawal and use of low dose naltrexone; Exclude: No empirical data reported to allow calculation of proportion of people discontinuing naltrexone |
| 213. Johnson BD, Alexander E, McCarthy P, Lincoln T. Extended-release naltrexone for opioid use disorder started during or following incarceration. Journal of Addiction Medicine. 2016;10(3):E3. | Exclude: Conference abstract of included study Lincoln 2018 with same timeframe of data collection |
| 214. Jones CM, Byrd DJ, Clarke TJ, Campbell TB, Ohuoha C, McCance-Katz EF. Characteristics and current clinical practices of opioid treatment programs in the United States. Drug and Alcohol Dependence. 2019;205(no pagination). | Exclude: Cross sectional survey of opioid treatment providers |
| 215. Jones CM, Shoff C, Blanco C, Losby JL, Ling SM, Compton WM. Association of Receipt of Opioid Use Disorder-Related Telehealth Services and Medications for Opioid Use Disorder With Fatal Drug Overdoses Among Medicare Beneficiaries Before and During the COVID-19 Pandemic. JAMA Psychiatry. 2023;80(5):508-14. | Exclude: Reports prescribing data receipt of XR-NTX; Nil discontinuation data reported |
| 216. Jones HE. Acceptance of naltrexone by pregnant women enrolled in comprehensive drug addiction treatment: an initial survey. American Journal on Addictions. 2012;21(3):199-201. | Exclude: Cross sectional survey of women’s attitudes toward naltrexone |
| 217. Jovanovic T, Lazarevic D, Nikolic G. Differences in depression severity and frequency of relapses in opiate addicts treated with methadone or opiate blocker after detoxification. Vojnosanitetski Pregled. 2012;69(4):326-32. | Exclude: Reports association of depressive symptoms among people with dependence on treatment. Does not report calculable rates of naltrexone discontinuation |
| 218. Judson BA, Carney TM, Goldstein A. Naltrexone treatment of heroin addiction: efficacy and safety in a double-blind dosage comparison. Drug and alcohol dependence. 1981;7(4):325-46. | Exclude: Clinical trial reporting time in treatment and retention drop out rate not rate of naltrexone discontinuation “drop-out during the first treatment”. Also reports summary statistics for total number of doses but not calculable rate of discontinuation “When total number of doses over all treatment periods (not shown in Fig. 2) in the two dosage groups were compared (Kolmogorov-Smirnov two- sample test), a significant difference (0.01 < p < 0.025)” |
| 219. Judson BA, Goldstein A. Naltrexone treatment of heroin addiction: One-year follow-up. Drug and Alcohol Dependence. 1984;13(4):357-65. | Exclude: Does not report the number of individuals detoxed from LAMM and eligible to receive naltrexone who initiated it. Results of discontinuation are presented only for 12 months post initiation - timepoint not part of studied review - Study with earlier data Judson 1981 screened for inclusion |
| 220. Kalkan O, Ogel K, Pozam G, Sari H. Rate and Predictors of Severe Withdrawal Symptoms in an Accelerated Opioid Detoxification Program. Psychiatry and Clinical Psychopharmacology. 2020;30(1):31-7. | Exclude: Reports study of withdrawal and induction onto naltrexone implant. No denominator of people opting for naltrexone treatment provided and no follow up or discontinuation data presented. |
| 221. Kaye AD, Gevirtz C, Bosscher HA, Duke JB, Frost EA, Richards TA, et al. Ultrarapid opiate detoxification: a review. Can J Anaesth. 2003;50(7):663-71. | Exclude: Review article all references checked |
| 222. Kelty E, Hulse G. Rates of Hospital and Emergency Department Attendances in Opiate-dependent Patients Treated with Implant Naltrexone, Methadone, or Buprenorphine. Addictive Disorders and their Treatment. 2017;16(2):39-48. | Exclude: Examines hopsitalisations of patients treated with medication for opioid use disorder. Nil empirical data of calculable rate of induction or discontinuation of naltrexone reported |
| 223. Kelty E, Hulse G. A Retrospective Cohort Study of Birth Outcomes in Neonates Exposed to Naltrexone in Utero: A Comparison with Methadone-, Buprenorphine- and Non-opioid-Exposed Neonates. Drugs. 2017;77(11):1211-9. | Exclude: Examines outcomes of neonates exposed to naltrexone in utero |
| 224. Kelty E, Hulse G. A retrospective cohort study of obstetric outcomes in opioid-dependent women treated with implant naltrexone, oral methadone or sublingual buprenorphine, and non-dependent controls. Drugs. 2017;77:1199-210. | Exclude: Does not reports rates of initiation or discontinuation of pregnant women on naltrexone therapy |
| 225. Kennedy-Hendricks A, Bandara S, Merritt S, Barry CL, Saloner B. Structural and organizational factors shaping access to medication treatment for opioid use disorder in community supervision. Drug Alcohol Depend. 2021;226:108881. | Exclude: Qualitative study; No empirical data on number of people initiating or discontinuing naltrexone |
| 226. Kienbaum P, Scherbaum N, Thurauf N, Michel MC, Gastpar M, Peters J. Acute detoxification of opioid-addicted patients with naloxone during propofol or methohexital anesthesia: a comparison of withdrawal symptoms, neuroendocrine, metabolic, and cardiovascular patterns. Crit Care Med. 2000;28(4):969-76. | Exclude: Paper assessing withdrawal; All patients aimed to be inducted onto naltrexone following detoxification; No prevalence estimate of number of patients opting to initiate naltrexone calculable. Nil discontinuation of naltrexone data reported. |
| 227. Kilaru AS, Perrone J, Meisel Z, Delgado M. Treatment for opioid use disorder following nonfatal overdose in patients with commercial insurance. Academic Emergency Medicine. 2020;27(Supplement 1):S135. | Exclude: Conference Abstract; No empirical data reported on the prevalence of or the factors associated with naltrexone initiation/discontinuation. Dispensing data reported and unclear within selected cohort the number eligible for naltrexone. |
| 228. Kim HK, Biary R, Chen BC, Berlin DA, Hoffman RS, Nelson LS, et al. Complications from ultra rapid opioid detoxification at a single detoxification center. Clinical Toxicology. 2013;51(4):347-8. | Exclude: Conference abstract of three cases of complications during ultra rapid opioid withdrawal |
| 229. Kirchmayer U, Davoli M, Verster A. Naltrexone maintenance treatment for opioid dependence. Cochrane Database of Systematic Reviews. 2000(2):CD001333. | Exclude: Cochrane review with more recent edition available |
| 230. Kirchmayer U, Davoli M, Verster A. Naltrexone maintenance treatment for opioid dependence. Cochrane Database of Systematic Reviews. 2003(2):CD001333. | Exclude: Cochrane review with more recent update available |
| 231. Kirchmayer U, Davoli M, Verster AD, Amato L, Ferri A, Perucci CA. A systematic review on the efficacy of naltrexone maintenance treatment in opioid dependence. Addiction. 2002;97(10):1241-9. | Exclude: Cochrane review with later version available |
| 232. Kirtadze I, Otiashvili D, O'Grady KE, Jones HE. Behavioral treatment + naltrexone reduces drug use and legal problems in the Republic of Georgia. Am J Drug Alcohol Abuse. 2012;38(2):171-5. | Exclude: Does not report calculable rates of naltrexone discontinuation |
| 233. Kjome KL, Gerard Moeller F. Long-acting injectable naltrexone for the management of patients with opioid dependence. Substance Abuse: Research and Treatment. 2011;5(1):1-9. | Exclude: Review article all references checked for inclusion |
| 234. Kleber HD. Ultrarapid opiate detoxification. Addiction. 1998;93(11):1629-33. | Exclude: Editorial on Ultra Rapid Detoxification |
| 235. Kleber HD. Pharmacologic treatments for heroin and cocaine dependence. American Journal on Addictions. 2003;12(s2):S5-S18. | Exclude: Review article all references checked |
| 236. Kleber HD. Pharmacologic treatments for opioid dependence: detoxification and maintenance options. Dialogues Clin Neurosci. 2007;9(4):455-70. | Exclude: Review article all references checked for inclusion |
| 237. Kleber HD, Kosten TR. Naltrexone induction: psychologic and pharmacologic strategies. Journal of Clinical Psychiatry. 1984;45(9 Pt 2):29-38. | Exclude: Review article all references checked for inclusion |
| 238. Kleber HD, Topazian M, Gaspari J, Riordan CE, Kosten T. Clonidine and naltrexone in the outpatient treatment of heroin withdrawal. Am J Drug Alcohol Abuse. 1987;13(1-2):1-17. | Exclude: Subsequent study Vining 1988 from same centre, same time period and same protocol with increased number of patients. Excluded to prevent double counting and Vining 1988 included. |
| 239. Klemperer EM, Hughes J. Does the efficacy of medications for substance abuse treatment decrease over time? Drug and Alcohol Dependence. 2015;Conference: 2014 Annual Meeting of the College on Problems of Drug Dependence. San Juan Puerto Rico. 146:e160. | Exclude: Conference abstract review of clinical trials with abstinence outcome |
| 240. Kosten TR. Combining family therapy with pharmacotherapy in opioid addicts: Use of naltrexone. International Journal of Family Psychiatry. 1986;7(2):167-77. | Exclude: No empirical data reported on the prevalence of or the factors associated with naltrexone initiation/discontinuation |
| 241. Kosten TR. Current pharmacotherapies for opioid dependence. Psychopharmacology Bulletin. 1990;26(1):69-74. | Exclude: Review article all references checked |
| 242. Kosten TR. Buprenorphine for Opioid Detoxification: A Brief Review. Addictive Disorders and their Treatment. 2003;2(4):107-12. | Exclude: Review of buprenorphine all references checked |
| 243. Kosten TR, Kleber HD. Strategies to improve compliance with narcotic antagonists. Am J Drug Alcohol Abuse. 1984;10(2):249-66. | Exclude: Review article all references checked for inclusion |
| 244. Kosten TR, Morgan C, Kleber HD. Phase II clinical trials of buprenorphine: detoxification and induction onto naltrexone. NIDA Res Monogr. 1992;121:101-19. | Exclude: No empirical data reported on the prevalence of or the factors associated with naltrexone initiation/discontinuation |
| 245. Kosten TR, Rounsaville BJ, Kleber HD. A 2.5-year follow-up of depression, life crises, and treatment effects on abstinence among opioid addicts. Archives of General Psychiatry. 1986;43(8):733-8. | Exclude: No empirical data reported on the prevalence of or the factors associated with naltrexone initiation/discontinuation |
| 246. Kresina TF, Pick B. Global availability and utilization of medication assisted treatment of drug abuse and dependence: An update. Frontiers in CNS Drug Discovery. 2010;1:625-37. | Exclude: Review article of medications; All available references checked |
| 247. Krupitsky E. Injectable extended-release naltrexone for the prevention of relapse to opioid dependence following opioid detoxification. Neuropsychiatry. 2012;2(4):355-62. | Exclude: Review article all references checked for inclusion |
| 248. Krupitsky E, Bisaga A, Nangia N, Sullivan M, Akerman S, Silverman B, et al. Extended-release naltrexone decreases opioid craving across the 4-week dosing interval. American Journal on Addictions. 2018;27(4):324-5. | Exclude: Secondary analysis of screened clinical trial reporting on craving outcomes |
| 249. Krupitsky E, Blokhina E, Zvartau E, Verbitskaya E, Lioznov D, Gross R, et al. P.148 Craving for opioids affects retention in treatment and adherence to antiretrovirals in hiv positive patients with oud treated with naltrexone. European Neuropsychopharmacology. 2019;29(Supplement 6):S117. | Exclude: Conference abstract of screened clinical trial reporting outcomes including retention in addiction treatment (number of addiction appointments kept) but not discontinuation. |
| 250. Krupitsky E, Blokhina E, Zvartau E, Verbitskaya E, Lioznov D, Yaroslavtseva T, et al. Slow-release naltrexone implant versus oral naltrexone for improving treatment outcomes in people with HIV who are addicted to opioids: a double-blind, placebo-controlled, randomised trial. Lancet HIV. 2019;6(4):e221-e9. | Exclude: Reports number of addiction appointments kept. Does not report number discontinuing from oral naltrexone nor the number of implants received . Does not allow a rate of discontinuation to be calculated. |
| 251. Krupitsky E, Illeperuma A, Gastfriend D, Silverman B. Efficacy and safety of extended-release injectable naltrexone for the treatment of opioid dependence. European Neuropsychopharmacology. 2011;3):S576. | Exclude: Conference abstract duplicate of Krupitsky 2011 |
| 252. Krupitsky E, Nunes EV, Ling W, Gastfriend DR, Memisoglu A, Silverman BL. Injectable extended-release naltrexone (XR-NTX) for opioid dependence: long-term safety and effectiveness. Addiction. 2013;108(9):1628-37. | Exclude: Reports time to discontinuation for groups at 1-year open label long term follow up of included clinical trial Krupitsky 2011 |
| 253. Krupitsky E, Woody GE, Zvartau E, O'Brien CP. Addiction treatment in Russia. The Lancet. 2010;376(9747):1145. | Exclude: Letter on commentary |
| 254. Krupitsky E, Zvartau E, Blokhina E, Verbitskaya E, Lioznov D, Yaroslavtseva T, et al. Naltrexone implant improves HIV TX outcomes VS oral NTX in opiate addicted patients. Topics in Antiviral Medicine. 2017;25(1 Supplement 1):368s-9s. | Exclude: Conference abstract of screened clinical trial reporting HIV outcomes; Nil empirical data of calculable rate of induction or discontinuation of naltrexone reported |
| 255. Krupitsky E, Zvartau E, Blokhina E, Verbitskaya E, Palatkin V, Tyurina A, et al. Anhedonia, depression, anxiety, and craving for opiates in opiate addicts stabilized on oral naltrexone and long acting naltrexone implant. Drug and Alcohol Dependence. 2015;Conference: 2015 Annual Meeting of the College on Problems of Drug Dependence, CPDD 2015. Phoenix, AZ United States. 156:e120. | Exclude: Conference abstract of screened clinical trial reporting on craving and anhedonia outcomes |
| 256. Krupitsky E, Zvartau E, Blokhina E, Verbitskaya E, Tsoy M, Wahlgren V, et al. Naltrexone with or without guanfacine for preventing relapse to opiate addiction in St.-Petersburg, Russia. Drug Alcohol Depend. 2013;132(3):674-80. | Exclude: Clinical trial: Reports medication compliance “and the proportion of riboflavin positive urines varied between 75 and 100%” and retention “defined as not missing two consecutive counselling sessions and not having relapsed”. No data able to calculate rate of naltrexone discontinuation reported. |
| 257. Krupitsky E, Zvartau E, Blokhina E, Verbitskaya E, Wahlgren V, Tsoy-Podosenin M, et al. Randomized trial of long-acting sustained-release naltrexone implant vs oral naltrexone or placebo for preventing relapse to opioid dependence. Archives of General Psychiatry. 2012;69(9):973-81. | Exclude: Clinical trial which reports relapse free retention in treatment and not discontinuation data. Number of implants received not reported nor data enabling calculation of discontinuation for oral naltrexone. |
| 258. Krupitsky E, Zvartau E, Blokhina E, Verbitskaya E, Wahlgren V, Tsoy-Podosenin M, et al. Anhedonia, depression, anxiety, and craving in opiate dependent patients stabilized on oral naltrexone or an extended release naltrexone implant. Am J Drug Alcohol Abuse. 2016;42(5):614-20. | Exclude: Secondary analysis of screened trial Krupitsky 2012; reports mental health and craving outcomes no discontinuation data |
| 259. Krupitsky E, Zvartau E, Blokhina E, Verbitskaya E, Woody G. Naltrexone for opioid dependence: Oral, implantable, and injectable. European Neuropsychopharmacology. 2012;2):S399-S400. | Exclude: Conference abstract of Krupitsky 2012 trial |
| 260. Krupitsky E, Zvartau E, Woody G. Use of naltrexone to treat opioid addiction in a country in which methadone and buprenorphine are not available. Current Psychiatry Reports. 2010;12(5):448-53. | Exclude: Review article all references checked for inclusion |
| 261. Krupitsky EM. Injectable extended-release naltrexone for the prevention of relapse to opioid dependence following opioid detoxification. Zhurnal Nevrologii i Psihiatrii imeni SS. 2014;Korsakova. 2014(5):64-72. | Exclude: Review article all references checked for inclusion |
| 262. Krupitsky EM, Blokhina EA. Long-acting depot formulations of naltrexone for heroin dependence: A review. Current Opinion in Psychiatry. 2010;23(3):210-4. | Exclude: Review article all references checked for inclusion |
| 263. Krupitsky EM, Zvartau EE, Masalov DV, Tsoi MV, Burakov AM, Egorova VY, et al. Naltrexone for heroin dependence treatment in St. Petersburg, Russia. Journal of Substance Abuse Treatment. 2004;26(4):285-94. | Exclude: Clinical trial reporting relapse free retention not discontinuation; “By the end of 6 376 months, 12 naltrexone patients (44.4%) remained in the 377 study and had not relapsed as compared to four (16%) in the 378 control group ( p < .05)” |
| 264. Krupitsky EM, Zvartau EE, Masalov DV, Tsoy MV, Burakov AM, Egorova VY, et al. Naltrexone with or without fluoxetine for preventing relapse to heroin addiction in St. Petersburg, Russia. Journal of substance abuse treatment. 2006;31(4):319-28. | Exclude: Reports treatment retention not discontinuation; Compliance monitored using riboflavin urine detection but no empirical data presented to allow calculable proportion of discontinuation. |
| 265. Kunoe N, Lobmaier P, Ngo H, Hulse G. Injectable and implantable sustained release naltrexone in the treatment of opioid addiction. British Journal of Clinical Pharmacology. 2014;77(2):264-71. | Exclude: Review article all references checked for inclusion |
| 266. Kunoe N, Lobmaier P, Vederhus JK, Hjerkinn B, Gossop M, Hegstad S, et al. Challenges to antagonist blockade during sustained-release naltrexone treatment. Addiction. 2010;105(9):1633-9. | Exclude: Study of report of testing blockade in naltrexone implants; Does not report calculable data on rate of naltrexone discontinuation or number with implant at end of study period |
| 267. Kunoe N, Lobmaier P, Vederhus JK, Hjerkinn B, Hegstad S, Gossop M, et al. Naltrexone implants after in-patient treatment for opioid dependence: randomised controlled trial. British Journal of Psychiatry. 2009;194(6):541-6. | Exclude: Discontinuation data reported in Kunoe 2010 pooled from three trials. Excluded to avoid double counting. Study level data extracted. |
| 268. Kunoe N, Lobmaier PP, Kornor H. Sustained-release naltrexone for opioid dependence: A cochrane review and meta-analysis. Drug and Alcohol Dependence. 2015;Conference: 2014 Annual Meeting of the College on Problems of Drug Dependence. San Juan Puerto Rico. 146:e165-e6. | Exclude: Conference abstract of systematic review of sustained release naltrexone. No initiation or discontinuation outcomes reported in abstract. Nil complete article subsequently published |
| 269. LaCasse M, Quigley J. Naltrexone for adolescent opioid use disorder: a bridge in the treatment gap? Journal of Substance Use. 2022;27(3):239-43. | Exclude: Review article all references checked for inclusion |
| 270. Ladewig D. Naltrexone--an effective aid in the psychosocial rehabilitation process of former opiate dependent patients. Therapeutische Umschau Revue Therapeutique. 1990;47(3):247-50. | Exclude: RCT; Does not report calculable discontinuation rates |
| 271. Landabaso M, Iraurgi I, Jimenez‐Lerma J, Sanz J, Corres BFD, Araluce K, et al. A randomized trial of adding fluoxetine to a naltrexone treatment programme for heroin addicts. Addiction. 1998;93(5):739-44. | Exclude: Reports retention in treatment programme; no empirical naltrexone discontinuation data |
| 272. Larochelle MR, Bernson D, Land T, Stopka TJ, Wang N, Xuan Z, et al. Medication for Opioid Use Disorder After Nonfatal Opioid Overdose and Association With Mortality: A Cohort Study. Annals of Internal Medicine. 2018;169(3):137-45. | Exclude: Does not report number of detoxified patients eligible and offered naltrexone who initiate. No discontinuation data reported |
| 273. Larochelle MR, Wakeman SE, Ameli O, Chaisson CE, McPheeters JT, Crown WH, et al. Relative Cost Differences of Initial Treatment Strategies for Newly Diagnosed Opioid Use Disorder: A Cohort Study. Medical Care. 2020;58(10):919-26. | Exclude: Reports number of people with OUD receiving naltrexone as their “initial treatment pathway” but unclear if all population eligible for naltrexone; i.e., currently opioid free - some individuals chose detoxification other partial hospitalisation, unclear of denominator eligible for naltrexone as a relapse prevention medication, No discontinuation data reported |
| 274. Lawental E. Ultra rapid opiate detoxification as compared to 30-day inpatient detoxification program - A retrospective follow-up study. Journal of Substance Abuse. 2000;11(2):173-81. | Exclude: Paper assessing long term follow up of withdrawal; All patients aimed to be inducted onto naltrexone following detoxification; No prevalence estimate of number of patients opting to initiate naltrexone calculable. Nil discontinuation of naltrexone data reported. |
| 275. Lee JD, Friedmann PD, Boney TY, Hoskinson RA, Jr., McDonald R, Gordon M, et al. Extended-release naltrexone to prevent relapse among opioid dependent, criminal justice system involved adults: rationale and design of a randomized controlled effectiveness trial. Contemporary Clinical Trials. 2015;41:110-7. | Exclude: Design and methods paper for a clinical trial |
| 276. Lee JD, McDonald R, Grossman E, McNeely J, Laska E, Rotrosen J, et al. Opioid treatment at release from jail using extended-release naltrexone: a pilot proof-of-concept randomized effectiveness trial. Addiction. 2015;110(6):1008-14. | Exclude: Clinical trial of XR-NTX. No empirical data reported to allow calculation of rate of initiation or discontinuation |
| 277. Lee JD, Nunes EV, Mpa PN, Bailey GL, Brigham GS, Cohen AJ, et al. NIDA Clinical Trials Network CTN-0051, Extended-Release Naltrexone vs. Buprenorphine for Opioid Treatment (X:BOT): Study design and rationale. Contemporary Clinical Trials. 2016;50:253-64. | Exclude: Design and methods paper of screened clinical trial |
| 278. Legarda JJ, Gossop M. A 24-h inpatient detoxification treatment for heroin addicts: A preliminary investigation. Drug and Alcohol Dependence. 1994;35(2):91-3. | Exclude: Paper assessing withdrawal; All patients aimed to be inducted onto naltrexone following detoxification; Nil other treatment options available and no prevalence estimate of number of patients opting to initiate naltrexone calculable. Nil discontinuation data reported. |
| 279. Lerner A, Sigal M, Bacalu A, Shiff R, Burganski I, Gelkopf M. A naltrexone double blind placebo controlled study in Israel. Isr J Psychiatry Relat Sci. 1992;29(1):36-43. | Exclude: Clinical trial reporting retention in rehabilitative treatment not naltrexone discontinuation. “retention in a rehabilitative program” |
| 280. Lerner AG, Gelkopf M, Sigal M, Oyffe I. Indicators of good prognosis in naltrexone treatment: A five-year prospective study. Addiction Research. 1997;4(4):385-91. | Exclude: Reports “Retention rate i.e., the rate at which patients were still in the program after 6 months. Patients were removed from the program and considered therapeutic failures when: 1-during the course of the study the patient failed to attend the established pattern of visits on at least 3 consecutive or 6 alternative times; 2-when at least one urine sample was found opiate positive, meaning that only opiate free patients remained in the program” Does not report data on naltrexone discontinuation. |
| 281. Leslie DL, Milchak W, Gastfriend DR, Herschman PL, Bixler EO, Velott DL, et al. Effects of injectable extended-release naltrexone (XR-NTX) for opioid dependence on residential rehabilitation outcomes and early follow-up. American Journal on Addictions. 2015;24(3):265-70. | Exclude: Unclear if those “recommended” for naltrexone treatment were eligible to receive it i.e., opioid free having completed self or medically assisted detoxification. No discontinuation data reported |
| 282. Li X, Langleben D, Lynch K, Wang GJ, Wiers CE, Shi Z. Body Mass Index as a Predictor of Extended-Release Naltrexone Treatment Retention in Individuals With Opioid Use Disorder. Neuropsychopharmacology. 2022;47(Supplement 1):416. | Exclude: Conference abstract reporting data are from individuals who participated in “XR-NTX treatment trials”; All XR-NTX treatment trials screened and original data included where appropriate excluded so as not to double count participants |
| 283. Lin A, McHugh RK, McCue B, Votaw V, Tester R, Rodolico J, et al. Acceptance of medication assisted treatment among uniformed service personnel receiving residential treatment for mental health and substance use disorders. American Journal on Addictions. 2017;26(3):285. | Exclude: Conference abstract; Unclear what relapse prevention medication assisted treatment participants were taking |
| 284. Lin LA, Powell VD, Macleod C, Bohnert ASB, Lagisetty P. Factors associated with clinician treatment recommendations for patients with a new diagnosis of opioid use disorder. Journal of Substance Abuse Treatment. 2022;141(no pagination). | Exclude: Reports number of people with OUD recommended for medications; Nil initiation or discontinuation data reported |
| 285. Ling W, Compton P. Recent advances in the treatment of opiate addiction. Clinical Neuroscience Research. 2005;5(2-4):161-7. | Exclude: Review article all references checked for inclusion |
| 286. Ling W, Mooney L, Wu L-T. Advances in opioid antagonist treatment for opioid addiction. Psychiatric Clinics of North America. 2012;35(2):297-308. | Exclude: Review article all references checked for inclusion |
| 287. Ling W, Rawson RA, Compton M. Clinical treatment of opioid addiction and dependence. Methods in molecular medicine. 2003;84:285-95. | Exclude: Review article all references checked |
| 288. Lintzeris N, Bell J, Bammer G, Jolley DJ, Rushworth L. A randomized controlled trial of buprenorphine in the management of short‐term ambulatory heroin withdrawal. Addiction. 2002;97(11):1395-404. | Exclude: Initiation data included from pooled results in Digustio et al. Discontinuation rate at one month not calculable from data presented. Reports 9 people in naltrexone at day 35 but not number commencing in each arm at day 8 post detoxification. |
| 289. Littlewood C. A new buprenorphine prescribing service for opiate detoxification. Psychiatric Bulletin. 2007;31(3):91-3. | Exclude: Study relating to withdrawal all patients inducted onto naltrexone; No empirical initation or discontinuation data reported |
| 290. Lobmaier P, Gossop M, Waal H, Bramness J. The pharmacological treatment of opioid addiction--a clinical perspective. European Journal of Clinical Pharmacology. 2010;66(6):537-45. | Exclude: Review article all references checked for inclusion |
| 291. Lobmaier P, Kornor H, Kunoe N, Bjorndal A. Sustained-release naltrexone for opioid dependence. Cochrane Database of Systematic Reviews. 2008;(2) (no pagination). | Exclude: Review article all references checked for inclusion |
| 292. Lobmaier PP, Kunoe N, Gossop M, Katevoll T, Waal H. Naltrexone implants compared to methadone: outcomes six months after prison release. European Addiction Research. 2010;16(3):139-45. | Exclude: Clinical trial for which discontinuation data is reported in pooled analysis Kunoe 2010. Excluded to avoid double counting. Individual data extracted for trial characteristics. |
| 293. Lobmaier PP, Kunøe N, Waal H. Treatment research in prison: Problems and solutions in a randomized trial. Addiction Research & Theory. 2010;18(1):1-13. | Exclude: Duplicate data and trial of Lobemaier 2010, discontinuation data from which is included in Kunoe 2010. Excluded to avoid double counting |
| 294. Loffreda A, Falcone G, Motola G, Mazzeo F, Iacobelli M, Ferrari P, et al. Use of naltrexone for the treatment of opiate addiction in Campania, Italy: The role of family. Journal of Substance Use. 2003;8(3):182-5. | Exclude: Only potential discontinuation data reported is that “Twenty per cent of patients withdrew from the trial in the very first months” Unclear if timepoint is one month, unclear if withdrawal from trial is discontinuation of naltrexone therapy. |
| 295. Loimer N, Lenz K, Schmid R, Presslich O. Technique for greatly shortening the transition from methadone to naltrexone maintenance of patients addicted to opiates. American Journal of Psychiatry. 1991;148(7):933-5. | Exclude: Paper assessing induction onto naltrexone; All patients aimed to be inducted onto naltrexone following detoxification; Nil other treatment options available and no prevalence estimate of number of patients opting to initiate naltrexone calculable. Nil discontinuation data reported. |
| 296. London M, Paul E, Gkolia I. Ultra-rapid opiate detoxification in hospital. Psychiatric Bulletin. 1999;23(9):544-6. | Exclude: Paper assessing withdrawal strategies; All patients aimed to be inducted onto naltrexone following detoxification; Nil other treatment options available and no prevalence estimate of number of patients opting to initiate naltrexone calculable. Nil discontinuation of naltrexone specific data reported. |
| 297. Lorenzi P, Marsili M, Boncinelli S, Fabbri LP, Fontanari P, Zorn AM, et al. Searching for a general anaesthesia protocol for rapid detoxification from opioids. European Journal of Anaesthesiology. 1999;16(10):719-27. | Exclude: Paper assessing withdrawal; All patients aimed to be inducted onto naltrexone following detoxification; No prevalence estimate of number of patients opting to initiate naltrexone calculable. Nil discontinuation of naltrexone data reported. |
| 298. Lundy C, Stringer M, Zummo J, Crits-Christoph P, Gastfriend DR. Extended-release naltrexone (XR-NTX) in St. Louis parolees and probationers. Alcoholism: Clinical and Experimental Research. 2014;1):213A. | Exclude: Conference abstract of reviewed article Crits-Christoph 2015 reporting retention in both alcohol and opioid dependence. No discontinuation rates reported. |
| 299. Luty J. Treatment preferences of opiate-dependent patients. Psychiatric Bulletin. 2004;28(2):47-50. | Exclude: Patient preference survey of treatments; Nil data of initiation/discontinuation presented |
| 300. Macedo TR, Relvas J, Fontes Ribeiro CA, Pacheco F, Morgadinho MT, Pinto CM, et al. Plasma catecholamines during an ultrarapid heroin detoxification. Annals of the New York Academy of Sciences. 2000;914:303-10. | Exclude: Paper assessing withdrawal strategies; All patients aimed to be inducted onto naltrexone following detoxification; Nil other treatment options available and no prevalence estimate of number of patients opting to initiate naltrexone calculable. Nil discontinuation data reported. |
| 301. Mannelli P, Andrew Tompkins D, Akerman S, Yu M, Nangia N, Graham C, et al. Transition to extended-release naltrexone: Heroin versus prescription opioid users. American Journal on Addictions. 2018;27(4):323-4. | Exclude: No data reported to enable calculable rate of discontinuation; Planned all initiation cohort onto NTX-XR |
| 302. Mannelli P, Douaihy AB, Akerman SC, Legedza A, Fratantonio J, Zavod A, et al. Characteristics and treatment preferences of individuals with opioid use disorder seeking to transition from buprenorphine to extended-release naltrexone in a residential setting. American Journal on Addictions. 2022;31(2):142-7. | Exclude: Cross sectional survey of reasons for switching from buprenorphine to naltrexone |
| 303. Mannelli P, Douaihy AB, Zavod A, Legedza A, Akerman SC, Sullivan MA. Patterns of withdrawal in patients with opioid use disorder (OUD) transitioning from untreated OUD or buprenorphine treatment to extended-release naltrexone. Am J Drug Alcohol Abuse. 2021;47(6):753-9. | Exclude: Post hoc analyses of two screened clinical trials assessing withdrawal symptomatology |
| 304. Mannelli P, Gottheil E, Buonanno A, De Risio S. Use of very low-dose naltrexone during opiate detoxification. Journal of Addictive Diseases. 2003;22(2):63-70. | Exclude: Reports 5 patients inducted onto naltrexone following detoxification and states “The 5 patients were still heroin-free and taking naltrexone 50 mg/day two weeks post discharge” unclear one month or other discontinuation rates. |
| 305. Mannelli P, Gottheil E, Van Bockstaele EJ. Antagonist treatment of opioid withdrawal translational low dose approach. Journal of Addictive Diseases. 2006;25(2):1-8. | Exclude: Review article all references checked for inclusion |
| 306. Mannelli P, Patkar AA, Peindl K, Gorelick DA, Wu LT, Gottheil E. Very low dose naltrexone addition in opioid detoxification: a randomized, controlled trial. Addiction Biology. 2009;14(2):204-13. | Exclude: Reports trial for withdrawal strategies with naltrexone; No empirical data reported to for calculable naltrexone initiation or discontinuation. |
| 307. Mannelli P, Patkar AA, Peindl K, Gottheil E, Wu LT, Gorelick DA. Early outcomes following low dose naltrexone enhancement of opioid detoxification. American Journal on Addictions. 2009;18(2):109-16. | Exclude: Reports retention in treatment at one week post detoxification; No empirical data reported to for calculable naltrexone initiation or discontinuation. |
| 308. Mannelli P, Peindl K, Patkar AA, Wu L-T, Pae C-U, Gorelick DA. Reduced cannabis use after low-dose naltrexone addition to opioid detoxification. Journal of Clinical Psychopharmacology. 2010;30(4):476-8. | Exclude: Study on withdrawal using naltrexone. Does not report a rate of naltrexone discontinuation |
| 309. Mannelli P, Peindl K, Wu LT. Predictors of induction onto XR-NTX in patients with opioid use disorder. Drug and Alcohol Dependence. 2015;Conference: 2015 Annual Meeting of the College on Problems of Drug Dependence, CPDD 2015. Phoenix, AZ United States. 156:e138-e9. | Exclude: Conference abstract describing success of induction onto XR-NTR not numbers of patient opting to initiate naltrexone |
| 310. Mannelli P, Peindl K, Wu LT, Patkar AA, Gorelick DA. The combination very low-dose naltrexone-clonidine in the management of opioid withdrawal. Am J Drug Alcohol Abuse. 2012;38(3):200-5. | Exclude: Reports study of withdrawal. Does not report calculable rates of naltrexone discontinuation |
| 311. Mannelli P, Peindl KS, Lee T, Bhatia KS, Wu LT. Buprenorphine-mediated transition from opioid agonist to antagonist treatment: state of the art and new perspectives. Current Drug Abuse Reviews. 2012;5(1):52-63. | Exclude: Review article of switching strategies from methadone to alternative medications for opioid use disorder, references checked for inclusion |
| 312. Mannelli P, Peindl KS, Wu LT. Feasibility of very low dose naltrexone and buprenorphine transition from opioid use to extended-release naltrexone. Drug and Alcohol Dependence. 2015;Conference: 2014 Annual Meeting of the College on Problems of Drug Dependence. San Juan Puerto Rico. 146:e192. | Exclude: Conference abstract of transition strategies onto XR-NTX not calculable numbers opting to initiate naltrexone. |
| 313. Mannelli P, Swartz M, Wu LT. Withdrawal severity and early response to treatment in the outpatient transition from opioid use to extended release naltrexone. American Journal on Addictions. 2018;27(6):471-6. | Exclude: Reports prevalence of successful induction onto naltrexone following detoxification in a cohort having preselected this treatment regimen; Nil discontinuation data reported |
| 314. Mannelli P, Wu LT, Peindl KS, Swartz MS, Woody GE. Extended release naltrexone injection is performed in the majority of opioid dependent patients receiving outpatient induction: a very low dose naltrexone and buprenorphine open label trial. Drug Alcohol Depend. 2014;138:83-8. | Exclude: Study of withdrawal and induction onto NTX-XR reports number of follow-up visits attended. Nil discontinuation data reported. |
| 315. Marciuch A, Brenna IH, Weimand B, Solli KK, Tanum L, Rostad BK, et al. Patients' experiences of continued treatment with extended-release naltrexone: a Norwegian qualitative study. Addict Sci Clin Pract. 2022;17(1):36. | Exclude: Qualitative Study; No empirical data on number of people initiating or discontinuing naltrexone |
| 316. Marcus R, Bojko MJ, Mazhnaya A, Makarenko I, Filippovych S, Dvoriak S, et al. A qualitative assessment of attitudes about and preferences for extended-release naltrexone, a new pharmacotherapy to treat opioid use disorders in Ukraine. Journal of Substance Abuse Treatment. 2018;86:86-93. | Exclude: Qualitative study: No empirical data on numbers of people initiating or discontinuing naltrexone |
| 317. Marcus R, Makarenko I, Mazhnaya A, Zelenev A, Polonsky M, Madden L, et al. Patient preferences and extended-release naltrexone: A new opportunity to treat opioid use disorders in Ukraine. Drug Alcohol Depend. 2017;179:213-9. | Exclude: Cross sectional survey of acceptability of XR-NTX |
| 318. Mariani JJ, Basaraba C, Pavlicova M, Alschuler DM, Brooks DJ, Mahony AL, et al. Open label trial of lofexidine-assisted non-opioid induction onto naltrexone extended-release injection for opioid use disorder. Am J Drug Alcohol Abuse. 2023;49(5):618-29. | Exclude: Evaluates induction protocol of lofexidine and numbers inducted onto XR-NTX; Nil opting for initation data or discontinuation naltrexone data reported |
| 319. Martinez-Raga J, Sabater A, Perez-Galvez B, Castellano M, Cervera G. Add-on gabapentin in the treatment of opiate withdrawal. Progress in Neuro-Psychopharmacology & Biological Psychiatry. 2004;28(3):599-601. | Exclude: Paper assessing withdrawal; All patients aimed to be inducted onto naltrexone following detoxification; No prevalence estimate of number of patients opting to initiate naltrexone calculable. Nil discontinuation of naltrexone data reported |
| 320. Mattick R, Bell J, Daws L, White J, OBrien S, Harris S. Review of the evidence on the effectiveness of antagonists in managing opioid dependence. NDARC monograph. 1998;34. | Exclude: Review article all references checked |
| 321. Mattick RP, Hall W. Are detoxification programmes effective? Lancet. 1996;347(8994):97-100. | Exclude: Review article all references checked |
| 322. Mburu M, Masese R, Knippler ET, Watt MH, Muhirwa A, Ledbetter L, et al. Factors associated with medications for opioid use disorder (MOUD) treatment success during the pregnancy and postpartum periods: A scoping review. Drug Alcohol Depend. 2024;264:112454. | Exclude: Review article all references checked for inclusion |
| 323. McCann MJ, Miotto K, Rawson RA, Huber A, Shoptaw S, Ling W. Outpatient non-opioid detoxification for opioid withdrawal. Who is likely to benefit? American Journal on Addictions. 1997;6(3):218-23. | Exclude: Paper assessing opioid withdrawal; All patients aimed to be inducted onto naltrexone following detoxification; Nil other treatment options available and no prevalence estimate of number of patients opting to initiate naltrexone calculable. Nil discontinuation data reported. |
| 324. McCausland K, Haider B, White M, O'Sullivan A, Kosinski M, Akerman S, et al. Current practice in withdrawal management: opportunities to improve treatment of opioid use disorder. American Journal on Addictions. 2020;29(3):202-3. | Exclude: HCP survey for factors associated with XR-NTX initiation |
| 325. McDonald RD, Tofighi B, Laska E, Goldfeld K, Bonilla W, Flannery M, et al. Extended-release naltrexone opioid treatment at jail reentry (XOR). Contemporary Clinical Trials. 2016;49:57-64. | Exclude: Design and methods of screened clinical trial |
| 326. McGregor C, Ali R, White JM, Thomas P, Gowing L. A comparison of antagonist-precipitated withdrawal under anesthesia to standard inpatient withdrawal as a precursor to maintenance naltrexone treatment in heroin users: Outcomes at 6 and 12 months. Drug and Alcohol Dependence. 2002;68(1):5-14. | Exclude: Pooled induction data included via the review Digustio et al, which captures additional unpublished data. Reports retention by attendance not naltrexone discontinuation data “retention between the groups for the first 3 months (defined as collecting naltrexone tablets and attending medical and research interviews)” |
| 327. McKay JR. Continuing care in the treatment of addictive disorders. Current Psychiatry Reports. 2006;8(5):355-62. | Exclude: Review article all references checked |
| 328. Mello NK, Mendelson JH, Kuehnle JC, Sellers MS. Operant analysis of human heroin self-administration and the effects of naltrexone. Journal of Pharmacology and Experimental Therapeutics. 1981;216(1):45-54. | Exclude: RCT of naltrexone vs placebo; Nil discontinuation data reported |
| 329. Merlo LJ, Greene WM, Pomm R. Mandatory naltrexone treatment prevents relapse among opiate-dependent anesthesiologists returning to practice. Journal of Addiction Medicine. 2011;5(4):279-83. | Exclude: Reports rates of relapse to use; does not reports rates of naltrexone discontinuation in the group using naltrexone |
| 330. Messinis L, Lyros E, Andrian V, Katsakiori P, Panagis G, Georgiou V, et al. Neuropsychological functioning in buprenorphine maintained patients versus abstinent heroin abusers on naltrexone hydrochloride therapy. Human Psychopharmacology. 2009;24(7):524-31. | Exclude; No empirical data reported to for calculable naltrexone initiation or discontinuation. |
| 331. Metzger DS, Cornish J, Woody GE, McLellan AT, Druley P, O'Brien CP. Naltrexone in federal probationers. NIDA Research Monograph Series. 1989(95):465-6. | Exclude: Abstract reporting preliminary results of clinical trial in research monograph. Does not report number of participants in naltrexone group and reports overall retention in treatment rate not rate of naltrexone discontinuation. |
| 332. Meyer R, Gastfriend D, Milchak W, Bixler E, Leslie D, Bird J, et al. Injectable extended-release naltrexone in opioid dependence: Patients characteristics, coverage and hospital course. Journal of Addiction Medicine. 2013;7(4):E6. | Exclude: Does not report denominator of patients eligible (i.e., opioid free post self or medically assisted opioid withdrawal) and offered naltrexone only those in full cohort of people with OUD “recommended for naltrexone”. Does not repot discontinuation data. |
| 333. Minovitz O. Drug treatment of opioid dependence. Canadian Medical Association Journal. 1988;138(6):496. | Exclude: Letter; Nil empirical data reported |
| 334. Minozzi S, Amato L, Bellisario C, Davoli M. Detoxification treatments for opiate dependent adolescents. Cochrane Database of Systematic Reviews. 2014(4):CD006749. | Exclude: Does not report calculable initiation or discontinuation rates for naltrexone. |
| 335. Minozzi S, Amato L, Davoli M. Detoxification treatments for opiate dependent adolescents. Cochrane Database of Systematic Reviews. 2009(2):CD006749. | Exclude: Updated Cochrane Review available |
| 336. Minozzi S, Amato L, Vecchi S, Davoli M, Kirchmayer U, Verster A. Oral naltrexone maintenance treatment for opioid dependence. Cochrane Database of Systematic Reviews. 2006(1):CD001333. | Exclude: Updated Cochrane Review Available |
| 337. Minozzi S, Amato L, Vecchi S, Davoli M, Kirchmayer U, Verster A. Oral naltrexone maintenance treatment for opioid dependence. Cochrane Database of Systematic Reviews. 2011(2):CD001333. | Exclude: Review article all references checked for inclusion |
| 338. Mintz CM, Presnall NJ, Sahrmann JM, Borodovsky JT, Glaser PE, Bierut LJ, et al. Age disparities in six-month treatment retention for opioid use disorder. Drug and alcohol dependence. 2020;213:108130. | Exclude: Reports retention in treatment; Nil initiation or discontinuation data for naltrexone reported. |
| 339. Miotto K, McCann MJ, Rawson RA, Frosch D, Ling W. Overdose, suicide attempts and death among a cohort of naltrexone-treated opioid addicts. Drug and alcohol dependence. 1997;45(1-2):131-4. | Exclude: Reports discontinuation rates at two weeks only for those participants who experienced adverse events on naltrexone. With respect to initiation reports “”Out of 167 who initiated outpatient detoxification treatment, 65 were successfully inducted onto naltrexone” does not report denominator for those successfully completing detoxification and eligible for naltrexone. |
| 340. Mitchell MM, Schwartz RP, Choo TH, Pavlicova M, O'Grady KE, Gryczynski J, et al. An alternative analysis of illicit opioid use during treatment in a randomized trial of extended-release naltrexone versus buprenorphine-naloxone: A per-protocol and completers analysis. Drug Alcohol Depend. 2021;219:108422. | Exclude: Post hoc analysis of screened clinical trial examining per protocol population for opioid relapse outcomes |
| 341. Mogali S, Khan NA, Drill ES, Pavlicova M, Sullivan MA, Nunes E, et al. Baseline characteristics of patients predicting suitability for rapid naltrexone induction. American Journal on Addictions. 2015;24(3):258-64. | Exclude: Reports predictor of successful initiation of naltrexone after a rapid induction-detoxification, not number of patients opting to initiate naltrexone. |
| 342. Mokri A, Chawarski MC, Taherinakhost H, Schottenfeld RS. Medical treatments for opioid use disorder in Iran: a randomized, double-blind placebo-controlled comparison of buprenorphine/naloxone and naltrexone maintenance treatment. Addiction. 2016;111(5):874-82. | Exclude: Reports retention; Does not report data to enable calculation of rate of initiation or discontinuation of naltrexone |
| 343. Moore KE, Roberts W, Reid HH, Smith KMZ, Oberleitner LMS, McKee SA. Effectiveness of medication assisted treatment for opioid use in prison and jail settings: A meta-analysis and systematic review. Journal of Substance Abuse Treatment. 2019;99:32-43. | Exclude: Review article all references checked for inclusion |
| 344. Morse E, Binswanger IA, Taylor E, Gray C, Stimmel M, Timko C, et al. Strategies to improve implementation of medications for opioid use disorder reported by veterans involved in the legal system: A qualitative study. Journal of Substance Abuse Treatment. 2021;129(no pagination). | Exclude: Qualitative Study; No empirical data on number of people initiating or discontinuing naltrexone |
| 345. Murphy PN, Mohammed F, Wareing M, Cotton A, McNeill J, Irving P, et al. High drug related mortality rates following prison release: Assessing the acceptance likelihood of a naltrexone injection and related concerns. Journal of Substance Abuse Treatment. 2018;92:91-8. | Exclude: Cross sectional survey of likelihood of accepting naltrexone on release from prison |
| 346. Murphy S, McCollister KE, Jeng P, Leff J, Lee JD, Nunes EV, et al. Pdg72 Determinants of an Efficient Model of Extended-Release Naltrexone Initiation for the Treatment of Opioid Use Disorder. Value in Health. 2019;22(Supplement 2):S175. | Exclude: Conference Abstract of screened clinical trial; No empirical data in abstract reported on the prevalence of naltrexone initiation/discontinuation |
| 347. Murphy SM, Jeng PJ, McCollister KE, Leff JA, Jalali A, Shulman M, et al. Cost-effectiveness implications of increasing the efficiency of the extended-release naltrexone induction process for the treatment of opioid use disorder: a secondary analysis. Addiction. 2021;116(12):3444-53. | Exclude: Modelling study of naltrexone induction regimens. |
| 348. Murphy SM, McCollister KE, Leff JA, Yang X, Jeng PJ, Lee JD, et al. Cost-Effectiveness of Buprenorphine-Naloxone Versus Extended-Release Naltrexone to Prevent Opioid Relapse. Annals of Internal Medicine. 2019;170(2):90-8. | Exclude: Cost-effectiveness analysis of screened clinical trial. |
| 349. Murphy SM, Polsky D, Lee JD, Friedmann PD, Kinlock TW, Nunes EV, et al. Cost-effectiveness of extended release naltrexone to prevent relapse among criminal justice-involved individuals with a history of opioid use disorder. Addiction. 2017;112(8):1440-50. | Exclude: Cost effectiveness evaluation of screened clinical trial Lee et al. 2016 |
| 350. Muthulingam D, Hassett TC, Madden LM, Bromberg DJ, Fraenkel L, Altice FL. Preferences in medications for patients seeking treatment for opioid use disorder: A conjoint analysis. Journal of Substance Use and Addiction Treatment. 2023;154:209138. | Exclude: Cross sectional survey of attitudes to medications for opioid use disorder |
| 351. Mysels DJ, Cheng WY, Nunes EV, Sullivan MA. The association between naltrexone treatment and symptoms of depression in opioid-dependent patients. Am J Drug Alcohol Abuse. 2011;37(1):22-6. | Exclude: Reports association of depressive symptoms among people maintained on naltrexone. Does not report calculable rates of naltrexone discontinuation |
| 352. Naderi-Heiden A, Naderi A, Naderi MM, Rahmani-Didar F, Salimi AR, Gleiss A, et al. Ultra-rapid opiate detoxification followed by nine months of naltrexone maintenance therapy in Iran. Pharmacopsychiatry. 2010;43(4):130-7. | Exclude: Reports naltrexone discontinuation rates at 9 months post initiation |
| 353. Nasr DAM, Omran HASA, Hakim SM, Mansour WAA. Ultra-rapid opiate detoxification using dexmedetomidine under general anesthesia. Journal of Opioid Management. 2011;7(5):337-44. | Exclude: Reports on withdrawal strategies; Does not report calculable rates of naltrexone discontinuation |
| 354. Nasseri K, Ahsan B, Farhadifar F, Shami S. Shortening anesthesia duration does not affect severity of withdrawal syndrome in patients undergoing ultra rapid opioid detoxification. Acta Med Iran. 2010;48(1):27-32. | Exclude: Study of withdrawal; Nil calculable rates of naltrexone discontinuation reported |
| 355. Navaratnam V, Jamaludin A, Raman N, Mohamed M, Mansor SM. Determination of naltrexone dosage for narcotic agonist blockade in detoxified Asian addicts. Drug Alcohol Depend. 1994;34(3):231-6. | Exclude: Dose finding study; Nil empirical data on initiation/discontinuation reported |
| 356. Ngo H, Arnold-Reed D, Hansson RC, Tait RJ, Hulse GK. Blood naltrexone levels over time following naltrexone implant. Progress in Neuro-Psychopharmacology and Biological Psychiatry. 2008;32(1):23-8. | Exclude: Does not report discontinuation data of individuals receiving number of implants, only first implant received. No calculable discontinuation rates |
| 357. Ngo HT, Tait RJ, Hulse GK. Comparing drug-related hospital morbidity following heroin dependence treatment with methadone maintenance or naltrexone implantation. Archives of General Psychiatry. 2008;65(4):457-65. | Exclude: Does not report discontinuation data of individuals receiving number of implants, only first implant received. Describes three cases implant removed in the first week but not in the analyses cohort and does not report any further implant removals. No calculable discontinuation rates |
| 358. Nishio M, Gevirtz CM. Rapid opioid detoxification under general anesthesia [2]. Journal of Anesthesia. 1997;11(4):315. | Exclude: Description of rapid opioid detoxification under general anaesthesia |
| 359. Nunes E, Bisaga A, Krupitsky E, Nangia N, Silverman B, Akerman S, et al. Effects of extended-release naltrexone extend beyond exogenous opioid blockade: Clinical trial observations. American Journal on Addictions. 2018;27(4):322-3. | Exclude: Conference abstract of screened clinical trial reporting on dropout from treatment, no calculable rate of initiation or discontinuation reported |
| 360. Nunes EV, Bisaga A, Krupitsky E, Nangia N, Silverman BL, Akerman SC, et al. Opioid use and dropout from extended-release naltrexone in a controlled trial: implications for mechanism. Addiction. 2020;115(2):239-46. | Exclude: Secondary analysis of screened clinical trail. Reports “drop out” defined buy non attendance no discontinuation data presented to allow calculable rate |
| 361. Nunes EV, Gordon M, Friedmann PD, Fishman MJ, Lee JD, Chen DT, et al. Relapse to opioid use disorder after inpatient treatment: Protective effect of injection naltrexone. Journal of Substance Abuse Treatment. 2018;85:49-55. | Exclude: Secondary analysis of screened trial reporting on relapse outcomes |
| 362. Nunes EV, Krupitsky E, Ling W, Zummo J, Memisoglu A, Silverman BL, et al. Treating Opioid Dependence With Injectable Extended-Release Naltrexone (XR-NTX): Who Will Respond? Journal of Addiction Medicine. 2015;9(3):238-43. | Exclude: Secondary analysis of screened clinical trial looking at predictors of abstinence. |
| 363. Nunes EV, Rothenberg JL, Sullivan MA, Carpenter KM, Kleber HD. Behavioral therapy to augment oral naltrexone for opioid dependence: a ceiling on effectiveness? Am J Drug Alcohol Abuse. 2006;32(4):503-17. | Exclude: Reports retention in treatment defined by clinic attendance “time to dropout from treatment or 26 weeks for those who completed treatment. Patients who relapsed or did not attend the clinic at least once within a 14-day period were rated as dropouts” and percentage compliance but does not report naltrexone discontinuation rates. |
| 364. Nunes EV, Jr., Scodes JM, Pavlicova M, Lee JD, Novo P, Campbell ANC, et al. Sublingual Buprenorphine-Naloxone Compared With Injection Naltrexone for Opioid Use Disorder: Potential Utility of Patient Characteristics in Guiding Choice of Treatment. American Journal of Psychiatry. 2021;178(7):660-71. | Exclude: Secondary analysis of screened clinical trial examining patient characteristics. |
| 365. O'Brien C, Cornish JW. Naltrexone for probationers and parolees. Journal of Substance Abuse Treatment. 2006;31(2):107-11. | Exclude: Review article all references checked |
| 366. O'Brien CP, Friedmann PD, Nunes E, Lee JD, Kinlock TW. Depot naltrexone as relapse prevention for opioid-dependent parolees. Drug and Alcohol Dependence. 2015;Conference: 2014 Annual Meeting of the College on Problems of Drug Dependence. San Juan Puerto Rico. 146:e54-e5. | Exclude: Conference abstract of clinical trial reporting retention. No empirical data enabling calculation of rate of initiation or discontinuation reported. |
| 367. O'Brien CP, Greenstein R, Woody GE. Update of naltrexone treatment. NIDA Res Monogr. 1978(19):315-20. | Exclude: No empirical data presented and descriptions are an update on the included Greenstein 1976. No data reported initiation or discontinuation prevalence. |
| 368. O'Brien CP, Greenstein RA, Evans B, Woody GE, Arndt R. Opioid antagonists: do they have a role in treatment programs? NIDA Res Monogr. 1983;43:71-8. | Exclude: Review article; All references checked |
| 369. O'brien CP, Greenstein RA, Mintz J, Woody GE. Clinical experience with naltrexone. The American journal of drug and alcohol abuse. 1975;2(3-4):365-77. | Exclude: Earlier report of Greenstein 1976 which includes more patients. Exclude due to avoid double counting |
| 370. O'Brien CP, Terenius L, Wahlstrom A, McLellan AT, Krivoy W. Endorphin levels in opioid-dependent human subjects: a longitudinal study. Annals of the New York Academy of Sciences. 1982;398:377-87. | Exclude: No empirical data reported on the prevalence of or the factors associated with naltrexone initiation/discontinuation |
| 371. O'Connor PG, Carroll KM, Shi JM, Schottenfeld RS, Kosten TR, Rounsaville BJ. Three methods of opioid detoxification in a primary care setting. A randomized trial. Annals of Internal Medicine. 1997;127(7):526-30. | Exclude: Paper assessing withdrawal and induction onto naltrexone; All patients aimed to be inducted onto naltrexone following detoxification; No prevalence estimate of number of patients opting to initiate naltrexone calculable. Nil discontinuation of naltrexone data reported. |
| 372. O'Connor PG, Kosten TR. Rapid and ultrarapid opioid detoxification techniques. Jama. 1998;279(3):229-34. | Exclude: Review of rapid detoxication: All references checked |
| 373. O'Connor PG, Waugh ME, Carroll KM, Rounsaville BJ, Diagkogiannis IA, Schottenfeld RS. Primary care-based ambulatory opioid detoxification: the results of a clinical trial. Journal of General Internal Medicine. 1995;10(5):255-60. | Exclude: Paper assessing withdrawal regimens; All patients aimed to be inducted onto naltrexone following detoxification; Nil other treatment options available and no prevalence estimate of number of patients opting to initiate naltrexone calculable. Nil discontinuation data reported. |
| 374. O'Connor PG, Waugh ME, Schottenfeld RS, Diakogiannis IA, Rounsaville BJ. Ambulatory opiate detoxification and primary care: a role for the primary care physician. Journal of General Internal Medicine. 1992;7(5):532-4. | Exclude: Does not report the number of participants successfully completing detoxification, eligible and initiating naltrexone. Reports “patients were referred for relapse prevention to a nearby outpatient program for naltrexone maintenance and counselling” Does not state number of patients that received naltrexone. Discontinuation not reported only “Successful initiation of relapse prevention was defined as participation in treatment for one month.” |
| 375. O'Malley SS, Froehlich JC. Advances in the use of naltrexone: An integration of preclinical and clinical findings.  Recent developments in alcoholism, Volume 16: Research on alcoholism treatment. New York, NY: Kluwer Academic/Plenum Publishers; US; 2003. p. 217-45. | Exclude: Textbook review of naltrexone; All references checked |
| 376. O'Neil G, Hulse G, Armstrong J, Little M, Murray L. Rapid opioid detoxification in Australia. Academic emergency medicine : official journal of the Society for Academic Emergency Medicine. 2002;9(9):960. | Exclude: Editorial all references checked |
| 377. O'Shea J, Law F, Melichar J. Opioid dependence. Clin Evid (Online). 2009;24:24. | Exclude: Review article all references checked for inclusion |
| 378. Ockert DM, Volpicelli JR, Baier AR, Jr., Coons EE, Fingesten A. A nonopioid procedure for outpatient opioid detoxification. Journal of Addiction Medicine. 2011;5(2):110-4. | Exclude: Reports withdrawal strategies. Does not report calculable rates of naltrexone discontinuation |
| 379. Opheim A, Benth JS, Solli KK, Kloster PS, Fadnes LT, Kunoe N, et al. Risk of relapse to non-opioid addictive substances among opioid dependent patients treated with an opioid receptor antagonist or a partial agonist: A randomized clinical trial. Contemporary Clinical Trials. 2023;135:107360. | Exclude: Secondary analysis of screened trial reporting other substance relapse outcomes |
| 380. Opheim A, Gaulen Z, Solli KK, Latif ZE, Fadnes LT, Benth JS, et al. Risk of Relapse Among Opioid-Dependent Patients Treated With Extended-Release Naltrexone or Buprenorphine-Naloxone: A Randomized Clinical Trial. American Journal on Addictions. 2021;30(5):453-60. | Exclude: Secondary analysis of screened clinical trial examining relapse outcomes |
| 381. Opheim A, Haase KS, Tanum L, Kunoe N, Nesvag S, Stavseth L, et al. Existential anxiety in first-time recipients of XR-NTX for opioid addiction. Drug and Alcohol Dependence. 2015;Conference: 2014 Annual Meeting of the College on Problems of Drug Dependence. San Juan Puerto Rico. 146:e58. | Exclude: Conference abstract of anxiety symptoms during first XR-NTX injection |
| 382. Osborn E, Grey C, Reznikoff M. Psychosocial adjustment, modality choice, and outcome in naltrexone versus methadone treatment. The American journal of drug and alcohol abuse. 1986;12(4):383-8. | Exclude: Reports retention as measured by clinic attendance and not naltrexone discontinuation “retention was calculated by the number of required clinic appointments kept during the 12-week period of the study” |
| 383. Otiashvili D, Kirtadze I, O'Grady KE, Jones HE. Drug use and HIV risk outcomes in opioid-injecting men in the Republic of Georgia: behavioral treatment + naltrexone compared to usual care. Drug Alcohol Depend. 2012;120(1-3):14-21. | Exclude: Does not report calculable rates of naltrexone discontinuation |
| 384. Palatkin V, Kibitov A, Krupitsky E, Blokhina E, Brodyansky V, Verbitskaya E, et al. Role of dopamine and opioid gene polymorphisms in efficacy of naltrexone and guanfacine combination for relapse prevention among opioid addicts. European Neuropsychopharmacology. 2017;27(Supplement 4):S1069. | Exclude: Conference abstract of screened clinical trial reporting genetic outcomes |
| 385. Palatkin V, Krupitsky E, Zvartau E, Blokhina E, Bushara N, Tyurina A, et al. Effectiveness of naltrexone in the double blind placebo controlled clinical trial of naltrexone and guanfacine for heroin dependence. European Neuropsychopharmacology. 2012;1):S61-S2. | Exclude: Conference abstract of clinical trial: Abstract reports retention. Does not report calculable rates of naltrexone discontinuation |
| 386. Palatkin V, Krupitsky E, Zvartau E, Blokhina E, Bushara N, Tyurina A, et al. Stress-protective and anticraving effects of guanfacine in opiate dependent patients stabilized on oral naltrexone. European Neuropsychopharmacology. 2014;2):S688. | Exclude: Conference abstract of screened clinical trial reporting on stress outcomes. |
| 387. Peglow SL, Moran J, Keats M. Antagonist treatment for opioid use disorder-Development of antagonist treatment: Antagonists versus agonist treatments pros and cons.  Substance use disorders. New York, NY: Oxford University Press; US; 2021. p. 169-84. | Exclude: Book chapter describing naltrexone use all references checked for inclusion. |
| 388. Pereska Z, Dejanova B, Bozinovska C, Petkovska L. Prooxidative/antioxidative homeostasis in heroin addiction and detoxification. Bratisl Lek Listy. 2007;108(9):393-8. | Exclude: No empirical initation or discontinuation data reported |
| 389. Perez-Macia V, Martinez-Cortes M, Mesones J, Segura-Trepichio M, Garcia-Fernandez L. Monitoring and improving naltrexone adherence in patients with substance use disorder. Patient Preference and Adherence. 2021;15:999-1015. | Exclude: Systematic review all references checked for inclusion |
| 390. Pfab R, Hirtl C, Zilker T. Opiate detoxification under anesthesia: No apparent benefit but suppression of thyroid hormones and risk of pulmonary and renal failure. Journal of Toxicology - Clinical Toxicology. 1999;37(1):43-50. | Exclude: Paper assessing withdrawal strategies; All patients aimed to be inducted onto naltrexone following detoxification; Nil other treatment options available and no prevalence estimate of number of patients opting to initiate naltrexone calculable. Nil discontinuation data reported. |
| 391. Pfaff A, Cochran A, Vechinski J, Molfenter T, Zayas-Caban G. The association between attitudes and the provision of medications for opioid use disorder (MOUD) in United States jails. Drug and Alcohol Dependence Reports. 2024;10(no pagination). | Exclude: Cross sectional survey of providers and jails of attitudes towards medications for opioid use disorder |
| 392. Pilarinos A, Bromberg DJ, Karamouzian M. Access to Medications for Opioid Use Disorder and Associated Factors Among Adolescents and Young Adults: A Systematic Review. JAMA Pediatrics. 2022;176(3):304-11. | Exclude: Systematic review all references checked for inclusion |
| 393. PLUS VN. COMPARISON OF TWO NALTREXONE TREATMENT. NALTREXONE. 1976:150. | Exclude: Reports average length of time naltrexone taken per group not calculable numbers of people discontinuing. |
| 394. Pourtaher E, Gelberg KH, Fallico M, Ellendon N, Li S. Expanding access to Medication for Opioid Use Disorder (MOUD) in jails: A comprehensive program evaluation. Journal of Substance Use and Addiction Treatment. 2024;161:209248. | Exclude: Reports number of people initiating medication for opioid use disorder in jails; Unclear number eligible for naltrexone; No discontinuation/continuation data presented for naltrexone |
| 395. Pozzi G, Conte G, De Risio S. Combined use of trazodone-naltrexone versus clonidine-naltrexone in rapid withdrawal from methadone treatment. A comparative inpatient study. Drug and Alcohol Dependence. 2000;59(3):287-94. | Exclude: Paper assessing withdrawal; All patients aimed to be inducted onto naltrexone following detoxification; No prevalence estimate of number of patients opting to initiate naltrexone calculable. Nil discontinuation of naltrexone data reported. |
| 396. Prakash S, Balhara Y. Perceptions Related to Pharmacological Treatment of Opioid Dependence Among Individuals Seeking Treatment at a Tertiary Care Center in Northern India: A Descriptive Study. Substance Use & Misuse. 2016;51(7):861-9. | Exclude: Cross sectional survey of patients attitudes to naltrexone and buprenorphine |
| 397. Prieto IJ, Poyo-Guerrero R, Fernandez R, Ochoa E. Ultrarapid high-dose methadone detoxification [1]. Psychopharmacology. 2003;165(4):430. | Exclude: Letter relating to ultra-rapid detoxification |
| 398. Psaras C, Balkovski SL, Seamans M. Sociodemographic factors and medications for opioid use disorder (moud) initiation and retention: A review. Pharmacoepidemiology and Drug Safety. 2022;31(Supplement 2):327. | Exclude: Conference Abstract; No empirical data reported on the prevalence of or the factors associated with naltrexone initiation/discontinuation |
| 399. Rabinowitz J, Cohen H, Kotler M. Outcomes of ultrarapid opiate detoxification combined with naltrexone maintenance and counseling. Psychiatric Services. 1998;49(6):831-3. | Exclude: No empirical data reported on the prevalence of or the factors associated with naltrexone initiation/discontinuation |
| 400. Raistrick D, West D, Finnegan O, Thistlethwaite G, Brearley R, Banbery J. A comparison of buprenorphine and lofexidine for community opiate detoxification: Results from a randomized controlled trial. Addiction. 2005;100(12):1860-7. | Exclude: Reports number of people abstinent from opioids on naltrexone not rate of discontinuation |
| 401. Raith K, Hochhaus G. Drugs used in the treatment of opioid tolerance and physical dependence: A review. International Journal of Clinical Pharmacology and Therapeutics. 2004;42(4):191-203. | Exclude: Review article all references checked |
| 402. Ramsey SE, Rounsaville D, Hoskinson R, Park TW, Ames EG, Neirinckx VD, et al. The need for psychosocial interventions to facilitate the transition to extended-release naltrexone (XR-NTX) treatment for opioid dependence: A concise review of the literature. Substance Abuse: Research and Treatment. 2016;10:65-8. | Exclude: Review article all references checked for inclusion |
| 403. Randall-Kosich O, Andraka-Christou B. Reasons for Starting and Stopping Methadone, Buprenorphine, and Naltrexone Treatment. Journal of Addiction Medicine. 2020;14(6):e414-e5. | Exclude: Qualitative study of reasons for MOUD discontinuation |
| 404. Randall-Kosich O, Andraka-Christou B, Totaram R, Alamo J, Nadig M. Comparing Reasons for Starting and Stopping Methadone, Buprenorphine, and Naltrexone Treatment Among a Sample of White Individuals With Opioid Use Disorder. Journal of Addiction Medicine. 2020;14(4):e44-e52. | Exclude: Cross sectional qualitative interviews ascertaining reasons for dropout of treatment. |
| 405. Rashid S, Saleem F, Hanif S, Imam H, Omer SM, Farooq M, et al. Efficacy of naltrexone in preventing relapse in opioid dependent patients. Pakistan Journal of Medical and Health Sciences. 2012;6(3):549-51. | Exclude: Clinical trial which reports relapse to opioid use. Does not report calculable rates of naltrexone discontinuation |
| 406. Rawson RA, Glazer M, Callahan EJ, Liberman RP. Naltrexone and behavior therapy for heroin addiction. NIDA Research Monograph Series. 1979;25:26-43. | Exclude: Clinical trial reports mean number of dosage days of naltrexone and number of weeks in treatment defined as “number of weeks clients remained in contact”. Does not allow calculation of proportion of people discontinuing naltrexone - reports number of people who “stayed in treatment” not number with naltrexone discontinuation. |
| 407. Rawson RA, McCann MJ, Shoptaw SJ, Miotto KA, Forsch DL, Obert JL, et al. Naltrexone for opioid dependence: Evaluation of a manualized psychosocial protocol to enhance treatment response. Drug and Alcohol Review. 2001;20(1):67-78. | Exclude: Reports retention by weeks in treatment and number of doses of naltrexone taken. No data enabling a calculable rate of discontinuation reported. |
| 408. Rawson RA, Tennant Jr FS. Five-year follow-up of opiate addicts with naltrexone and behavior therapy. NIDA Research Monograph Series. 1984;NO. 49:289-95. | Exclude: Reports five-year outcomes of screened study Rawson 1979 which reports six month outcomes but reports retention not naltrexone discontinuation. |
| 409. Rawson RA, Washton AM, Resnick RB, Tennant FS, Jr. Clonidine hydrochloride detoxification from methadone treatment--the value of naltrexone aftercare. Adv Alcohol Subst Abuse. 1984;3(3):41-9. | Exclude: Paper assessing induction onto naltrexone; All patients aimed to be inducted onto naltrexone following detoxification; Nil other treatment options available and no prevalence estimate of number of patients opting to initiate naltrexone calculable. Nil discontinuation data reported. |
| 410. Reece AS. Psychosocial and treatment correlates of opiate free success in a clinical review of a naltrexone implant program. Subst Abuse Treat Prev Policy. 2007;2:35. | Exclude: Reports cohort of patients receiving oral or different formulations of naltrexone implants with differing length of naltrexone coverage. Although it reports the number of patients receiving 1, 2, 3, 3+ etc implants unclear which formulation these are thus unable to calculate rate of discontinuation at any prespecified specific time point. Opioid free survival reported no naltrexone discontinuation data. |
| 411. Reece AS. Comparative treatment and mortality correlates and adverse event profile of implant naltrexone and sublingual buprenorphine. Journal of Substance Abuse Treatment. 2009;37(3):256-65. | Exclude: Reports cohort of patients receiving different formulations of naltrexone implants with differing length of naltrexone coverage. Although it reports the number of patients receiving 1, 2, 3, 3+ etc implants unclear which formulation these are thus unable to calculate rate of discontinuation at any prespecified specific time point. |
| 412. Reece AS. Favorable mortality profile of naltrexone implants for opiate addiction. Journal of Addictive Diseases. 2010;29(1):30-50. | Exclude: Does not report data to calculate the number of people discontinuing the naltrexone implant either not replacing therapy when ended via dosage regimen or removal during active dosing. |
| 413. Reece AS. Epidemiologic and molecular pathophysiology of chronic opioid dependence and the place of naltrexone extended- release formulations in its clinical management. Substance Abuse: Research and Treatment. 2012;6(1):115-33. | Exclude: Review article all references checked for inclusion |
| 414. Renault PF. Treatment of heroin-dependent persons with antagonists: current status. NIDA Res Monogr. 1981;28:11-22. | Exclude: Review article all references checked |
| 415. Resnick RB, Kestenbaum RS, Washton A, Poole D. Naloxone-precipitated withdrawal: a method for rapid induction onto naltrexone. Clin Pharmacol Ther. 1977;21(4):409-13. | Exclude: Paper assessing induction onto naltrexone; All patients aimed to be inducted onto naltrexone following detoxification; Nil other treatment options available and no prevalence estimate of number of patients opting to initiate naltrexone calculable. Nil discontinuation data reported. |
| 416. Rezaiyan MK, Moghadam HK, Khosrojerdi H, Afshari R. Very low-dose naltrexone versus placebo in alleviating withdrawal manifestation. Clinical Toxicology. 2014;1):368. | Exclude: Conference abstract of withdrawal comparing naltrexone to placebo. |
| 417. Ring BM, Jarvis BP, Sigurdsson SO, DeFulio A, Silverman K. Propensity to work among detoxified opioid-dependent adults. Journal of Vocational Rehabilitation. 2018;49(2):187-94. | Exclude: Secondary analyses of three screened studies reporting on propensity to work |
| 418. Roberts K. Treatment and detoxification of opiate dependent drug users in different countries. Journal of the Royal Society of Health. 1989;109(3):100-5+9. | Exclude: Review article of detoxification practice |
| 419. Robertson AG, Easter MM, Lin HJ, Frisman LK, Swanson JW, Swartz MS. Associations between pharmacotherapy for opioid dependence and clinical and criminal justice outcomes among adults with co-occurring serious mental illness. Journal of Substance Abuse Treatment. 2018;86:17-25. | Exclude: Explore association between MOUD and criminal justice and other outcomes. Nil data relating to rate of initiation or discontinuation reported |
| 420. Robertson AG, Swartz MS. Extended-release naltrexone and drug treatment courts: Policy and evidence for implementing an evidence-based treatment. Journal of Substance Abuse Treatment. 2018;85:101-4. | Exclude: Review article all references checked for inclusion |
| 421. Roe M, Wilson CG, Fusco CW, Hulkower S, Stigleman S. Does XR injectable naltrexone prevent relapse as effectively as daily sublingual buprenorphine-naloxone? Journal of Family Practice. 2020;69(10):E14-E5. | Exclude: Review article all references checked for inclusion |
| 422. Roman PM, Ducharme LJ, Knudsen HK. Patterns of organization and management in private and public substance abuse treatment programs. Journal of Substance Abuse Treatment. 2006;31(3):235-43. | Exclude: Examination of treatment modality availability |
| 423. Roozen HG, Boulogne JJ, Van Tulder MW, Van Den Brink W, De Jong CAJ, Kerkhof AJFM. A systematic review of the effectiveness of the community reinforcement approach in alcohol, cocaine and opioid addiction. Drug and Alcohol Dependence. 2004;74(1):1-13. | Exclude: Review article examining community reinforcement: All references checked for additional citations |
| 424. Roozen HG, de Waart R, van der Windt DA, van den Brink W, de Jong CA, Kerkhof AJ. A systematic review of the effectiveness of naltrexone in the maintenance treatment of opioid and alcohol dependence. European Neuropsychopharmacology. 2006;16(5):311-23. | Exclude: Systematic Review of naltrexone maintenance; All references checked for inclusion |
| 425. Roozen HG, Kerkhof AJ, van den Brink W. Experiences with an outpatient relapse program (community reinforcement approach) combined with naltrexone in the treatment of opioid-dependence: effect on addictive behaviors and the predictive value of psychiatric comorbidity. European Addiction Research. 2003;9(2):53-8. | Exclude: Paper assessing follow up of naltrexone; All patients aimed to be inducted onto naltrexone following detoxification; No prevalence estimate of number of patients opting to initiate naltrexone calculable. Nil discontinuation of naltrexone data reported only relapse |
| 426. Rosen M, Kosten TR. Detoxification and induction onto naltrexone.  Buprenorphine: Combatting drug abuse with a unique opioid. New York, NY: Wiley-Liss; US; 1995. p. 289-305. | Exclude: Paper assessing opioid withdrawal; All patients aimed to be inducted onto naltrexone following detoxification; Nil other treatment options available and no prevalence estimate of number of patients opting to initiate naltrexone calculable. Nil discontinuation data reported. |
| 427. Rosenberg H, Phillips KT. Acceptability and availability of harm-reduction interventions for drug abuse in American substance abuse treatment agencies. Psychology of Addictive Behaviors. 2003;17(3):203-10. | Exclude: Survey of treatment agencies |
| 428. Rothenberg JL, Sullivan MA, Church SH, Seracini A, Collins E, Kleber HD, et al. Behavioral naltrexone therapy: An integrated treatment for opiate dependence. Journal of Substance Abuse Treatment. 2002;23(4):351-60. | Exclude: Reports retention “The principal outcome measure was retention in treatment. Participants were removed from the trial and counted as dropouts if they ceased naltrexone and resumed opiate use with physiological dependence” |
| 429. Rotrosen J. Comparative effectiveness of extended-release naltrexone vs. Buprenorphine for opioid dependence treatment-NIDA CTN-0051. Neuropsychopharmacology. 2017;43(Supplement 1):S60-S1. | Exclude: Conference abstract of design and methods of screened clinical trial |
| 430. Rounsaville BJ. Can psychotherapy rescue naltrexone treatment of opioid addiction? NIDA Research Monograph Series. 1995(150):37-52. | Exclude: Review article all references checked |
| 431. Rozenberg I. The use of naltrexone among young adults with opioid use disorder. Dissertation Abstracts International: Section B: The Sciences and Engineering. 2020;81(12-B):No Pagination Specified. | Exclude: Thesis all references checked for inclusion; reports on retention in treatment not discontinuation |
| 432. Rudolf G, Walsh J, Baston K, Gianutsos P, Rudolf V. A non-opioid protocol for outpatient opioid detoxification and transition to antagonist treatment. Journal of Addiction Medicine. 2016;10(3):E5-E6. | Exclude: Conference abstract duplicate data from subsequent included study Rudolf 2018 with more participants |
| 433. Ruger JP, Chawarski M, Mazlan M, Ng N, Schottenfeld R. Cost-effectiveness of buprenorphine and naltrexone treatments for heroin dependence in Malaysia. PLoS ONE. 2012;7(12):e50673. | Exclude: Cost effectiveness analysis of clinical trial. Reports retention in treatment. Does not report calculable rates of naltrexone discontinuation |
| 434. Ruglass LM, Scodes J, Pavlicova M, Campbell ANC, Fitzpatrick S, Barbosa-Leiker C, et al. Trajectory classes of opioid use among individuals in a randomized controlled trial comparing extended-release naltrexone and buprenorphine-naloxone. Drug and Alcohol Dependence. 2019;205(no pagination). | Exclude: Secondary analysis of screened clinical trial examining classes of patient relapsing to opioid use |
| 435. Saeed SA, Stanciu C. Injectable extended-release naltrexone for opioid dependence: 3 studies. Current Psychiatry. 2019;18(1):39-42. | Exclude: Review editorial all references checked for inclusion |
| 436. Safari F, Mottaghi K, Malek S, Salimi A. Effect of ultra-rapid opiate detoxification on withdrawal syndrome. Journal of Addictive Diseases. 2010;29(4):449-54. | Exclude: Study of withdrawal; Nil calculable rates of naltrexone discontinuation reported |
| 437. Samet JH, Blokhina E, Cheng DM, Rosen S, Lioznov D, Lunze K, et al. Rapid access to antiretroviral therapy, receipt of naltrexone, and strengths-based case management versus standard of care for HIV viral load suppression in people with HIV who inject drugs in Russia (LINC-II): an open-label, randomised controlled trial. The Lancet HIV. 2023;10(9):e578-e87. | Exclude: Clinical trial; Nil calculable data reported for initiation of naltrexone in an eligible population. Discontinuation data not calculable: Each intervention participant scheduled to receive depot NTX-XR and then offered up to 4 12-week naltrexone implants. Unclear number of people initiating naltrexone implant or numbers continuing “Of the 111 participants in the intervention group, 63 (57%) received a naltrexone injection at baseline and ten (9%) received four implants at follow-up” |
| 438. San L, Pomarol G, Peri JM, Olle JM, Cami J. Follow-up after a six-month maintenance period on naltrexone versus placebo in heroin addicts. British Journal of Addiction. 1991;86(8):983-90. | Exclude: Clinical trial reporting retention in treatment “Retention rate was defined as the percentage of patients who completed the study and did not miss five consecutive visits or |
| 439. Saunders EC, Budney AJ, Cavazos-Rehg P, Scherer E, Bell K, John D, et al. Evaluating preferences for medication formulation and treatment model among people who use opioids non-medically: A web-based cross-sectional study. Journal of Substance Use and Addiction Treatment. 2024;163(no pagination). | Exclude: 10 alternative visits (unless unforeseen circumstances had occurred) or positive opioid-screening urine samples (i.e. presence of heroin) had been presented more than five times” No discontinuation data reported. |
| 440. Saunders EC, Moore SK, Walsh O, Metcalf SA, Budney AJ, Scherer E, et al. Perceptions and preferences for long-acting injectable and implantable medications in comparison to short-acting medications for opioid use disorders. Journal of Substance Abuse Treatment. 2020;111:54-66. | Exclude: Cross sectional survey of preferences for medication for opioid use disorder |
| 441. Schacht JP, Randall PK, Latham PK, Voronin KE, Book SW, Myrick H, et al. Predictors of Naltrexone Response in a Randomized Trial: Reward-Related Brain Activation, OPRM1 Genotype, and Smoking Status. Neuropsychopharmacology. 2017;42(13):2640-53. | Exclude: Qualitative Study; No empirical data on number of people initiating or discontinuing naltrexone |
| 442. Schottenfeld RS, Chawarski MC, Mazlan M. Maintenance treatment with buprenorphine and naltrexone for heroin dependence in Malaysia: a randomised, double-blind, placebo-controlled trial. Lancet. 2008;371(9631):2192-200. | Exclude: Alcohol use disorder population |
| 443. Scorsone KL. Exploring individual experiences obtaining medication-assisted treatment for opioid use disorder in rural New Mexico. Dissertation Abstracts International: Section B: The Sciences and Engineering. 2020;81(3-B):No Pagination Specified. | Exclude: Reports retention not discontinuation of naltrexone |
| 444. Seivewright N. Community treatment of drug misuse: More than methadone. New York, NY: Cambridge University Press; US; 2000. | Exclude: Qualitative dissertation study of experiences of accessing medication for opioid use disorder |
| 445. Seivewright N, Iqbal MZ. Prescribing to drug misusers in practice--often effective, but rarely straightforward. Addiction Biology. 2002;7(3):269-77. | Exclude: Book and review of opioid use disorder management: all references checked |
| 446. Senft RA. Experience with clonidine-naltrexone for rapid opiate detoxification. Journal of Substance Abuse Treatment. 1991;8(4):257-9. | Exclude: Review article all references checked |
| 447. Seoane A, Carrasco G, Cabre L, Puiggros A, Hernandez E, Alvarez M, et al. Efficacy and safety of two new methods of rapid intravenous detoxification in heroin addicts previously treated without success. British Journal of Psychiatry. 1997;171:340-5. | Exclude: Paper assessing induction onto naltrexone; All patients aimed to be inducted onto naltrexone following detoxification; Nil other treatment options available and no prevalence estimate of number of patients opting to initiate naltrexone calculable. Nil discontinuation data reported. |
| 448. Shafa R, Abdolmaleky HM, Eskandari M, Yaqubi S, Ghaemi SN. Utilizing (catechol-o-methyltransferase) comtinhibition effect in detoxification off buprenorphine, methadone and other opioids. American Journal on Addictions. 2013;22(3):309-10. | Exclude: Paper assessing opioid withdrawal; All patients aimed to be inducted onto naltrexone following detoxication; Nil other treatment options available and no prevalence estimate of number of patients opting to initiate naltrexone calculable. Nil discontinuation data reported. |
| 449. Sharma Haase K, Kunoe N, Opheim A, Gaulen Z, Nja AM, Latif ZE, et al. Interest in Extended Release Naltrexone among Opioid Users. European Addiction Research. 2016;22(6):301-5. | Exclude: Conference abstract using entacapone in withdrawal |
| 450. Shi JM, O'Connor PG, Constantino JA, Carroll KM, Schottenfeld RS, Rounsaville BJ. Three methods of ambulatory opiate detoxification: Preliminary results of a randomized clinical trial. NIDA Research Monograph Series. 1993(132):309. | Exclude: Cross sectional survey of people who use opioids about interest in naltrexone |
| 451. Shi Z, Jagannathan K, Wiers CE, O'Brien CP, Childress AR, Langleben DD. Resting-state nucleus accumbens connectivity predicts adherence to extended-release naltrexone treatment in opioid use disorder. Neuropsychopharmacology. 2020;Conference: 59th Annual Meeting of the American College of Neuropsychopharmacology, ACNP 2020. Virtual. 45:356-7. | Exclude: Conference abstract of O’Connor 1997 |
| 452. Shouan A, Ghosh A, Singh S, Basu D, Mattoo S. Predictors of retention in the treatment for opioid dependence: A prospective, observational study from India. Indian Journal of Psychiatry. 2021;63(4):355-65. | Exclude: Conference Abstract of data from University of Pennsylvania at some time with same authors as included sata on larger full study cohort reported in Wang et al. Exclude due to potential duplicate data |
| 453. Shufman EN, Porat S, Witztum E, Gandacu D, Bar-Hamburger R, Ginath Y. The efficacy of naltrexone in preventing reabuse of heroin after detoxification. Biological psychiatry. 1994;35(12):935-45. | Exclude: Reports treatment retention and follow up not discontinuation rates of naltrexone. |
| 454. Shulman M, Choo TH, Scodes J, Pavlicova M, Wai J, Haenlein P, et al. Association between methadone or buprenorphine use during medically supervised opioid withdrawal and extended-release injectable naltrexone induction failure. Journal of Substance Abuse Treatment. 2021;124:108292. | Exclude: Clinical trial reporting retention in treatment trial as outcome not naltrexone discontinuation. |
| 455. Shulman M, Hu MC, Sullivan MA, Akerman SC, Fratantonio J, Barbieri V, et al. Patient characteristics associated with initiation of XR-naltrexone for opioid use disorder in clinical trials. Drug Alcohol Depend. 2022;233:109343. | Exclude: Secondary analysis of screened clinical trial assessing withdrawal outcomes |
| 456. Shulman M, Provost S, Ohrtman K, Novo P, Meyers-Ohki S, Van Veldhuisen P, et al. Discontinuation of medication treatment for opioid use disorder after a successful course: The discontinuation phase of the CTN-0100 (RDD) trial. Contemporary Clinical Trials. 2024;142:107543. | Exclude: Systematic review all references checked for inclusion |
| 457. Shulman M, Weiss R, Rotrosen J, Novo P, Costello E, Nunes EV. Prior National Drug Abuse Treatment Clinical Trials Network (CTN) opioid use disorder trials as background and rationale for NIDA CTN-0100 "optimizing retention, duration and discontinuation strategies for opioid use disorder pharmacotherapy (RDD)". Addict Sci Clin Pract. 2021;16(1):15. | Exclude: Reports discontinuation rates after 6 months for those stable on NTX-XR |
| 458. Sibai M, Mishlen K, Nunes EV, Levin FR, Mariani JJ, Bisaga A. A week-long outpatient induction onto XR-naltrexone in patients with opioid use disorder. Am J Drug Alcohol Abuse. 2020;46(3):289-96. | Exclude: Discussion paper of future clinical trial not including naltrexone |
| 459. Sigmon SC, Bisaga A, Nunes EV, O'Connor PG, Kosten T, Woody G. Opioid detoxification and naltrexone induction strategies: recommendations for clinical practice. Am J Drug Alcohol Abuse. 2012;38(3):187-99. | Exclude: Reports on induction technique for XR-NTX naltrexone Does not report on a cohort or give number of people detoxified eligible and initiating naltrexone therapy only number entering detoxification; Nil discontinuation data reported |
| 460. Sigmon SC, Dunn KE, Badger GJ, Heil SH, Higgins ST. Brief buprenorphine detoxification for the treatment of prescription opioid dependence: a pilot study. Addictive Behaviors. 2009;34(3):304-11. | Exclude: Review article all references checked for inclusion |
| 461. Sigmon SC, Dunn KE, Saulsgiver K, Patrick ME, Badger GJ, Heil SH, et al. A randomized, double-blind evaluation of buprenorphine taper duration in primary prescription opioid abusers. JAMA Psychiatry. 2013;70(12):1347-54. | Exclude: No empirical data reported to for calculable naltrexone initiation or discontinuation. |
| 462. Simon DL. The rationale for naltrexone therapy as an alternative to methadone treatment for opiate addiction. Connecticut Medicine. 1996;60(11):683-5. | Exclude: No empirical data reported to allow calculation of proportion of people discontinuing naltrexone; retention in treatment reported |
| 463. Singh VV, Sarkar S, Chadda RK, Mishra AK, Dhawan A. Reasons for leaving treatment among patients with opioid dependence: A 3-month prospective follow-up study. Journal of Opioid Management. 2022;18(5):455-66. | Exclude: No empirical data reported on the prevalence of or the factors associated with naltrexone initiation/discontinuation |
| 464. Singla V. Correlates of opioid abstinence in patients maintained on naltrexone as compared to buprenorphine. Indian Journal of Psychiatry. 2020;62(7 Supplement 1):S34-S5. | Exclude: Prospective observational study nil data on rates of initiation or discontinuation reported |
| 465. Singleton EG, Sherman MF, Bigelow GE. The index of choice: indications of methadone patients' selection of naltrexone treatment. Am J Drug Alcohol Abuse. 1984;10(2):209-21. | Exclude: Conference Abstract; No empirical data reported on the prevalence of or the factors associated with naltrexone initiation/discontinuation. No report of discontinuations. |
| 466. Sinha R, Kimmerling A, Doebrick C, Kosten TR. Effects of lofexidine on stress-induced and cue-induced opioid craving and opioid abstinence rates: preliminary findings. Psychopharmacology. 2007;190(4):569-74. | Exclude: Cohort of patients on methadone and examines interest in naltrexone therapy. Does not report number of patients post detoxification and eligible for naltrexone who initiate naltrexone therapy. |
| 467. Soares WE, 3rd, Wilson D, Gordon MS, Lee JD, Nunes EV, O'Brien CP, et al. Incidence of future arrests in adults involved in the criminal justice system with opioid use disorder receiving extended release naltrexone compared to treatment as usual. Drug Alcohol Depend. 2019;194:482-6. | Exclude: Trial of lofexidine as an adjunct; treatment completion reported nil calculable rate of naltrexone discontinuation reported |
| 468. Soares WE, 3rd, Wilson D, Rathlev N, Lee JD, Gordon M, Nunes EV, et al. Healthcare utilization in adults with opioid dependence receiving extended release naltrexone compared to treatment as usual. Journal of Substance Abuse Treatment. 2018;85:66-9. | Exclude: Secondary analysis of screened trial reporting criminal justice outcomes |
| 469. Sofuoglu M, Devito EE, Carroll KM. Pharmacological and behavioral treatment of opioid use disorder. Psychiatric Research and Clinical Practice. 2019;1(1):4-15. | Exclude: Secondary analysis of screened clinical trial examining healthcare utilisation data |
| 470. Sofuoglu M, Kosten TR. Pharmacologic management of relapse prevention in addictive disorders. Psychiatric Clinics of North America. 2004;27(4):627-48. | Exclude: Review article all references checked for inclusion |
| 471. Solli K, Opheim A, Latif ZEH, Kunoe N, Tanum L. The effectiveness of injectable extended release naltrexone versus daily buprenorphine-naloxone for opioid dependence: A randomized clinical trial. Neurology Conference: 70th Annual Meeting of the American Academy of Neurology, AAN. 2018;90(15 Supplement 1). | Exclude: Review article all references checked for inclusion |
| 472. Springer SA, Brown SE, Di Paola A, Altice FL. Correlates of retention on extended-release naltrexone among persons living with HIV infection transitioning to the community from the criminal justice system. Drug Alcohol Depend. 2015;157:158-65. | Exclude: Conference abstract of Tanum 2017/Soli 2018. Exclude to prevent duplicate data |
| 473. Stafford C, Marrero WJ, Naumann RB, Lich KH, Wakeman S, Jalali MS. Identifying key risk factors for premature discontinuation of opioid use disorder treatment in the United States: A predictive modeling study. Drug and Alcohol Dependence. 2022;237(no pagination). | Exclude: Preliminary data from full included trial Springer 2018. Excluded to prevent double counting. |
| 474. Stein MD, Anderson BJ, Bailey GL. Preferences for Aftercare Among Persons Seeking Short-Term Opioid Detoxification. Journal of Substance Abuse Treatment. 2015;59:99-103. | Exclude: Modelling study to predict treatment dropout. Nil naltrexone initiation or discontinuation data reported |
| 475. Stella L, Cassese F, Barone S, Barchetta A, Iacobelli M, Motola G, et al. Naltrexone to keep a drug-free condition. Research Communications in Alcohol and Substances of Abuse. 1999;20(3-4):91-8. | Exclude: Cross sectional survey of post detoxification treatment modality preferences |
| 476. Stella L, D'Ambra C, Mazzeo F, Capuano A, Del Franco F, Avolio A, et al. Naltrexone plus benzodiazepine aids abstinence in opioid-dependent patients. Life Sciences. 2005;77(21):2717-22. | Exclude: Clinical trial with drop out from trial reported as primary outcome not naltrexone discontinuation. |
| 477. Stotts AL, Dodrill CL, Kosten TR. Opioid dependence treatment: options in pharmacotherapy. Expert Opinion on Pharmacotherapy. 2009;10(11):1727-40. | Exclude: Clinical trial: Reports opioid relapse rates not discontinuation rates |
| 478. Strang J, Kelleher M, Mayet S, Day E, Hellier J, Byford S, et al. Extended-release naltrexone versus standard oral naltrexone versus placebo for opioid use disorder: the NEAT three-arm RCT. Health Technol Assess. 2019;23(3):1-72. | Exclude: Review article all references checked |
| 479. Streel E, Chenut C, Papageorgiou C, Verbanck P. DSM IV axis II traits can influence compliance to treatment with oral naltrexone: a preliminary study on 30 opiate dependent patients. Addictive Behaviors. 2014;39(1):321-4. | Exclude: Six patients recruited nil discontinuation data reported |
| 480. Streel E, Verbanck P. Ultra-rapid opiate detoxification: from clinical applications to basic science. Addiction Biology. 2003;8(2):141-6. | Exclude: Does not report number of people with 0,3 and 6 months of naltrexone compliance only their SCID scores |
| 481. Suffet F, Remine DC, 2nd, Taleporos E, Brotman R. Naltrexone and conventionality. Am J Drug Alcohol Abuse. 1978;5(2):221-33. | Exclude: Review article of rapid detoxification; All references checked |
| 482. Sullivan M, Mannelli P, Yu M, Nangia N, Graham C, Webster I, et al. Outpatient transition to extended-release naltrexone in patients with opioid-use disorder. American Journal on Addictions. 2018;27(4):323. | Exclude: Double counting already included as part of Resnick 1978 |
| 483. Sullivan MA, Bisaga A, Carpenter K, Mariani JJ, Mishlen K, Nunes EV. Long-acting injectable naltrexone induction: A randomized trial of outpatient opioid detoxification with naltrexone vs. Buprenorphine. Drug and Alcohol Dependence. 2015;Conference: 2014 Annual Meeting of the College on Problems of Drug Dependence. San Juan Puerto Rico. 146:e112. | Exclude: Conference Abstract of Bisaga 2018; Screened clinical trial: Exclude due to duplicate data |
| 484. Sullivan MA, Bisaga A, Glass A, Mishlen K, Pavlicova M, Carpenter KM, et al. Opioid use and dropout in patients receiving oral naltrexone with or without single administration of injection naltrexone. Drug Alcohol Depend. 2015;147:122-9. | Exclude: Conference abstract of included study excluded to avoid double counting |
| 485. Sullivan MA, Bisaga A, Mariani JJ, Glass A, Levin FR, Comer SD, et al. Naltrexone treatment for opioid dependence: does its effectiveness depend on testing the blockade? Drug Alcohol Depend. 2013;133(1):80-5. | Exclude: Clinical trial of 4 arms all of which include oral naltrexone with two arms additionally using XR-NTX or placebo injections. Discontinuation rate data for stopping all forms of naltrexone was not calculable. Paper reports retention in treatment arms described as the number of participants who completes x number of weeks of treatment based on attendance not on naltrexone discontinuation “ of patients who did not complete the trial, 48 were non-compliant with attendance (i.e., dropped out/relapsed) and 18 relapsed to opiate use while still attending sessions” |
| 486. Sullivan MA, Bisaga A, Pavlicova M, Carpenter KM, Choi CJ, Mishlen K, et al. A Randomized Trial Comparing Extended-Release Injectable Suspension and Oral Naltrexone, Both Combined With Behavioral Therapy, for the Treatment of Opioid Use Disorder. American Journal of Psychiatry. 2019;176(2):129-37. | Exclude: Post-hoc analysis of screened data from Comer 2006. Reports retention in treatment defined as clinic attendance not discontinuation data. |
| 487. Sullivan MA, Garawi F, Bisaga A, Comer SD, Carpenter K, Raby WN, et al. Management of relapse in naltrexone maintenance for heroin dependence. Drug Alcohol Depend. 2007;91(2-3):289-92. | Exclude: Reports retention in treatment and “Time to dropout (last on-site clinical contact) not discontinuation of naltrexone |
| 488. Sullivan MA, Rothenberg JL, Vosburg SK, Church SH, Feldman SJ, Epstein EM, et al. Predictors of retention in naltrexone maintenance for opioid dependence: analysis of a stage I trial. American Journal on Addictions. 2006;15(2):150-9. | Exclude: Reports results from two screened clinical trials which report retention in treatment as their outcome not discontinuation from naltrexone data reported. Reports mean compliance using riboflavin urine assay but not numbers or rate of naltrexone discontinuation. |
| 489. Sullivan MA, Vosburg SK, Comer SD. Depot naltrexone: antagonism of the reinforcing, subjective, and physiological effects of heroin. Psychopharmacology. 2006;189(1):37-46. | Exclude: Duplicate data of screened trial Rothenburg 2002, original trial reports retention “The principal outcome measure was retention in treatment. Participants were removed from the trial and counted as dropouts if they ceased naltrexone and resumed opiate use with physiological dependence” |
| 490. Syed YY, Keating GM. Extended-release intramuscular naltrexone (VIVITROL R): a review of its use in the prevention of relapse to opioid dependence in detoxified patients. CNS Drugs. 2013;27(10):851-61. | Exclude: No empirical data reported on the prevalence of or the factors associated with naltrexone initiation/discontinuation; n=5 |
| 491. Tait RJ, Ngo HTT, Hulse GK. Mortality in heroin users 3 years after naltrexone implant or methadone maintenance treatment. Journal of substance abuse treatment. 2008;35(2):116-24. | Exclude: Review article all references checked for inclusion |
| 492. Tanum L, Solli K, Latif ZEH, Benth JS, Opheim A, Krajci P, et al. The effectiveness of injectable extended release naltrexone versus daily buprenorphine-naloxone for opioid dependence in short and long term treatment. Biological Psychiatry. 2018;83(9 Supplement 1):S454. | Exclude: Does not report discontinuation data of individuals receiving number of implants, only first implant received. Describes three cases implant removed in the first week but not in the analyses cohort and does not report any further implant removals. No calculable discontinuation rates |
| 493. Tanum LH, Opheim A, Solli K, Sharma-Haase K, Latif ZEH, Kunoe N. Optimal prevention of relapse among opioid users: A 12-week randomized controlled trial of extended-release naltrexone injections versus daily buprenorphine-naloxone. Drug and Alcohol Dependence. 2017;Conference: 2016 Annual Meeting of the College on Problems of Drug Dependence, CPDD 2016. Palm Springs, CA United States. 171:e200-e1. | Exclude: Conference Abstract; No empirical data reported on the prevalence of or the factors associated with naltrexone initiation/discontinuation |
| 494. Teplin D, Raz B, Daiter J, Varenbut M, Zachos CT, Whang P, et al. Measurement of symptom withdrawal severity in a 24-hour period after the anesthesia-assisted rapid opiate detoxification procedure. Am J Drug Alcohol Abuse. 2005;31(2):327-35. | Exclude: Conference abstract of clinical trial; Nil discontinuation or initiation data presented |
| 495. Tetrault JM, Fiellin DA. Current and potential pharmacological treatment options for maintenance therapy in opioid-dependent individuals. Drugs. 2012;72(2):217-28. | Exclude: Does not report prevalence of initiation or discontinuation |
| 496. Thackway SV, Ward PL, Taylor LK. Active surveillance of adverse events associated with naltrexone in New South Wales. Medical Journal of Australia. 1999;171(9):499-500. | Exclude: Review article all references checked for inclusion |
| 497. Thomas M, Kauders F, Harris M, Cooperstein J, Hough G, Resnick R. Clinical experiences with naltrexone in 370 detoxified addicts. NIDA Res Monogr. 1976(9):88-92. | Exclude: Previous publication of data in Resnick 1978 excluded to prevent double counting more recent numbers published |
| 498. Throckmorton DC, Temple R, Rappaport BA, Roca R, Winchell CJ. Injectable extended-release naltrexone for opioid dependence. The Lancet. 2011;378(9792):665-6. | Exclude: Commentary article |
| 499. Tiihonen J, Krupitsky E, Verbitskaya E, Blokhina E, Mamontova O, Föhr J, et al. Naltrexone implant for the treatment of polydrug dependence: a randomized controlled trial. American Journal of Psychiatry. 2012;169(5):531-6. | Exclude: Reports retention in treatment in opioid and amphetamine use disorder co-morbid sample. |
| 500. Timko C, Schultz NR, Cucciare MA, Vittorio L, Garrison-Diehn C. Retention in medication-assisted treatment for opiate dependence: a systematic review. Journal of addictive diseases. 2016;35(1):22-35. | Exclude: Systematic review all references checked for inclusion |
| 501. Tofighi B, Williams AR, Chemi C, Suhail-Sindhu S, Dickson V, Lee JD. Patient Barriers and Facilitators to Medications for Opioid Use Disorder in Primary Care. Substance Use & Misuse. 2019;54(14):2409-19. | Exclude: Qualitative study; No empirical data on number of people initiating or discontinuing naltrexone |
| 502. Toh ZA, Wijesinghe PR. Clinical profiles of patients and their compliance with oral naltrexone for alcohol, opioid and gambling addiction in the national addictions management service at the institute of mental health (IMH), Singapore. Annals of the Academy of Medicine Singapore. 2014;1):S93. | Exclude: Conference abstract reporting on alcohol, opioid and gambling cohorts no separate data for opioid use disorder |
| 503. Tompkin DA, Sun X, Bisaga A, Sullivan M, Akerman S, Legedza A, et al. Daily withdrawal and craving scores as predictors of dropout during outpatient induction onto XR-NTX. American Journal on Addictions. 2019;28(3):216-7. | Exclude: Conference Abstract; Duplicate of screened included clinical trial Bisaga 2018. Duplicate data |
| 504. Tompkins CNE, Neale J, Marsden J, Strang J. Factors influencing recruitment to a randomised placebo-controlled trial of oral naltrexone and extended release implant naltrexone: Qualitative study. Journal of Substance Abuse Treatment. 2019;99:52-60. | Exclude: Qualitative study |
| 505. Tornay CB, Favrat B, Monnat M, Daeppen JB, Schnyder C, Bertschy G, et al. Ultra-rapid opiate detoxification using deep sedation and prior oral buprenorphine preparation: Long-term results. Drug and Alcohol Dependence. 2003;69(3):283-8. | Exclude: Paper assessing withdrawal; All patients aimed to be inducted onto naltrexone following detoxification; No prevalence estimate of number of patients opting to initiate naltrexone calculable. Nil discontinuation of naltrexone data calculable from report |
| 506. Tourdot NE. Comparison of Vivitrol and Suboxone in terms of lowering relapse among opioid addicts. Dissertation Abstracts International: Section B: The Sciences and Engineering. 2024;85(11-B):No Pagination Specified. | Exclude: Dissertation abstract no initiation or discontinuation of naltrexone data reported |
| 507. Tretter F, Burkhardt D, Bussello-Spieth B, Reiss J, Walcher S, Buchele W. Clinical experience with antagonist-induced opiate withdrawal under anaesthesia. Addiction. 1998;93(2):269-75. | Exclude: Paper assessing withdrawal strategies; All patients aimed to be inducted onto naltrexone following detoxication; Nil other treatment options available and no prevalence estimate of number of patients opting to initiate naltrexone calculable. Nil discontinuation data reported. |
| 508. Tucker T, Ritter A, Maher C, Jackson H. A randomized control trial of group counseling in a naltrexone treatment program. Journal of Substance Abuse Treatment. 2004;27(4):277-88. | Exclude: Reports rates of retention in treatment with no definition if this relates to naltrexone discontinuation. No discontinuation data calculable |
| 509. Tucker TK, Ritter AJ. Naltrexone in the treatment of heroin dependence: A literature review. Drug and Alcohol Review. 2000;19(1):73-82. | Exclude: Review article all references checked for inclusion |
| 510. Uebelacker LA, Bailey G, Herman D, Anderson B, Stein M. Patients' Beliefs About Medications are Associated with Stated Preference for Methadone, Buprenorphine, Naltrexone, or no Medication-Assisted Therapy Following Inpatient Opioid Detoxification. Journal of Substance Abuse Treatment. 2016;66:48-53. | Exclude: Cross sectional survey of attitudes to medications for opioid use disorder |
| 511. Veilleux JC, Colvin PJ, Anderson J, York C, Heinz AJ. A review of opioid dependence treatment: pharmacological and psychosocial interventions to treat opioid addiction. Clinical Psychology Review. 2010;30(2):155-66. | Exclude: Review article all references checked for inclusion |
| 512. Velasquez M, Flannery M, Badolato R, Vittitow A, McDonald RD, Tofighi B, et al. Perceptions of extended-release naltrexone, methadone, and buprenorphine treatments following release from jail. Addict Sci Clin Pract. 2019;14(1):37. | Exclude: Qualitative Study; No empirical data on number of people initiating or discontinuing naltrexone |
| 513. Verebey K. The clinical pharmacology of naltrexone: pharmacology and pharmacodynamics. NIDA Res Monogr. 1981;28:147-58. | Exclude: Pharmacokinetic study; No empirical data reported on the prevalence of or the factors associated with naltrexone initiation/discontinuation |
| 514. Waal H, Christophersen AS, Frogopsahl G, Olsen LH, Mørland J. Naltrexone implants--a pilot project. Tidsskrift for den Norske Laegeforening: Tidsskrift for Praktisk Medicin, ny Raekke. 2003;123(12):1660-1. | Exclude: No empirical data on naltrexone initiation or discontinuation |
| 515. Waal H, Frogopsahl G, Olsen L, Christophersen AS, Mørland J. Naltrexone implants–duration, tolerability and clinical usefulness: a pilot study. European Addiction Research. 2006;12(3):138-44. | Exclude: Pooled discontinuation data included in Kunoe 2010; Excluded to avoid double counting. Study level data extracted. |
| 516. Waal H, Kornor H. Abstinence-oriented therapies for opiate addicts. Current Opinion in Psychiatry. 2004;17(3):169-74. | Exclude: Review article all references checked |
| 517. Wachman EM, Saia K, Miller M, Valle E, Shrestha H, Carter G, et al. Naltrexone Treatment for Pregnant Women With Opioid Use Disorder Compared With Matched Buprenorphine Control Subjects. Clinical Therapeutics. 2019;41(9):1681-9. | Exclude: Six patients taking naltrexone; Nil discontinuation data reported |
| 518. Wakeman SE, Larochelle MR, Ameli O, Chaisson CE, McPheeters JT, Crown WH, et al. Comparative Effectiveness of Different Treatment Pathways for Opioid Use Disorder. JAMA Network Open. 2020;3(2):e1920622. | Exclude: Reports number of people with OUD receiving naltrexone as their “initial treatment pathway” but unclear if all population eligible for naltrexone; i.e., currently opioid free - some individuals chose detoxification other partial hospitalisation, unclear of denominator eligible for naltrexone as a relapse prevention medication, No discontinuation data reported |
| 519. Wang AL, Elman I, Lowen S, Blady SJ, Lynch KG, O'Brian CP, et al. Pharmacotherapy of opioid dependence with the extended-release injectable naltrexone. European Neuropsychopharmacology. 2014;2):S696. | Exclude: Conference Abstract of data from included study Shi et al. 2018. Exclude to avoid double counting - Wang 2015 full paper also includes same cohort and excluded. |
| 520. Wang AL, Elman I, Lowen SB, Blady SJ, Lynch KG, Hyatt JM, et al. Neural correlates of adherence to extended-release naltrexone pharmacotherapy in heroin dependence. Translational Psychiatry. 2015;5(3) (no pagination). | Exclude: Reports on same subject recruited at same time point and same institution as Shi et al. 2018. Excluded to prevent double counting. |
| 521. Wang L, Volkow ND, Berger NA, Davis PB, Kaelber DC, Xu R. Cardiac and mortality outcome differences between methadone, buprenorphine and naltrexone prescriptions in patients with an opioid use disorder. Journal of Clinical Psychology. 2023;79(12):2869-83. | Exclude: Prescription data for cohort of people on medications for opioid use disorder examining cardiac outcomes; Nil initiation or discontinuation data reported |
| 522. Weimand BM, Solli KK, Reichelt WH, Tanum L. Enablers and hindrances for longer-term abstinence in opioid dependent individuals receiving treatment with extended-release naltrexone: A Norwegian longitudinal recovery trial (NaltRec study). Contemporary Clinical Trials Communications. 2021;21(no pagination). | Exclude: Protocol paper |
| 523. Weiss R, Nunes E, Novo P, Shulman M, Bailey G, Provost S, et al. Ethical and Methodological Challenges in Studying Discontinuation of Medications for Opioid Use Disorder. Drug and Alcohol Dependence. 2024;Conference: Abstracts from the 2023 Annual Meeting of the College on Problems of Drug Dependence. Downtown Denver Sheraton. | Exclude: Conference Abstract; No empirical data reported on the prevalence of or the factors associated with naltrexone initiation/discontinuation |
| 524. Weiss RD. Adherence to pharmacotherapy in patients with alcohol and opioid dependence. Addiction. 2004;99(11):1382-92. | Exclude: Review article all references checked for inclusion |
| 525. White JM, Lopatko OV. Opioid maintenance: A comparative review of pharmacological strategies. Expert Opinion on Pharmacotherapy. 2007;8(1):1-11. | Exclude: Review article all references checked for inclusion |
| 526. Wilcox RE, Erikson CK. Prevention of relapse to addiction: information for the practitioner. Tex Med. 2004;100(2):52-61. | Exclude: Review article all references checked |
| 527. Wildberger J, Wenzel KR, Burgower R, Fishman M. Relationship between injectable naltrexone and IOP utilization on opioid relapse in youth. Journal of Addiction Medicine. 2019;13(3):E36. | Exclude: Conference Abstract; No empirical data reported on the prevalence of or the factors associated with naltrexone initiation/discontinuation |
| 528. Williams AR, Barbieri V, Mishlen K, Levin FR, Nunes EV, Mariani JJ, et al. Long-term follow-up study of community-based patients receiving XR-NTX for opioid use disorders. American Journal on Addictions. 2017;26(4):319-25. | Exclude: Follow up study of included trial participants from Sullivan 2017; Reports number of injections received after study completion not for all participants; unable to calculate rate of discontinuation for timepoints other than included in original trial |
| 529. Wodak A. Drug treatment for opioid dependence. Australian Prescriber. 2001;24(1):4-6. | Exclude: Review article all references checked |
| 530. Woody G, Krupitsky E, Zvartau E, Blokhina E, Verbitskaya E, Lioznov D, et al. Naltrexone implant improves HIV treatment outcomes compared to oral naltrexone in opiate addicted patients. Neuropsychopharmacology. 2017;43(Supplement 1):S201-S2. | Exclude: Conference abstract of screened clinical trial reporting viral serology outcomes |
| 531. Woody G, Poole S, Krupitsky E, Kampman K. Transition to Antagonist Treatment in the Age of Fentanyl. Drug and Alcohol Dependence. 2024;Conference: Abstracts from the 2023 Annual Meeting of the College on Problems of Drug Dependence. Downtown Denver Sheraton. | Exclude: Does not report denominator clearly to enable calculation of number eligible to receive naltrexone who were offered it and number successfully initiating naltrexone |
| 532. Yaroslavtseva T, Krupitsky E, Zvartau E, Blokhina E, Woody G. The impact of oral and implantable formulations of naltrexone on overall functioning and social adjustment in opioid dependent patients. European Neuropsychopharmacology. 2014;2):S689-S90. | Exclude: Conference abstract of reviewed clinical trial reporting social and functional outcomes |
| 533. Yeo BK. What you need to know: addiction--prescribing naltrexone. Singapore Med J. 1997;38(2):92-3. | Exclude: Review article all references checked |
| 534. Zaaijer ER, Goudriaan AE, Koeter MW, Booij J, van den Brink W. Acceptability of Extended-Release Naltrexone by Heroin-Dependent Patients and Addiction Treatment Providers in the Netherlands. Substance Use & Misuse. 2016;51(14):1905-11. | Exclude: Cross sectional survey of patients and providers of attitudes to XR-NTX |
| 535. Zangiabadian M, Golmohammadi S, Nejadghaderi SA, Zahmatkesh MM, Nasiri MJ, Sadeghian M. The effects of naltrexone on retention in treatment and being opioid-free in opioid-dependent people: A systematic review and meta-analysis. Frontiers in Psychiatry. 2022;13(no pagination). | Exclude: Systematic review all references checked for inclusion |
| 536. Zavod A, Akerman SC, Snow MM, Tierney M, Sullivan MA. Psychoeducational Strategies During Outpatient Transition to Extended-Release Naltrexone for Patients With Opioid Use Disorder. J Am Psychiatr Nurses Assoc. 2019;25(4):272-9. | Exclude: Reports psychoeducational interventions during withdrawal management |
| 537. Zhang P, Tossone K, Ashmead R, Bickert T, Bailey E, Doogan NJ, et al. Examining differences in retention on medication for opioid use disorder: An analysis of Ohio Medicaid data. Journal of Substance Abuse Treatment. 2022;136(no pagination). | Exclude: Reports adjusted hazard ratios of discontinuation at three, six and 12 months but no numbers of people discontinuing at these points unable with data presented to calculate rate of discontinuation for naltrexone. |
| 538. Ziaadini H, Parvaresh N, Afshar N, Hoseinian SM, Sarhadi R, Hahdoost AA. Comparison of the outcomes of three detoxification methods (Clonidin, methadon, rapid) in opioid-dependents referred to kerman shaheed beheshti hospital in a 6 -month follow- up. [Persian, English]. Journal of Kerman University of Medical Sciences. 2011;18(3):246-59. | Exclude: Does not report denominator for number of people initiating naltrexone in each of the three arms to allow calculation of discontinuation rate |

**Table S2: Characteristics of included studies**

| Study ID | Years of data collection | Country | Study Design | Setting | Type of opioid used prior to withdrawal | Unique features of population | Type of detoxification | Naltrexone formulation | Dose | Mean age | % Female | % Employed | n | Initiation/ Discontinuation | Total Newcastle -Ottawa Quality Score |
| --- | --- | --- | --- | --- | --- | --- | --- | --- | --- | --- | --- | --- | --- | --- | --- |
| Wenzel 2024 (3) | 2019 - 2021 | USA | Clinical trial | Outpatient Drug Service |  | No | Inpatient | Depot |  |  |  |  | 21 | Discontinuation | 3 |
| Shulman 2024 (4) | 2021 - 2022 | USA | Clinical trial | Outpatient Drug Service | > 2/3 heroin/ fentanyl use | No | Inpatient | Depot | 380mg |  |  |  | 209 | Discontinuation | 3 |
| Larochelle 2024 (5) | 2014 - 2016 | USA | Retrospective cohort | Outpatient Drug Service |  | No | Inpatient | Depot | 380mg |  | 27 |  | 57201 | Initiation | 3 |
|  |  |  |  |  |  |  |  |  |  |  |  |  | 1645 | Discontinuation | 3 |
| Brenna 2024 (6) | 2018 - 2020 | Norway | Prospective cohort | Outpatient Drug Service |  | No | Inpatient | Depot | 380mg |  |  |  | 138 | Discontinuation | 3 |
| Noam 2023 (7) | 2018 - 2019 | USA | Retrospective cohort | Specialist Inpatient Unit |  | Medicaid | Inpatient | Not specified |  |  | 26.8 |  | 2332 | Initiation | 6 |
| Shulman 2022 (8) | 2015 - 2017 | USA | Prospective cohort | Outpatient Drug Service | Excluded all those on OAT | Previously failed XR-NTX induction | Inpatient | Depot |  |  |  |  | 8 | Discontinuation | 3 |
| Karlsson 2022 (9) | 2018 - 2020 | Norway | Prospective cohort | Outpatient Drug Service | All enrolled in OAT program | No | Inpatient | Depot | 380mg |  |  |  | 135 | Discontinuation | 3 |
| Erdoğan 2022 (10) | 2016 - 2020 | Turkey | Retrospective cohort | Outpatient Drug Service |  | No |  | Implant | 1000mg | 25.5 | 6.7 |  | 208 | Discontinuation | 4 |
| Coleman 2022 (11) | 2018 - 2020 | USA | Retrospective cohort | Outpatient Drug Service | Excluded all those on OAT | Medicaid | Inpatient | Depot |  | 34.7 | 34 |  | 47 | Initiation | 3 |
| Woody 2021 (12) | 2016 - 2018 | USA | Clinical trial | Outpatient Drug Service |  | Criminal Justice Offenders | Inpatient | Depot | 380mg |  |  |  | 54 | Discontinuation | 4 |
| Morgan 2021 (13) | 2017 - 2018 | USA | Retrospective cohort | Outpatient Drug Service |  | Commercially insured |  | Depot | 380mg |  | 39 |  | 1173 | Discontinuation | 4 |
| Mitchell 2021 (14) | 2013 - 2018 | USA | Clinical trial | Outpatient Drug Service |  | Adolescents and Young Adults | Inpatient | Depot | 380mg | 19.2 | 52.4 |  | 82 | Discontinuation | 6 |
| Wang 2020 (15) | 2011 - 2013 | USA | Prospective cohort |  | Heroin or prescription opioid use | Tobacco smokers |  | Depot | 380mg |  |  |  | 58 | Discontinuation | 4 |
| Walley 2020 (16) | 2012 - 2014 | USA | Retrospective cohort | Outpatient Drug Service | 16% on OAT | No |  | Oral or Depot |  |  | 32 |  | 61819 | Initiation | 6 |
| Towers 2020 (17) | 2017 - 2018 | USA | Prospective cohort | Outpatient Drug Service | All enrolled in OAT program | Pregnant women | Inpatient/Outpatient | Oral | 50mg |  |  |  | 137 | Initiation | 3 |
| Makarenko 2019 (18) | 2015 - 2016 | Ukraine | Prospective cohort | Outpatient Drug Service |  | No | Inpatient | Depot | 380mg | 17.9 | 32 | 72 | 134 | Discontinuation | 6 |
| Latif 2019 (19) | 2012 - 2015 | Norway | Clinical trial | Outpatient Drug Service |  | No |  | Depot | 380mg |  |  |  | 71 | Discontinuation | 4 |
| Jarvis 2019 (20) | 2012 - 2016 | USA | Clinical trial | Outpatient Drug Service | Heroin use | No |  | Depot |  |  |  |  | 46 | Discontinuation | 4 |
| Vo 2018 (21) | 2014 - 2017 | USA | Retrospective cohort | Outpatient Drug Service |  | Adolescents and Young Adults | Inpatient | Depot |  |  |  |  | 35 | Discontinuation | 3 |
| Springer 2018 (22) | 2010 - 2016 | USA | Clinical trial | Outpatient Drug Service | All enrolled in OAT program | HIV +ve/Criminal Justice Offenders |  | Depot | 380mg |  |  |  | 45 | Discontinuation | 3 |
| Solli 2018 (23) | 2012 - 2015 | Norway | Prospective cohort | Outpatient Drug Service |  | Previously Tanum 2017 Trial participants | Inpatient | Depot | 380mg | 35.1 | 28.6 |  | 63 | Discontinuation | 3 |
| Shi 2018 (24) | 2012 - 2014 | USA | Prospective cohort |  | Illicit opioid use | No |  | Depot | 380mg | 30.2 | 37.5 |  | 24 | Discontinuation | 2 |
| Saxon 2018 (25) | 2011 - 2013 | USA | Retrospective cohort | Outpatient Drug Service |  | No |  | Depot | 380mg | 29 | 28.4 | 41.4 | 395 | Discontinuation | 3 |
| Rudolf 2018 (26) | 2011 - 2012 | USA | Retrospective cohort | Specialist Inpatient Unit | Excluded all those on OAT | No | Inpatient | Depot |  |  |  |  | 79 | Initiation | 4 |
| Morgan 2018a (27) | 2010 - 2014 | USA | Retrospective cohort | Outpatient Drug Service |  | Commercially insured |  | Depot |  |  | 34 |  | 1343 | Discontinuation | 3 |
| Morgan 2018b (27) | 2010 - 2014 | USA | Retrospective cohort | Outpatient Drug Service |  | Commercially insured |  | Oral |  |  | 40 |  | 5327 | Discontinuation | 3 |
| Lincoln 2018 (28) | 2013 - 2014 | USA | Prospective cohort | Outpatient Drug Service | Excluded all those on OAT | Criminal Justice Offenders |  | Depot | 380mg |  |  |  | 54 | Discontinuation | 4 |
| Lee 2018 (29) | 2014 - 2016 | USA | Clinical trial | Outpatient Drug Service | 94% used heroin or prescription opioids | No | Inpatient | Depot | 380mg |  |  |  | 204 | Discontinuation | 4 |
| Friedmann 2018 (30) | 2012 - 2014 | USA | Clinical trial | Outpatient Drug Service | Excluded all those on OAT | Criminal Justice Offenders |  | Depot |  |  |  |  | 13 | Discontinuation | 2 |
| Chang 2018 (31) | 2014 - 2015 | USA | Retrospective cohort | Outpatient Drug Service |  | Veterans |  | Depot |  | 34.9 | 3 | 27 | 85 | Discontinuation | 3 |
| Bisaga 2018 (32) | 2015 - 2017 | USA | Clinical trial | Outpatient Drug Service | Excluded all those on OAT | No | Outpatient | Depot |  |  |  |  | 166 | Discontinuation | 4 |
| Tanum 2017 (33) | 2012 - 2015 | Norway | Clinical trial | Outpatient Drug Service | Heroin use | No | Inpatient | Depot | 380mg |  |  |  | 71 | Discontinuation | 4 |
| Sullivan 2017 (34) | 2011 - 2015 | USA | Clinical trial | Outpatient Drug Service | Excluded all those on OAT | No | Outpatient | Depot | 380mg |  |  |  | 72 | Discontinuation | 4 |
| Mishlen 2017 (35) |  | USA | Prospective cohort |  | 49% prescription opioid use |  | Outpatient | Depot |  | 34 | 14 |  | 86 | Discontinuation | 6 |
| Korthuis 2017 (36) | 2014 - 2015 | Canada/USA | Clinical trial | Outpatient HIV Service | Excluded all those on OAT | HIV +ve |  | Depot | 380mg |  |  |  | 5 | Discontinuation | 2 |
| Earley 2017 (37) | 2009 - 2012 | USA | Prospective cohort | Outpatient Drug Service | Heroin or prescription opioid use | Healthcare Professionals |  | Depot | 380mg | 42.4 | 81.6 | 42.1 | 38 | Discontinuation | 4 |
| Barnett 2017 (38) | 2015 - 2016 | USA | Retrospective cohort | Outpatient Drug Service |  | No |  | Depot | 380mg |  |  |  | 10 | Discontinuation | 2 |
| Vo 2016 (39) | 2013 - 2015 | USA | Retrospective cohort | Outpatient Drug Service | 85% heroin use | Adolescents and Young Adults | Inpatient/Outpatient | Depot | 380mg | 23.1 | 30 | 35 | 56 | Initiation | 3 |
|  |  |  |  |  |  |  |  |  |  | 22.8 | 12 | 23 | 13 | Discontinuation | 3 |
| Stein 2016 (40) | 2013 - 2015 | USA | Retrospective cohort | Outpatient Drug Service | 82.3% intravenous opioid use | No | Inpatient | Depot | 380mg | 32.4 | 32.3 |  | 62 | Discontinuation | 6 |
| Sajid 2016 (41) | 2012 - 2013 | USA | Retrospective cohort | Outpatient Drug Service | Heroin and prescription opioid use | No |  | Depot | 380mg |  |  |  | 32 | Discontinuation | 3 |
| Merlo 2016 (42) | 1995 - 2001 | USA | Retrospective cohort | Outpatient Drug Service | Excluded all those on OAT | Healthcare Professionals |  | Not specified |  | 43 | 13 |  | 339 | Initiation | 3 |
| Lee 2016 (43) | 2009 - 2013 | USA | Clinical trial | Outpatient Drug Service | 21.1% heroin use | Criminal Justice Offenders |  | Depot | 380mg |  |  |  | 146 | Discontinuation | 4 |
| Dayal 2016 (44) | 2012 | India | Prospective cohort | Outpatient Drug Service | Excluded all those on OAT | No | Inpatient | Oral |  |  |  |  | 225 | Initiation | 4 |
|  |  |  |  |  |  |  |  |  |  | 31.7 | 0.7 | 70.7 | 140 | Discontinuation | 6 |
| Cousins 2016 (45) | 2010 - 2013 | USA | Prospective cohort | Outpatient Drug Service | Heorin or other opiates | No |  | Depot |  | 36.1 | 46.2 | 5.8 | 171 | Discontinuation | 6 |
| Gordon 2015 (46) | 2012 - 2014 | USA | Prospective cohort | Community | Heroin use | Criminal Justice Offenders |  | Depot | 380mg | 39.9 | 40.7 |  | 27 | Discontinuation | 6 |
| Gandhi 2015 (47) |  | India | Prospective cohort |  | All enrolled in OAT program | No | Outpatient | Implant |  |  |  |  | 18 | Discontinuation | 3 |
| Salimi 2014 (48) |  | Iran | Prospective cohort | Community | Heroin, opium or buprenorphine *‘abuse’* | No | Rapid | Oral | 50mg | 30.3 | 3 | 66 | 400 | Discontinuation | 4 |
| Jhugroo 2014 (49) | 2007 - 2010 | Mauritius | Prospective cohort | Outpatient Drug Service | Illicit buprenorphine use | No |  | Implant | 2200mg |  |  |  | 24 | Discontinuation | 4 |
| Dunn 2013a (50) | 2006 - 2009 | USA | Clinical trial | Therapeutic workplace | Injection opiate use | Unemployed |  | Oral | 100mg M, 100mg W, 150mg F | 43.1 | 46 | 0 | 35 | Discontinuation | 3 |
| Dunn 2013b (50) | 2006 - 2009 | USA | Clinical trial | Therapeutic workplace | Injection opiate use | Unemployed |  | Oral | 50mg | 46.8 | 31 | 0 | 32 | Discontinuation | 3 |
| DeFulio 2012a (51) | 2008 - 2009 | USA | Clinical trial | Therapeutic workplace | Heroin use | Unemployed |  | Depot | 380mg | 45 | 26 | 0 | 16 | Discontinuation | 3 |
| DeFulio 2012b (51) | 2008 - 2009 | USA | Clinical trial | Therapeutic workplace | Heroin use | Unemployed |  | Depot | 380mg | 42 | 58 | 0 | 19 | Discontinuation | 3 |
| Chaudhry 2012 (52) | 1997 - 2006 | UK | Prospective cohort | Outpatient Drug Service |  | No | Inpatient/Outpatient | Oral |  | 26.4 | 6.3 |  | 142 | Discontinuation | 4 |
| Krupitsky 2011 (53) | 2008 - 2009 | Russia | Clinical trial | Outpatient drug service | Excluded all those on OAT | No | Inpatient | Depot | 380mg | 29.4 | 10 |  | 126 | Discontinuation | 4 |
| Everly 2011a (54) | 2006 - 2008 | USA | Clinical trial | Therapeutic workplace | Heroin use | Unemployed |  | Depot | 380mg |  |  | 0 | 18 | Discontinuation | 2 |
| Everly 2011b (54) | 2006 - 2008 | USA | Clinical trial | Therapeutic workplace | Heroin use | Unemployed |  | Depot | 380mg |  |  | 0 | 11 | Discontinuation | 2 |
| Day 2011 (55) | 2001 - 2003 | UK | Clinical trial | Specialist Inpatient Unit | All enrolled in OAT program | No | Inpatient/Outpatient | Oral | 50mg |  |  |  | 30 | Initiation | 3 |
|  |  |  |  |  |  |  |  |  |  |  |  |  | 22 | Discontinuation | 3 |
| Kunøe 2010 (56) | 2005 - 2007 | Norway | Clinical trial | Community | Excluded all those on OAT | No/Criminal Justice Offenders | Inpatient | Implant | 2200mg | 33.3 | 23 |  | 61 | Discontinuation | 4 |
| Fishman 2010 (57) | 2007 - 2008 | USA | Prospective cohort | Outpatient Drug Service | Heroin or prescription opioid use | Adolescents and Young Adults | Inpatient | Depot | 380mg | 18.5 | 50 |  | 19 | Discontinuation | 3 |
| Mistral 2008 (58) | 2005 - 2006 | UK | Retrospective cohort | Outpatient Drug Service | Heroin use | Criminal Justice Offenders |  | Oral |  |  | 13.7 |  | 51 | Discontinuation | 3 |
| Mannelli 2007 (59) |  | USA | Clinical trial | Outpatient Drug Service |  | No | Inpatient | Oral | 1 up to 10mg | 32.8 | 32.7 | 19.8 | 435 | Initiation | 4 |
|  |  |  |  |  |  |  |  |  |  |  |  |  | 162 | Discontinuation | 4 |
| Davison 2006 (60) | 1998 - 2000 | USA | Retrospective cohort | Outpatient Drug Service | Heroin use | No | Inpatient | Oral |  |  |  |  | 85 | Initiation | 4 |
|  |  |  |  |  |  |  |  |  |  |  |  |  | 50 | Discontinuation | 4 |
| Marsch 2005 (61) |  | USA | Clinical trial | Outpatient Drug Service | 52.5% heroin use | Adolescents and Young Adults | Outpatient | Oral |  |  |  |  | 21 | Initiation | 2 |
| Hulse 2005 (62) | 2001 - 2002 | Australia | Retrospective cohort | Outpatient Drug Service | Heroin use | No |  | Implant | 3400mg |  |  |  | 384 | Discontinuation | 4 |
| Digiusto 2005a (63) | 1998 | Australia | Clinical trial | Specialist Inpatient Unit | Heroin use | No | Rapid | Oral | 50mg |  |  |  | 68 | Initiation | 3 |
| Digiusto 2005b (63) |  | Australia | Clinical trial | Specialist Inpatient Unit | Heroin use | No | Inpatient | Oral | 50mg |  |  |  | 12 | Initiation | 3 |
| Digiusto 2005c (63) | 2000 - 2001 | Australia | Clinical trial | Specialist Inpatient Unit | Heroin use | No | Outpatient | Oral | 50mg |  |  |  | 21 | Initiation | 3 |
| De Jong 2005a (64) | 1999 - 2001 | Netherlands | Clinical trial | Outpatient Drug Service |  | No | Inpatient | Oral | 50mg |  |  |  | 134 | Discontinuation | 4 |
| De Jong 2005b (64) | 2000 - 2001 | Netherlands | Clinical trial | Outpatient Drug Service |  | No | Rapid | Oral | 50mg |  |  |  | 130 | Discontinuation | 4 |
| Arnold-Reed 2005a (65) | 2000 - 2001 | Australia | Clinical trial | Specialist Inpatient Unit | Heroin use | No | Inpatient/Outpatient | Oral |  |  |  |  | 11 | Discontinuation | 2 |
| Arnold-Reed 2005b (65) | 2001 - 2001 | Australia | Clinical trial | Specialist Inpatient Unit | Heroin use | No | Rapid | Oral |  |  |  |  | 36 | Discontinuation | 4 |
| Tucker 2004 (66) |  | Australia | Clinical trial | Outpatient Drug Service | Heroin use | No |  | Oral | 50mg |  |  |  | 108 | Initiation | 6 |
|  |  |  |  |  |  |  |  |  |  | 29.9 | 36.1 | 47.5 | 97 | Discontinuation | 6 |
| Rea 2004 (67) | 1999 - 2001 | Australia | Clinical trial | Outpatient Drug Service | Heroin use | No | Inpatient/Outpatient | Oral | 50mg/0.5mg/0.05mg | 28.5 | 41 | 20 | 66 | Discontinuation | 6 |
| Malhotra 2003 (68) | 1999 - 2000 | India | Prospective cohort | Outpatient Drug Service | 35% heroin use | No |  | Oral | 50mg | 28.4 | 0 | 72 | 106 | Discontinuation | 6 |
| Foster 2003 (69) | 1997 - 2000 | UK | Prospective cohort | Outpatient Drug Service |  | No |  | Implant | 1000mg | 28.7 | 20.7 |  | 101 | Discontinuation | 4 |
| Summers 2002 (70) |  | UK | Prospective cohort | Outpatient Drug Service |  | No | Self | Oral |  |  |  |  | 10 | Discontinuation | 2 |
| Rabinowitz 2002 (71) |  | Israel | Clinical trial | Specialist Inpatient Unit |  | No | Rapid | Oral |  | 34.7 | 8.3 |  | 22 | Discontinuation | 2 |
| Glasgow 2001 (72) | 1999 | Australia | Prospective cohort | Outpatient Drug Service | All enrolled in OAT program | No | Rapid | Oral |  | 34 | 28.6 | 85.8 | 14 | Discontinuation | 2 |
| Gerra 2001 (73) |  | Italy | Clinical trial | Specialist Inpatient Unit | Heroin use | No | Inpatient | Oral | 50mg |  |  |  | 35 | Initiation | 2 |
| Hulse 2000 (74) | 1997 - 1998 | Australia | Prospective cohort | Outpatient Drug Service | Heroin use | No | Outpatient | Oral | 50mg | 25.9 | 45.3 |  | 300 | Discontinuation | 4 |
| Gerra 2000 (75) |  | Italy | Clinical trial | Specialist Inpatient Unit | Heroin use | No | Inpatient | Oral | 50mg |  | 27.6 |  | 98 | Initiation | 4 |
| Preston 1999a (76) |  | USA | Clinical trial | Outpatient Drug Service | Heroin use | No | Inpatient | Oral | 100mg M, 100mg W, 150mg F | 34.7 | 42 |  | 19 | Discontinuation | 3 |
| Preston 1999b (76) |  | USA | Clinical trial | Outpatient Drug Service | Heroin use | No | Inpatient | Oral | 100mg M, 100mg W, 150mg F | 32.8 | 34.2 |  | 38 | Discontinuation | 3 |
| Bell 1999 (77) | 1998 | Australia | Prospective cohort | Outpatient Drug Service | Mixed: 50% heroin use + 50% OAT methadone | No | Rapid | Oral | 50mg |  |  |  | 24 | Discontinuation | 3 |
| Scherbaum 1998 (78) |  | Germany | Prospective cohort | Specialist Inpatient Unit | Mixed: Opiate “*addicts*” and OAT methadone | No | Rapid | Oral | 50mg | 29.4 | 31.8 |  | 22 | Initiation | 2 |
| Roth 1997 (79) |  | USA | Retrospective cohort | Outpatient Drug Service |  | Healthcare Professionals | Inpatient/Self | Oral | 100mg M, 100mg W, 150mg F | 34.4 | 85 |  | 20 | Discontinuation | 2 |
| Rabinowitz 1997 (80) | 1994 - 1995 | Israel | Retrospective cohort | Outpatient Drug Service |  | No | Rapid | Oral | 25-50mg | 32.9 |  |  | 83 | Discontinuation | 3 |
| Cornish 1997 (81) |  | USA | Clinical trial | Outpatient Drug Service | “*Mostly*” heroin use | Criminal Justice Offenders |  | Oral | 100mg T, 150mg F |  |  |  | 34 | Discontinuation | 3 |
| Azatian 1994 (82) |  | USSR | Prospective cohort | Outpatient/Inpatient Drug Service | *“Majority*” heroin use | No | Inpatient/Self/Outpatient | Oral | 50mg or 100mg M, 100mg W, 150mg F | 29.7 | 11 | 25 | 27 | Discontinuation | 3 |
| Schifano 1990 (83) | 1987 - 1989 | Italy | Retrospective cohort | Outpatient Drug Service | Heroin use | No | Inpatient/Outpatient | Oral | 100mg M, 100mg W, 150mg F | 26.1 | 34.5 | 81 | 84 | Discontinuation | 4 |
| Kosten 1989 (84) |  | USA | Prospective cohort | Specialist Inpatient Unit | Buprenorphine OAT | No | Inpatient | Oral | 50mg |  |  |  | 5 | Discontinuation | 2 |
| Fram 1989 (85) |  | USA | Retrospective cohort | Outpatient Drug Service | Excluded those in OAT program | No | Inpatient | Oral |  | 33.7 | 20 | 80 | 15 | Discontinuation | 2 |
| Vining 1988 (86) |  | USA | Prospective cohort | Outpatient Drug Service | Heroin use | No | Outpatient | Oral | 50mg |  |  |  | 14 | Discontinuation | 2 |
| Capone 1986 (87) | 1982 | USA | Retrospective cohort | Outpatient Drug Service | 70% not enrolled in OAT program | No |  | Oral | 350mg each week in two divided doses | 30.1 | 0 |  | 50 | Discontinuation | 3 |
| Washton 1984 (88) | 1979 - 1981 | USA | Retrospective cohort | Outpatient Drug Service |  | Business Executives/  Physicians | Inpatient | Oral |  | 30.9 | 7 |  | 129 | Discontinuation | 6 |
| Tennant 1984 (89) |  | USA | Retrospective cohort | Outpatient Drug Service | 71.6% heroin use | No | Inpatient/Outpatient/Self | Oral | 100mg M, 100mg W, 150mg F |  | 18.7 | 63.8 | 160 | Discontinuation | 3 |
| Ling 1984 (90) | 1980 - 1983 | USA | Retrospective cohort | Private physicians offices | All “*abused*” at least one (non-OAT) opioid | Healthcare Professionals |  | Oral | 300 - 350mg per week |  | 20 |  | 60 | Discontinuation | 3 |
| Greenstein 1984 (91) | 1981 - 1983 | USA | Retrospective cohort | Outpatient Drug Service | Mixed: Methadone OAT or “*street opiates*” | Veterans | Inpatient | Oral | 100mg M, 100mg W, 150mg F |  |  |  | 85 | Discontinuation | 6 |
| Resnick 1981a (92) |  | USA | Clinical trial | Outpatient Drug Service | Mixed: Methadone OAT or illicit opioids | No | Inpatient | Oral | 100mg M, 100mg W, 150mg F |  |  |  | 16 | Discontinuation | 3 |
| Resnick 1981b (92) |  | USA | Clinical trial | Outpatient Drug Service | Mixed: Methadone OAT or illicit opioids | No | Inpatient | Oral | 100mg M, 100mg W, 150mg F |  |  |  | 18 | Discontinuation | 3 |
| Greenstein 1981 (93) | 1973 - 1979 | USA | Retrospective cohort | Outpatient Drug Service | Mixed: Methadone OAT or illicit opioids | Veterans | Inpatient | Oral | 100mg M, 100mg W, 150mg F | 28.1 | 1.2 |  | 242 | Discontinuation | 6 |
| Anton 1981a (94) | 1978 - 1980 | USA | Retrospective cohort | Outpatient Drug Service |  | No | Inpatient/Self | Oral | 100mg M, 100mg W, 150mg F |  | 15 | 27.5 | 25 | Discontinuation | 4 |
| Anton 1981b (94) | 1978 - 1980 | USA | Retrospective cohort | Outpatient Drug Service |  | No | Inpatient/Self | Oral | 100mg M, 100mg W, 150mg F |  | 4 | 64 | 40 | Discontinuation | 2 |
| Grabowski 1979 (95) |  | USA | Retrospective cohort | Outpatient Drug Service | Mixed: Methadone OAT or heroin use | No |  | Oral | 100mg - 125mg three times per week |  |  |  | 125 | Discontinuation | 3 |
| Resnick 1978 (96) | 1975 - 1977 | USA | Retrospective cohort | Outpatient Drug Service |  | No | Inpatient | Oral | 100mg M, 100mg W, 150mg F |  | 0 |  | 285 | Discontinuation | 4 |
| Lewis 1978 (97) | 1974 - 1976 | USA | Retrospective cohort | Outpatient Drug Service |  | No | Inpatient | Oral | 50mg |  | 0 |  | 738 | Initiation | 4 |
|  |  |  |  |  |  |  |  |  |  | 27.7 | 0 | 14 | 22 | Discontinuation | 4 |
| Hollister 1977 (98) |  | USA | Clinical trial | Outpatient Drug Service | Opiate use “*Street addicts*” | Veterans | Inpatient | Oral | 50mg/100mg M, 100mg W, 150mg F | 29.2 | 0 |  | 108 | Initiation | 4 |
|  |  |  |  |  |  |  |  |  |  | 28.8 | 0 |  | 73 | Discontinuation | 4 |
| Meyer 1976 (99) |  | USA | Retrospective cohort | Community |  | No | Inpatient | Oral | 50 or 75 mg |  |  |  | 40 | Discontinuation | 3 |

**Table S3: GRADE clinical evidence profile for overall estimates of the proportion of individuals initiating naltrexone**

| **Certainty Assessment** | | | | | | **Proportion %  (95% CI)** | **Certainty** |
| --- | --- | --- | --- | --- | --- | --- | --- |
| **No of prevalence estimates** | **Risk of bias** | **Inconsistency** | **Indirectness** | **Imprecision** | **Publication Bias** |  |  |
| Oral | | | | | | | |
| 15 | Very serious | Very  serious | None | Very  serious | Serious | 18.2  (2.7 - 42.5) | VERY LOW |
| Depot | | | | | | | |
| 4 | Very serious | Very  serious | None | Serious | N/A | 60.3  (33.5 - 44.1) | VERY LOW |

**Table S4: GRADE clinical evidence profile for overall estimates of the proportion of individuals discontinuing naltrexone at one month following initiation**

| **Certainty Assessment** | | | | | | **Proportion %  (95% CI)** | **Certainty** |
| --- | --- | --- | --- | --- | --- | --- | --- |
| **No of prevalence estimates** | **Risk of bias** | **Inconsistency** | **Indirectness** | **Imprecision** | **Publication Bias** |  |  |
| Oral | | | | | | | |
| 34 | Very serious | Very  serious | None | Serious | Serious | 50.0  (41.9 - 58.1) | VERY LOW |
| Depot | | | | | | | |
| 33 | Very serious | Very  serious | None | Serious | Serious | 26.1  (19.5 - 33.3) | VERY LOW |
| Implant | | | | | | | |
| 2 | Very serious | N/A | None | None | N/A | 7.8  (5.5 - 10.4) | VERY LOW |

**Table S5: GRADE clinical evidence profile for overall estimates of the proportion of individuals discontinuing naltrexone at three months following initiation**

| **Certainty Assessment** | | | | | | **Proportion %  (95% CI)** | **Certainty** |
| --- | --- | --- | --- | --- | --- | --- | --- |
| **No of prevalence estimates** | **Risk of bias** | **Inconsistency** | **Indirectness** | **Imprecision** | **Publication Bias** |  |  |
| Oral | | | | | | | |
| 29 | Very serious | Very  serious | None | Very  serious | None | 61.3  (50.9 - 71.2) | VERY LOW |
| Depot | | | | | | | |
| 33 | Very serious | Very  serious | None | Serious | Serious | 46.7  (38.4 - 55.1) | VERY LOW |
| Implant | | | | | | | |
| 3 | Very serious | N/A | None | Very serious | N/A | 30.0  (0.0 - 79.2) | VERY LOW |

**Table S6: GRADE clinical evidence profile for overall estimates of the proportion of individuals discontinuing naltrexone at six months following initiation**

| **Certainty Assessment** | | | | | | **Proportion %  (95% CI)** | **Certainty** |
| --- | --- | --- | --- | --- | --- | --- | --- |
| **No of prevalence estimates** | **Risk of bias** | **Inconsistency** | **Indirectness** | **Imprecision** | **Publication Bias** |  |  |
| Oral | | | | | | | |
| 19 | Very serious | Very  serious | None | Very  serious | None | 71.0  (57.3 - 83.0) | VERY LOW |
| Depot | | | | | | | |
| 22 | Very serious | Very  serious | None | Very serious | Serious | 60.0  (43.2 - 75.8) | VERY LOW |
| Implant | | | | | | | |
| 3 | Very serious | Very  serious | None | Very serious | N/A | 76.1  (47.6 - 95.9) | VERY LOW |

**Table S7: Univariable meta-regression of individual and study-level factors associated with the proportion of individuals discontinuing oral naltrexone as a relapse prevention pharmacotherapy in opioid use disorder at three months following initiation**

|  | **Proportion estimates (n)** | **Individuals (n)** | **Proportion** | **LCI** | **UCI** | **τ^2^** | **I^2^ (%)** |  | |
| --- | --- | --- | --- | --- | --- | --- | --- | --- | --- |
|  | 29 | 2,409 | 61.3 | 50.9 | 71.2 | 0.290 | 95.9 |  |  |
|  | **Proportion estimates (n)** | **Individuals (n)** | **Beta** | **LCI** | **UCI** | **τ^2^** | **I^2^ (%)** | ***p* value** | **Adjusted *R*^2^ (%)** |
| **Univariable** |  | | | | | | | | |
| Mean age of individuals (Continuous) | 17 | 1,571 | -0.01 | -0.03 | 0.02 | 0.041 | 84.5 | 0.511 | 3.9 |
| Proportion of female individuals (Continuous) | 21 | 2,049 | -0.003 | -0.01 | 0.002 | 0.039 | 85.0 | 0.216 | 0.1 |
| Proportion of employed individuals (Continuous) | 13 | 1,164 | -0.002 | -0.01 | 0.004 | 0.051 | 85.2 | 0.387 | 0.7 |
| Median year of data collection (Continuous) | 16 | 1,357 | -0.01 | -0.01 | 0.002 | 0.009 | 40.5 | 0.144 | 34.8 |
| Total Newcastle-Ottawa scale quality score (Continuous) | 29 | 2,409 | 0.01 | -0.06 | 0.09 | 0.037 | 80.7 | 0.683 | 5.5 |
| Study setting (Binary: Routine vs investigational (ref)) | 29 | 2,409 | 0.11 | -0.07 | 0.28 | 0.032 | 73.7 | 0.229 | 8.3 |
| Type of opioid used prior to withdrawal and naltrexone initiation (Binary: OAT vs. Extra-medical/illicit use (ref)) | 12 | 1,082 | -0.06 | -0.86 | 0.75 | 0.054 | 88.4 | 0.880 | 7.0 |
| Were the recruited individuals healthcare professionals? (Binary Yes vs No (ref)) | 29 | 2,409 | -0.41 | -0.98 | 0.19 | 0.034 | 80.6 | 0.174 | 2.7 |
| Were the recruited individuals criminal justice offenders? (Binary Yes vs No (ref)) | 29 | 2,409 | 0.04 | -0.33 | 0.40 | 0.036 | 80.9 | 0.843 | 4.4 |
| Was the naltrexone reportedly administered under supervision? (Binary: Yes vs. No (ref)) | 29 | 2,409 | -0.19 | -0.37 | -0.01 | 0.031 | 76.2 | 0.043 | 11.9 |
| Was psychosocial therapy reportedly provided to people on naltrexone (Binary: Yes vs. No (ref)) | 29 | 2,409 | -0.01 | -0.21 | 0.19 | 0.037 | 79.0 | 0.946 | 5.9 |
| Was a specific psychosocial intervention designed to increase naltrexone adherence delivered? (Binary: Yes vs. No (ref)) | 29 | 2,409 | -0.18 | -0.51 | 0.14 | 0.034 | 80.7 | 0.261 | 1.4 |

^LCI Lower 95%Confidence Interval; UCI Upper 95% Confidence interval^

**Table S8: Univariable meta-regression of individual and study-level factors associated with the proportion of individuals discontinuing oral naltrexone as a relapse prevention pharmacotherapy in opioid use disorder at six months following initiation**

|  | **Proportion estimates (n)** | **Individuals (n)** | **Proportion** | **LCI** | **UCI** | **τ^2^** | **I^2^ (%)** |  | |
| --- | --- | --- | --- | --- | --- | --- | --- | --- | --- |
|  | 19 | 1,911 | 71.0 | 57.3 | 83.0 | 0.364 | 97.2 |  |  |
|  | **Proportion estimates (n)** | **Individuals (n)** | **Beta** | **LCI** | **UCI** | **τ^2^** | **I^2^ (%)** | ***p* value** | **Adjusted *R*^2^ (%)** |
| **Univariable** |  | | | | | | | | |
| Mean age of individuals (Continuous) | 13 | 1,626 | -0.004 | -0.03 | 0.02 | 0.055 | 89.6 | 0.737 | 8.0 |
| Proportion of female individuals (Continuous) | 14 | 1,686 | -0.002 | -0.008 | 0.01 | 0.052 | 88.8 | 0.560 | 7.6 |
| Proportion of employed individuals (Continuous) | 7 | 863 | -0.001 | -0.009 | 0.007 | 0.058 | 89.6 | 0.767 | 17.0 |
| Median year of data collection (Continuous) | 12 | 1,266 | -0.001 | --0.01 | 0.1 | 0.044 | 82.7 | 0.842 | 11.9 |
| Total Newcastle-Ottawa scale quality score (Continuous) | 19 | 1,911 | 0.01 | -0.08 | 0.10 | 0.046 | 84.5 | 0.774 | 6.1 |
| Study setting (Binary: Routine vs. investigational (ref)) | 19 | 1,911 | 0.05 | -0.19 | 0.30 | 0.044 | 80.8 | 0.650 | 2.7 |
| Type of opioid used prior to withdrawal and naltrexone initiation (Binary: OAT vs. Extra-medical/illicit use (ref)) | 11 | 1,223 | 0.28 | -0.42 | 0.98 | 0.045 | 84.9 | 0.390 | 0.7 |
| Were the recruited individuals healthcare professionals? (Binary Yes vs No (ref)) | 19 | 1,911 | -0.27 | -0.69 | 0.14 | 0.042 | 85.5 | 0.186 | 2.5 |
| Were the recruited individuals criminal justice offenders? (Binary Yes vs No (ref)) | 19 | 1,911 | -0.21 | -0.79 | 0.38 | 0.045 | 85.7 | 0.463 | 3.1 |
| Was the naltrexone reportedly administered under supervision? (Binary: Yes vs. No (ref)) | 19 | 1,911 | -0.27 | -0.50 | -0.04 | 0.032 | 75.4 | 0.022 | 27.0 |
| Was psychosocial therapy reportedly provided to people on naltrexone (Binary: Yes vs. No (ref)) | 19 | 1,911 | 0.19 | -0.07 | 0.44 | 0.038 | 80.9 | 0.138 | 12.2 |
| Was a specific psychosocial intervention designed to increase naltrexone adherence delivered? (Binary: Yes vs. No (ref)) | 19 | 1,911 | -0.03 | -0.47 | 0.41 | 0.046 | 85.8 | 0.887 | 5.7 |

^LCI Lower 95%Confidence Interval; UCI Upper 95% Confidence interval^

**Table S9: Univariable meta-regression of individual and study-level factors associated with the proportion of individuals discontinuing long-acting injectable depot naltrexone as a relapse prevention pharmacotherapy in opioid use disorder at three months following initiation**

|  | **Proportion estimates (n)** | **Individuals (n)** | **Proportion** | **LCI** | **UCI** | **τ^2^** | **I^2^ (%)** |  | |
| --- | --- | --- | --- | --- | --- | --- | --- | --- | --- |
|  | 33 | 3,459 | 46.7 | 38.4 | 55.1 | 0.193 | 94.7 |  |  |
|  | **Proportion estimates (n)** | **Individuals (n)** | **Beta** | **LCI** | **UCI** | **τ^2^** | **I^2^ (%)** | ***p* value** | **Adjusted *R*^2^ (%)** |
| **Univariable** |  | | | | | | | | |
| Mean age of individuals (Continuous) | 13 | 1,110 | -0.01 | -0.03 | 0.02 | 0.601 | 82.4 | 0.626 | 11.1 |
| Proportion of female individuals (Continuous) | 14 | 2,283 | 0.006 | -0.004 | 0.02 | 0.044 | 79.1 | 0.220 | 8.4 |
| Proportion of employed individuals (Continuous) | 9 | 862 | -0.002 | -0.01 | 0.01 | 0.061 | 80.6 | 0.643 | 8.8 |
| Median year of data collection (Continuous) | 33 | 3,459 | 0.002 | -0.03 | 0.03 | 0.040 | 76.9 | 0.882 | 4.4 |
| Total Newcastle-Ottawa scale quality score (Continuous) | 33 | 3,459 | 0.06 | -0.02 | 0.13 | 0.037 | 76.5 | 0.126 | 3.6 |
| Type of opioid used prior to withdrawal and naltrexone initiation (Binary: OAT vs. Extra-medical/illicit use (ref)) | 20 | 878 | -0.06 | -0.88 | 0.67 | 0.044 | 82.2 | 0.860 | 4.9 |
| Study setting (Binary: Routine vs. investigational (ref)) | 33 | 3,459 | 0.15 | -0.06 | 0.35 | 0.035 | 71.2 | 0.151 | 9.1 |
| Were the recruited individuals healthcare professionals? (Binary Yes vs No (ref)) | 33 | 3,459 | -0.41 | -0.98 | 0.19 | 0.034 | 80.6 | 0.174 | 2.7 |
| Were the recruited individuals criminal justice offenders? (Binary Yes vs No (ref)) | 33 | 3,459 | 0.08 | -0.15 | 0.32 | 0.041 | 76.5 | 0.476 | 5.3 |
| Was psychosocial therapy reportedly provided to people on naltrexone (Binary: Yes vs. No (ref)) | 33 | 3,459 | -0.01 | -0.19 | 0.17 | 0.041 | 76.5 | 0.924 | 4.8 |
| Was a specific psychosocial intervention designed to increase naltrexone adherence delivered? (Binary: Yes vs. No (ref)) | 33 | 3,459 | -0.27 | -0.57 | 0.02 | 0.035 | 74.7 | 0.067 | 10.3 |

^LCI Lower 95%Confidence Interval; UCI Upper 95% Confidence Interval^

**Table S10: Univariable meta-regression of individual and study-level factors associated with the proportion of individuals discontinuing long-acting injectable depot naltrexone as a relapse prevention pharmacotherapy in opioid use disorder at six months following initiation**

|  | **Proportion estimates (n)** | **Individuals (n)** | **Proportion** | **LCI** | **UCI** | **τ^2^** | **I^2^ (%)** |  | |
| --- | --- | --- | --- | --- | --- | --- | --- | --- | --- |
|  | 22 | 3,084 | 60.0 | 43.2 | 75.8 | 0.591 | 98.4 |  |  |
|  | **Proportion estimates (n)** | **Individuals (n)** | **Beta** | **LCI** | **UCI** | **τ^2^** | **I^2^ (%)** | ***p* value** | **Adjusted *R*^2^ (%)** |
| **Univariable** |  | | | | | | | | |
| Mean age of individuals (Continuous) | 11 | 702 | -0.02 | -0.04 | 0.01 | 0.037 | 74.7 | 0.110 | 15.9 |
| Proportion of female individuals (Continuous) | 11 | 702 | -0.001 | -0.01 | 0.01 | 0.051 | 75.8 | 0.897 | 16.1 |
| Proportion of employed individuals (Continuous) | 6 | 342 | -0.04 | -0.03 | 0.02 | 0.063 | 69.1 | 0.609 | 11.6 |
| Median year of data collection (Continuous) | 22 | 3,084 | 0.02 | -0.02 | 0.06 | 0.043 | 88.9 | 0.354 | 0.8 |
| Total Newcastle-Ottawa scale quality score (Continuous) | 22 | 3,084 | 0.07 | -0.03 | 0.16 | 0.040 | 89.4 | 0.159 | 6.2 |
| Study setting (Binary: Routine vs. investigational (ref)) | 22 | 3,084 | 0.22 | -0.01 | 0.45 | 0.032 | 73.4 | 0.062 | 24.3 |
| Type of opioid used prior to withdrawal and naltrexone initiation (Binary: OAT vs. Extra-medical/illicit use (ref)) | 12 | 805 | 0.02 | -0.87 | 0.67 | 0.022 | 80.1 | 0.810 | 7.0 |
| Were the recruited individuals healthcare professionals? (Binary Yes vs No (ref)) | 22 | 3,084 | -0.35 | -0.90 | 0.20 | 0.041 | 89.1 | 0.194 | 4.8 |
| Were the recruited individuals criminal justice offenders? (Binary Yes vs No (ref)) | 22 | 3,084 | -0.01 | -0.29 | 0.27 | 0.045 | 88.2 | 0.934 | 5.7 |
| Was psychosocial therapy reportedly provided to people on naltrexone (Binary: Yes vs. No (ref)) | 22 | 3,084 | -0.10 | -0.36 | 0.17 | 0.044 | 82.6 | 0.465 | 2.4 |
| Was a specific psychosocial intervention designed to increase naltrexone adherence delivered? (Binary: Yes vs. No (ref)) | 22 | 3,084 | -0.11 | -0.58 | 0.35 | 0.044 | 89.5 | 0.613 | 3.0 |

^LCI Lower 95%Confidence Interval; UCI Upper 95% Confidence Interval^

**Table S11: Preferred Reporting Items for Systematic Reviews and Meta-Analyses (PRISMA) Checklist**

|  | **Section/topic** | **#** | **Checklist item** | **Reported on page #** |
| --- | --- | --- | --- | --- |
|  | **TITLE** | | |  |
|  | Title | 1 | Identify the report as a systematic review, meta-analysis, or both. | 1 |
|  | **ABSTRACT** | | |  |
|  | Structured summary | 2 | Provide a structured summary including, as applicable: background; objectives; data sources; study eligibility criteria, participants, and interventions; study appraisal and synthesis methods; results; limitations; conclusions and implications of key findings; systematic review registration number. | 3 |
|  | **INTRODUCTION** | | |  |
|  | Rationale | 3 | Describe the rationale for the review in the context of what is already known. | 5-6 |
|  | Objectives | 4 | Provide an explicit statement of questions being addressed with reference to participants, interventions, comparisons, outcomes, and study design (PICOS). | 6 |
|  | **METHODS** | | |  |
|  | Protocol and registration | 5 | Indicate if a review protocol exists, if and where it can be accessed (e.g., Web address), and, if available, provide registration information including registration number. | 6; OSM |
|  | Eligibility criteria | 6 | Specify study characteristics (e.g., PICOS, length of follow-up) and report characteristics (e.g., years considered, language, publication status) used as criteria for eligibility, giving rationale. | 6-7 |
|  | Information sources | 7 | Describe all information sources (e.g., databases with dates of coverage, contact with study authors to identify additional studies) in the search and date last searched. | 6 |
|  | Search | 8 | Present full electronic search strategy for at least one database, including any limits used, such that it could be repeated. | 6; OSM |
|  | Study selection | 9 | State the process for selecting studies (i.e., screening, eligibility, included in systematic review, and, if applicable, included in the meta-analysis). | 6-7 |
|  | Data collection process | 10 | Describe method of data extraction from reports (e.g., piloted forms, independently, in duplicate) and any processes for obtaining and confirming data from investigators. | 7-8; OSM |
|  | Data items | 11 | List and define all variables for which data were sought (e.g., PICOS, funding sources) and any assumptions and simplifications made. | 7-8 |
|  | Risk of bias in individual studies | 12 | Describe methods used for assessing risk of bias of individual studies (including specification of whether this was done at the study or outcome level), and how this information is to be used in any data synthesis. | 8-9 |
|  | Summary measures | 13 | State the principal summary measures (e.g., risk ratio, difference in means). | 7 |
|  | Synthesis of results | 14 | Describe the methods of handling data and combining results of studies, if done, including measures of consistency (e.g., I^2^) for each meta-analysis. | 9-11 |
|  | Risk of bias across studies | 15 | Specify any assessment of risk of bias that may affect the cumulative evidence (e.g., publication bias, selective reporting within studies). | 8-11 |
|  | Additional analyses | 16 | Describe methods of additional analyses (e.g., sensitivity or subgroup analyses, meta-regression), if done, indicating which were pre-specified. | 10-11 |
| **RESULTS** | | | |  |
| Study selection | | 17 | Give numbers of studies screened, assessed for eligibility, and included in the review, with reasons for exclusions at each stage, ideally with a flow diagram. | 12 |
| Study characteristics | | 18 | For each study, present characteristics for which data were extracted (e.g., study size, PICOS, follow-up period) and provide the citations. | Table One |
| Risk of bias within studies | | 19 | Present data on risk of bias of each study and, if available, any outcome level assessment (see item 12). | Table One; OSM |
| Results of individual studies | | 20 | For all outcomes considered (benefits or harms), present, for each study: (a) simple summary data for each intervention group (b) effect estimates and confidence intervals, ideally with a forest plot. | Figure three and four; OSM |
| Synthesis of results | | 21 | Present results of each meta-analysis done, including confidence intervals and measures of consistency. | 12-14Figure three and four; OSM |
| Risk of bias across studies | | 22 | Present results of any assessment of risk of bias across studies (see Item 15). | 12-14; OSM |
| Additional analysis | | 23 | Give results of additional analyses, if done (e.g., sensitivity or subgroup analyses, meta-regression [see Item 16]). | 14-15 |
| **DISCUSSION** | | | |  |
| Summary of evidence | | 24 | Summarize the main findings including the strength of evidence for each main outcome; consider their relevance to key groups (e.g., healthcare providers, users, and policy makers). | 16 |
| Limitations | | 25 | Discuss limitations at study and outcome level (e.g., risk of bias), and at review-level (e.g., incomplete retrieval of identified research, reporting bias). | 17-18 |
| Conclusions | | 26 | Provide a general interpretation of the results in the context of other evidence, and implications for future research. | 18-20 |
| **FUNDING** | | | |  |
| Funding | | 27 | Describe sources of funding for the systematic review and other support (e.g., supply of data); role of funders for the systematic review. | 2 |

**Table S12: Meta-analysis of Observational Studies in Epidemiology (MOOSE) checklist**

| **Item No** | **Recommendation** | **Reported on Page No** |
| --- | --- | --- |
| Reporting of background should include | | |
| 1 | Problem definition | 6 |
| 2 | Hypothesis statement | 6 |
| 3 | Description of study outcome(s) | 7 |
| 4 | Type of exposure or intervention used | 7 |
| 5 | Type of study designs used | 6-7 |
| 6 | Study population | 6-7 |
| Reporting of search strategy should include | | |
| 7 | Qualifications of searchers (eg, librarians and investigators) | 6 |
| 8 | Search strategy, including time period included in the synthesis and key words | 6; OSM |
| 9 | Effort to include all available studies, including contact with authors | 6-7 |
| 10 | Databases and registries searched | 6 |
| 11 | Search software used, name and version, including special features used (eg, explosion) | 6; OSM |
| 12 | Use of hand searching (eg, reference lists of obtained articles) | 6-7 |
| 13 | List of citations located and those excluded, including justification | 12; OSM |
| 14 | Method of addressing articles published in languages other than English | 6; 17 |
| 15 | Method of handling abstracts and unpublished studies | 7 |
| 16 | Description of any contact with authors | 7 |
| Reporting of methods should include | | |
| 17 | Description of relevance or appropriateness of studies assembled for assessing the hypothesis to be tested | 6-7 |
| 18 | Rationale for the selection and coding of data (eg, sound clinical principles or convenience) | 7-8 |
| 19 | Documentation of how data were classified and coded (eg, multiple raters, blinding and interrater reliability) | 7-8 |
| 20 | Assessment of confounding (eg, comparability of cases and controls in studies where appropriate) | N/A |
| 21 | Assessment of study quality, including blinding of quality assessors, stratification or regression on possible predictors of study results | 8-9 |
| 22 | Assessment of heterogeneity | 10-11 |
| 23 | Description of statistical methods (eg, complete description of fixed or random effects models, justification of whether the chosen models account for predictors of study results, dose-response models, or cumulative meta-analysis) in sufficient detail to be replicated | 10-12 |
| 24 | Provision of appropriate tables and graphics | 33-51 |
| Reporting of results should include | | |
| 25 | Graphic summarizing individual study estimates and overall estimate | 50-51; OSM |
| 26 | Table giving descriptive information for each study included | 45; OSM |
| 27 | Results of sensitivity testing (eg, subgroup analysis) | 14-15; 46-47 |
| 28 | Indication of statistical uncertainty of findings | Throughout; 14-15 |

**References:**

1.	Higgins JPT, Green S (editors). Cochrane Handbook for Systematic Reviews of Interventions Version 5.1.0 [updated March 2011]. The Cochrane Collaboration, 2011. Available from <http://handbook.cochrane.org>.

2.	Egger M., Smith G. D., Schneider M., Minder C. Bias in meta-analysis detected by a simple, graphical test, BMJ 1997: 315: 629.

3.	Wenzel K., Thomas J., Carrano J., Stidham J., Fishman M. A pilot randomized controlled trial of medication adherence therapy: Psychosocial leverage using a significant other (MAT-PLUS) for individuals on extended-release naltrexone, Journal of Substance Use and Addiction Treatment 2024: 163: 209366.

4.	Shulman M., Greiner M. G., Tafessu H. M., Opara O., Ohrtman K., Potter K. et al. Rapid Initiation of Injection Naltrexone for Opioid Use Disorder: A Stepped-Wedge Cluster Randomized Clinical Trial, JAMA Network Open 2024: 7: e249744.

5.	Larochelle M., Yan S., Christine P., Hsu H., Bovell-Ammon B., Puppala R. et al. An Emulated Trial of Buprenorphine-Naloxone Versus Extended-Release Naltrexone Following Medically Managed Opioid Withdrawal, Drug and Alcohol Dependence 2024: Conference: Abstracts from the 2023 Annual Meeting of the College on Problems of Drug Dependence. Downtown Denver Sheraton.

6.	Brenna I. H., Waleur K. M., Benth J. S., Solli K. K., Mordal J., Loberg E. M. et al. Patients with Opioid Use Disorder Choosing Treatment with Extended-Release Naltrexone: A 6-Month Naturalistic Study, European Addiction Research 2024: (no pagination).

7.	Noam K. R., Schmutte T. J., Pirard S., Bourdon C., Langless D., Plant R. Associations Between Inpatient Induction on Medications for Opioid Use Disorder and Postdischarge Medications for Opioid Use Disorder Adherence, Overdose, and Service Use, Journal of Addiction Medicine 2023: 17(3): E199-E201.

8.	Shulman M., Hu M. C., Nunes E. V., Bisaga A. Initiating Long-acting Injectable Naltrexone: Procedure for a Second Attempt After a First Attempt Fails, Journal of Addiction Medicine 2022: 16: 588-591.

9.	Karlsson A. T., Vederhus J. K., Clausen T., Weimand B., Solli K. K., Tanum L. Impact of Impulsivity, Hyperactivity, and Inattention on Discontinuation Rate among Opioid-Dependent Patients Treated with Extended-Release Naltrexone, International Journal of Environmental Research and Public Health 2022: 19(18) (no pagination).

10.	Erdogan A., Topcuoglu M., Coskun M. N., Cinemre B., Kulaksizoglu B., Kuloglu M. M. Comparison of naltrexone implant and oral buprenorphine-naloxone in the treatment of opiate use disorder, Human Psychopharmacology 2022: 37: e2813.

11.	Coleman G. C., Baca-Atlas M., Williams J. B. Real-World Outcomes With MOUD in Uninsured Patients: Inpatient to Outpatient Transitions, Journal of Addiction Medicine 2022: 16(5): e308.

12.	Woody G. E., Poole S., Yu E., Carroll J., Lynch K. G. Extended release injectable naltrexone before vs. after release: A randomized trial of opioid addicted persons who are in prison, Journal of Substance Abuse Treatment 2021: 127: 108355.

13.	Morgan J. R., Walley A. Y., Murphy S. M., Chatterjee A., Hadland S. E., Barocas J. et al. Characterizing initiation, use, and discontinuation of extended-release buprenorphine in a nationally representative United States commercially insured cohort, Drug and Alcohol Dependence 2021: 225(no pagination).

14.	Mitchell S. G., Monico L. B., Gryczynski J., Fishman M. J., O'Grady K. E., Schwartz R. P. Extended-release naltrexone for youth with opioid use disorder, Journal of Substance Abuse Treatment 2021: 130: 108407.

15.	Wang A. L., Shi Z., Elman I., Langleben D. D. Reduced cigarette smoking during injectable extended-release naltrexone treatment for opioid use disorder, American Journal of Drug & Alcohol Abuse 2020: 46: 472-477.

16.	Walley A. Y., Lodi S., Li Y., Bernson D., Babakhanlou-Chase H., Land T. et al. Association between mortality rates and medication and residential treatment after in-patient medically managed opioid withdrawal: a cohort analysis, Addiction 2020: 115: 1496-1508.

17.	Towers C. V., Katz E., Weitz B., Visconti K. Use of naltrexone in treating opioid use disorder in pregnancy, Am J Obstet Gynecol 2020: 222: 83.e81-83.e88.

18.	Makarenko I., Pykalo I., Springer S. A., Mazhnaya A., Marcus R., Filippovich S. et al. Treating opioid dependence with extended-release naltrexone (XR-NTX) in Ukraine: Feasibility and three-month outcomes, Journal of Substance Abuse Treatment 2019: 104: 34-41.

19.	Latif Z. E., Saltyte Benth J., Solli K. K., Opheim A., Kunoe N., Krajci P. et al. Anxiety, Depression, and Insomnia Among Adults With Opioid Dependence Treated With Extended-Release Naltrexone vs Buprenorphine-Naloxone: A Randomized Clinical Trial and Follow-up Study, JAMA Psychiatry 2019: 76: 127-134.

20.	Jarvis B. P., Holtyn A. F., DeFulio A., Koffarnus M. N., Leoutsakos J. S., Umbricht A. et al. The effects of extended-release injectable naltrexone and incentives for opiate abstinence in heroin-dependent adults in a model therapeutic workplace: A randomized trial, Drug & Alcohol Dependence 2019: 197: 220-227.

21.	Vo H. T., Burgower R., Rozenberg I., Fishman M. Home-based delivery of XR-NTX in youth with opioid addiction, Journal of Substance Abuse Treatment 2018: 85: 84-89.

22.	Springer S. A., Di Paola A., Azar M. M., Barbour R., Biondi B. E., Desabrais M. et al. Extended-release naltrexone improves viral suppression among incarcerated persons living with HIV with opioid use disorders transitioning to the community: results of a double-blind, placebo-controlled randomized trial, JAIDS Journal of Acquired Immune Deficiency Syndromes 2018: 78: 43-53.

23.	Solli K. K., Latif Z. E., Opheim A., Krajci P., Sharma-Haase K., Benth J. S. et al. Effectiveness, safety and feasibility of extended-release naltrexone for opioid dependence: a 9-month follow-up to a 3-month randomized trial, Addiction 2018: 113: 1840-1849.

24.	Shi Z., Wang A. L., Jagannathan K., Fairchild V. P., O'Brien C. P., Childress A. R. et al. Effects of extended-release naltrexone on the brain response to drug-related stimuli in patients with opioid use disorder, J Psychiatry Neurosci 2018: 43: 254-261.

25.	Saxon A. J., Akerman S. C., Liu C. C., Sullivan M. A., Silverman B. L., Vocci F. J. Extended-release naltrexone (XR-NTX) for opioid use disorder in clinical practice: Vivitrol's Cost and Treatment Outcomes Registry, Addiction 2018: 113: 1477-1487.

26.	Rudolf G., Walsh J., Plawman A., Gianutsos P., Alto W., Mancl L. et al. A novel non-opioid protocol for medically supervised opioid withdrawal and transition to antagonist treatment, American Journal of Drug & Alcohol Abuse 2018: 44: 302-309.

27.	Morgan J. R., Schackman B. R., Leff J. A., Linas B. P., Walley A. Y. Injectable naltrexone, oral naltrexone, and buprenorphine utilization and discontinuation among individuals treated for opioid use disorder in a United States commercially insured population, Journal of Substance Abuse Treatment 2018: 85: 90-96.

28.	Lincoln T., Johnson B. D., McCarthy P., Alexander E. Extended-release naltrexone for opioid use disorder started during or following incarceration, Journal of Substance Abuse Treatment 2018: 85: 97-100.

29.	Lee J. D., Nunes E. V., Jr., Novo P., Bachrach K., Bailey G. L., Bhatt S. et al. Comparative effectiveness of extended-release naltrexone versus buprenorphine-naloxone for opioid relapse prevention (X:BOT): a multicentre, open-label, randomised controlled trial, Lancet 2018: 391: 309-318.

30.	Friedmann P. D., Wilson D., Hoskinson R., Jr., Poshkus M., Clarke J. G. Initiation of extended release naltrexone (XR-NTX) for opioid use disorder prior to release from prison, Journal of Substance Abuse Treatment 2018: 85: 45-48.

31.	Chang G., Crawford M., Pitts M., Schein A. Z., Goodwin K., Enggasser J. L. Adherence to extended release naltrexone: Patient and treatment characteristics, American Journal on Addictions 2018: 27: 524-530.

32.	Bisaga A., Mannelli P., Yu M., Nangia N., Graham C. E., Tompkins D. A. et al. Outpatient transition to extended-release injectable naltrexone for patients with opioid use disorder: A phase 3 randomized trial, Drug & Alcohol Dependence 2018: 187: 171-178.

33.	Tanum L., Solli K. K., Latif Z. E., Benth J. S., Opheim A., Sharma-Haase K. et al. Effectiveness of Injectable Extended-Release Naltrexone vs Daily Buprenorphine-Naloxone for Opioid Dependence: A Randomized Clinical Noninferiority Trial, JAMA Psychiatry 2017: 74: 1197-1205.

34.	Sullivan M., Bisaga A., Pavlicova M., Choi C. J., Mishlen K., Carpenter K. M. et al. Long-Acting Injectable Naltrexone Induction: A Randomized Trial of Outpatient Opioid Detoxification With Naltrexone Versus Buprenorphine, American Journal of Psychiatry 2017: 174: 459-467.

35.	Mishlen K., Barbieri V. A., Sullivan M. A., Bisaga A. Opioid use following an outpatient detoxification and induction onto XR-NTX: Testing the blockade as a predictor of retention in treatment, Drug and Alcohol Dependence 2017: Conference: 2016 Annual Meeting of the College on Problems of Drug Dependence, CPDD 2016. Palm Springs, CA United States. 171: e146.

36.	Korthuis P. T., Lum P. J., Vergara‐Rodriguez P., Ahamad K., Wood E., Kunkel L. E. et al. Feasibility and safety of extended‐release naltrexone treatment of opioid and alcohol use disorder in HIV clinics: a pilot/feasibility randomized trial, Addiction 2017: 112: 1036-1044.

37.	Earley P. H., Zummo J., Memisoglu A., Silverman B. L., Gastfriend D. R. Open-label Study of Injectable Extended-release Naltrexone (XR-NTX) in Healthcare Professionals With Opioid Dependence, Journal of Addiction Medicine 2017: 11: 224-230.

38.	Barnett B., Tebaldi C., McHugh K., Weiss R., Bogunovic O. Clinical experience with injectable naltrexone treatment in sixteen patients with alcohol and opioid use disorder: Retrospective case analysis, American Journal on Addictions 2017: 26(3): 291-292.

39.	Vo H. T., Robbins E., Westwood M., Lezama D., Fishman M. Relapse prevention medications in community treatment for young adults with opioid addiction, Substance Abuse 2016: 37: 392-397.

40.	Stein M. D., Risi M. M., Bailey G. L., Anderson B. J. Linkage to Primary Care for Persons First Receiving Injectable Naltrexone During Inpatient Opioid Detoxification, Journal of Substance Abuse Treatment 2016: 64: 44-46.

41.	Sajid A., Whiteman A., Bell R. L., Greene M. S., Engleman E. A., Chambers R. A. Prescription drug monitoring program data tracking of opioid addiction treatment outcomes in integrated dual diagnosis care involving injectable naltrexone, American Journal on Addictions 2016: 25: 557-564.

42.	Merlo L. J., Campbell M. D., Skipper G. E., Shea C. L., DuPont R. L. Outcomes for Physicians With Opioid Dependence Treated Without Agonist Pharmacotherapy in Physician Health Programs, Journal of Substance Abuse Treatment 2016: 64: 47-54.

43.	Lee J. D., Friedmann P. D., Kinlock T. W., Nunes E. V., Boney T. Y., Hoskinson R. A., Jr. et al. Extended-Release Naltrexone to Prevent Opioid Relapse in Criminal Justice Offenders, New England Journal of Medicine 2016: 374: 1232-1242.

44.	Dayal P., Balhara Y. P. S., Mishra A. K. An open label naturalistic study of predictors of retention and compliance to naltrexone maintenance treatment among patients with opioid dependence, Journal of Substance Use 2016: 21(3): 309-316.

45.	Cousins S. J., Denering L., Crevecoeur-MacPhail D., Viernes J., Sugita W., Barger J. et al. A demonstration project implementing extended-release naltrexone in Los Angeles County, Substance Abuse 2016: 37: 54-62.

46.	Gordon M. S., Kinlock T. W., Vocci F. J., Fitzgerald T. T., Memisoglu A., Silverman B. A Phase 4, Pilot, Open-Label Study of VIVITROL R (Extended-Release Naltrexone XR-NTX) for Prisoners, Journal of Substance Abuse Treatment 2015: 59: 52-58.

47.	Gandhi J. S., Kaur D. Buprenorphine tapering-with and without long acting antagonist, Indian Journal of Psychiatry 2015: 1): S21.

48.	Salimi A., Safari F., Mohajerani S. A., Hashemian M., Kolahi A. A., Mottaghi K. Long-term relapse of ultra-rapid opioid detoxification, Journal of Addictive Diseases 2014: 33: 33-40.

49.	Jhugroo A., Ellayah D., Norman A., Hulse G. Naltrexone implant treatment for buprenorphine dependence - Mauritian case series, Journal of Psychopharmacology 2014: 28(8): 800-803.

50.	Dunn K. E., Defulio A., Everly J. J., Donlin W. D., Aklin W. M., Nuzzo P. A. et al. Employment-based reinforcement of adherence to oral naltrexone treatment in unemployed injection drug users, Exp Clin Psychopharmacol 2013: 21: 74-83.

51.	DeFulio A., Everly J. J., Leoutsakos J. M., Umbricht A., Fingerhood M., Bigelow G. E. et al. Employment-based reinforcement of adherence to an FDA approved extended release formulation of naltrexone in opioid-dependent adults: a randomized controlled trial, Drug & Alcohol Dependence 2012: 120: 48-54.

52.	Chaudhry Z. A., Sultan J., Alam F. Predictors for retention in treatment with a UK community-based naltrexone programme for opioid dependence, Psychiatrist 2012: 36(6): 218-224.

53.	Krupitsky E., Nunes E. V., Ling W., Illeperuma A., Gastfriend D. R., Silverman B. L. Injectable extended-release naltrexone for opioid dependence: a double-blind, placebo-controlled, multicentre randomised trial, Lancet 2011: 377: 1506-1513.

54.	Everly J. J., DeFulio A., Koffarnus M. N., Leoutsakos J. M. S., Donlin W. D., Aklin W. M. et al. Employment‐based reinforcement of adherence to depot naltrexone in unemployed opioid‐dependent adults: A randomized controlled trial, Addiction 2011: 106: 1309-1318.

55.	Day E., Strang J. Outpatient versus inpatient opioid detoxification: A randomized controlled trial, Journal of Substance Abuse Treatment 2011: 40(1): 56-66.

56.	Kunøe N., Lobmaier P., Vederhus J. K., Hjerkinn B., Hegstad S., Gossop M. et al. Retention in naltrexone implant treatment for opioid dependence, Drug and alcohol dependence 2010: 111: 166-169.

57.	Fishman M. J., Winstanley E. L., Curran E., Garrett S., Subramaniam G. Treatment of opioid dependence in adolescents and young adults with extended release naltrexone: preliminary case-series and feasibility, Addiction 2010: 105: 1669-1676.

58.	Mistral W., Wilkinson S., Mastache C., Midgley S., Law F. Efficacy of naltrexone treatment with combined crack and opiate users: A descriptive study of a new treatment service in Bristol, UK, Drugs: Education, Prevention & Policy 2008: 15: 107-119.

59.	Mannelli P., Patkar A. A., Peindl K., Murray H. W., Wu L. T., Hubbard R. Effectiveness of low-dose naltrexone in the post-detoxification treatment of opioid dependence, Journal of Clinical Psychopharmacology 2007: 27: 468-474.

60.	Davison J. W., Sweeney M. L., Bush K. R., Davis Correale T. M., Calsyn D. A., Reoux J. P. et al. Outpatient treatment engagement and abstinence rates following inpatient opioid detoxification, Journal of Addictive Diseases 2006: 25: 27-35.

61.	Marsch L. A., Bickel W. K., Badger G. J., Stothart M. E., Quesnel K. J., Stanger C. et al. Comparison of pharmacological treatments for opioid-dependent adolescents: a randomized controlled trial, Archives of General Psychiatry 2005: 62: 1157-1164.

62.	Hulse G. K., Tait R. J., Comer S. D., Sullivan M. A., Jacobs I. G., Arnold-Reed D. Reducing hospital presentations for opioid overdose in patients treated with sustained release naltrexone implants, Drug & Alcohol Dependence 2005: 79: 351-357.

63.	Digiusto E., Lintzeris N., Breen C., Kimber J., Mattick R. P., Bell J. et al. Short-term outcomes of five heroin detoxification methods in the Australian NEPOD Project, Addictive Behaviors 2005: 30: 443-456.

64.	De Jong C. A. J., Laheij R. J. F., Krabbe P. F. M. General anaesthesia does not improve outcome in opioid antagonist detoxification treatment: A randomized controlled trial, Addiction 2005: 100(2): 206-215.

65.	Arnold-Reed D. E., Hulse G. K. A comparison of rapid (opioid) detoxification with clonidine-assisted detoxification for heroin-dependent persons, Journal of Opioid Management 2005: 1: 17-23.

66.	Tucker T. K., Ritter A. J., Maher C., Jackson H. Naltrexone maintenance for heroin dependence: uptake, attrition and retention, Drug and alcohol review 2004: 23: 299-309.

67.	Rea F., Bell J. R., Young M. R., Mattick R. P. A randomised, controlled trial of low dose naltrexone for the treatment of opioid dependence, Drug & Alcohol Dependence 2004: 75: 79-88.

68.	Malhotra A., Basu D., Mattoo S. K., Gupta N., Malhotra R. Acceptability of naltrexone by patients with opioid dependence: An Indian perspective, Hong Kong Journal of Psychiatry 2003: 13(4): 6-13+32.

69.	Foster J., Brewer C., Steele T. Naltrexone implants can completely prevent early (1-month) relapse after opiate detoxification: a pilot study of two cohorts totalling 101 patients with a note on naltrexone blood levels, Addiction Biology 2003: 8: 211-217.

70.	Summers Z., Stone S. Notes from the clinic: Descriptions of innovative practice a description of opiate detoxification using Buprenorphine in a community setting, Journal of Substance Use 2002: 7(2): 96-99.

71.	Rabinowitz J., Cohen H., Atias S. Outcomes of naltrexone maintenance following ultra rapid opiate detoxification versus intensive inpatient detoxification, American Journal on Addictions 2002: 11: 52-56.

72.	Glasgow N. J., Taylor J., Bell J. R., Young M. R., Bammer G. Accelerated withdrawal from methadone maintenance therapy using naltrexone and minimal sedation: A case-series analysis, Drug and Alcohol Review 2001: 20: 213-221.

73.	Gerra G., Zaimovic A., Giusti F., Di Gennaro C., Zambelli U., Gardini S. et al. Lofexidine versus clonidine in rapid opiate detoxification, Journal of Substance Abuse Treatment 2001: 21: 11-17.

74.	K. Hulse M. B., G. The association between naltrexone compliance and daily supervision, Drug and Alcohol Review 2000: 19: 41-48.

75.	Gerra G., Zaimovic A., Rustichelli P., Fontanesi B., Zambelli U., Timpano M. et al. Rapid opiate detoxication in outpatient treatment: Relationship with naltrexone compliance, Journal of Substance Abuse Treatment 2000: 18(2): 185-191.

76.	Preston K. L., Silverman K., Umbricht A., Dejesus A., Montoya I. D., Schuster C. R. Improvement in naltrexone treatment compliance with contingency management, Drug and Alcohol Dependence 1999: 54(2): 127-135.

77.	Bell J. R., Young M. R., Masterman S. C., Morris A., Mattick R. P., Bammer G. A pilot study of naltrexone-accelerated detoxification in opioid dependence, Medical Journal of Australia 1999: 171: 26-30.

78.	Scherbaum N., Klein S., Kaube H., Kienbaum P., Peters J., Gastpar M. Alternative strategies of opiate detoxification: evaluation of the so-called ultra-rapid detoxification, Pharmacopsychiatry 1998: 31: 205-209.

79.	Roth A., Hogan I., Farren C. Naltrexone plus group therapy for the treatment of opiate-abusing health-care professionals, Journal of Substance Abuse Treatment 1997: 14(1): 19-22.

80.	Rabinowitz J., Cohen H., Tarrasch R., Kotler M. Compliance to naltrexone treatment after ultra-rapid opiate detoxification: An open label naturalistic study, Drug and Alcohol Dependence 1997: 47(2): 77-86.

81.	Cornish J. W., Metzger D., Woody G. E., Wilson D., McLellan A. T., Vandergrift B. et al. Naltrexone pharmacotherapy for opioid dependent federal probationers, Journal of substance abuse treatment 1997: 14: 529-534.

82.	Azatian A., Papiasvilli A., Joseph H. A study of the use of clonidine and naltrexone in the treatment of opioid addiction in the former USSR, Journal of Addictive Diseases 1994: 13: 35-52.

83.	Schifano F., Marra R. Naltrexone for heroin addiction: Encouraging results from Italy, International Journal of Clinical Pharmacology Therapy and Toxicology 1990: 28(4): 144-146.

84.	Kosten T. R., Kleber H. D. Psychoactive substance use disorders: Drug abuse. Outpatient psychiatry: Diagnosis and treatment , 2nd ed, Baltimore, MD: Williams & Wilkins Co; US; 1989, p. 488-509.

85.	Fram D. H., Marmo J., Holden R. Naltrexone treatment--the problem of patient acceptance, Journal of Substance Abuse Treatment 1989: 6: 119-122.

86.	Vining E., Kosten T. R., Kleber H. D. Clinical utility of rapid clonidine-naltrexone detoxification for opioid abusers, British Journal of Addiction 1988: 83(5): 567-575.

87.	Capone T., Brahen L., Condren R. Retention and outcome in a narcotic antagonist treatment program, Journal of Clinical Psychology 1986: 42(5): 825-833.

88.	Washton A. M., Gold M. S., Pottash A. C. Successful use of naltrexone in addicted physicians and business executives, Advances in Alcohol & Substance Abuse 1984: 4: 89-96.

89.	Tennant F. S., Jr., Rawson R. A., Cohen A. J., Mann A. Clinical experience with naltrexone in suburban opioid addicts, Journal of Clinical Psychiatry 1984: 45: 42-45.

90.	Ling W., Wesson D. Naltrexone treatment for addicted health-care professionals: a collaborative private practice experience, The Journal of clinical psychiatry 1984: 45: 46-48.

91.	Greenstein R. A., Arndt I. C., McLellan A. T., O'Brien C. P., Evans B. Naltrexone: a clinical perspective, Journal of Clinical Psychiatry 1984: 45: 25-28.

92.	Resnick R., Washton A., Stone-Washton N. Psychotherapy and naltrexone in opioid dependence, NIDA Res Monogr 1981: 34: 109-115.

93.	Greenstein R. A., O'Brien C. P., McLellan A. T., Woody G. E., Grabowski J., Long M. et al. Naltrexone: a short-term treatment for opiate dependence, American Journal of Drug & Alcohol Abuse 1981: 8: 291-300.

94.	Anton R. F., Hogan I., Jalali B., Riordan C. E., Kleber H. D. Multiple family therapy and naltrexone in the treatment of opiate dependence, Drug and Alcohol Dependence 1981: 8: 157-168.

95.	Grabowski J., O'brien C., Greenstein R., Ternes J., Long M., Steinberg-Donato S. Effects of contingent payment on compliance with a naltrexone regimen, The American journal of drug and alcohol abuse 1979: 6: 355-365.

96.	Resnick R. B., Washton A. M., Thomas M. A., Kestenbaum R. S. Naltrexone in the treatment of opiate dependence, NIDA Res Monogr 1978: 321-332.

97.	Lewis D. C., Mayer J., Hersch R. G., Black R. Narcotic antagonist treatment: clinical experience with naltrexone, International Journal of the Addictions 1978: 13: 961-973.

98.	Hollister L. E., Schwin R. L., Kasper P. Naltrexone treatment of opiate-dependent persons, Drug and Alcohol Dependence 1977: 2: 203-209.

99.	Meyer R., Randall M., Barrington C., Mirin S., Greenberg I. LIMITATATIONS OF AN EXTINCTION APPROACH TO NARCOTIC ANTAGONIST TREATMENT, NALTREXONE 1976: 123.
